# A meta-analysis and expression profiling of DNA repair gene polymorphisms in leukemia

**DOI:** 10.3389/fonc.2026.1777198

**Published:** 2026-04-23

**Authors:** Honghui Ye, Jinyong Fang, Pei Jin

**Affiliations:** 1 Department of Blood Transfusion, Affiliated Jinhua Hospital, Zhejiang University School of Medicine, Jinhua, Zhejiang, China; 2 Department of Hematology, Affiliated Jinhua Hospital, Zhejiang University School of Medicine, Jinhua, Zhejiang, China

**Keywords:** DNA repair, excision repair cross-complementing, leukemia, meta-analysis, polymorphism

## Abstract

**Background:**

Genetic susceptibility is believed to contribute to leukemia development. This study aimed to systematically evaluate the association between DNA repair gene polymorphisms, particularly xeroderma pigmentosum (XP) and excision repair cross-complementing (XRCC) genes, and leukemia risk.

**Methods:**

A comprehensive search of PubMed, Web of Science, Scopus, and Cochrane Library was conducted until December 3, 2025, concerning the association between DNA repair gene polymorphisms and leukemia risk. Gene expressions were performed in Leukemia DB, GEPIA2, and BloodSpot datasets.

**Results:**

Fifty-four studies were included in the meta-analysis. No significant associations were observed for ERCC1 C118T or XPD Asp312Asn across genetic models, whereas ERCC1 8092C>A showed significant associations in homozygous and dominant models. The XPD Lys751Gln polymorphism demonstrated significant associations across all models. Significant associations were identified for XPC Lys939Gln in allelic, homozygous, and recessive models and for XPF Arg415Gln in the heterozygous model. XPG 3507G>C showed no significant association. XRCC1 Arg194Trp, XRCC1 Arg399Gln, and XRCC3 Thr241Met polymorphisms were significantly associated with leukemia risk, while XRCC1 Arg280His was not. Gene expression analyses revealed subtype-specific alterations in DNA repair genes across leukemia datasets.

**Conclusions:**

Several DNA repair polymorphisms, including *XPD Lys751Gln, XPC Lys939Gln, XRCC1 Arg194Trp, XRCC1 Arg399Gln*, and *XRCC3 Thr241Met*, are associated with increased leukemia susceptibility. Differential expression patterns of DNA repair genes across leukemia subtypes suggest functional heterogeneity and highlight their potential relevance in risk stratification and biomarker development.

## 1 Introduction

Leukemia is a malignancy of the blood, marked by the transformation of hematopoietic progenitors and widespread infiltration of the bone marrow (1). The primary types of leukemia are acute lymphoblastic leukemia (ALL), acute myeloid leukemia (AML), chronic lymphocytic leukemia (CLL), and chronic myeloid leukemia (CML) (2). In 2020, leukemia represented approximately 2.5% of all new cancer cases and 3.1% of cancer-related deaths worldwide (3). Risk factors for leukemia include smoking, exposure to certain chemicals, previous chemotherapy, radiation exposure, rare congenital diseases, specific blood disorders, family history, age, and gender (4–7). Further, longitudinal studies are needed to understand the reasons behind these epidemiological trends and to provide more insights into the specific etiology and prognosis of different leukemia subtypes (8). However, only a small fraction of individuals exposed to these risk factors develop leukemia, indicating that genetic factors may play a significant role in leukemia carcinogenesis (9, 10). The effectiveness of treatments varies depending on the type of leukemia and the patient’s condition (11–14).

DNA repair pathways, often called the guardians of the genome, play a crucial role in protecting cells from frequent damage that can lead to DNA breaks (15). These pathways operate through coordinated enzymatic steps including damage recognition, excision of altered nucleotides, DNA resynthesis, and ligation, and may be functionally influenced by genetic polymorphisms that alter repair efficiency (16). DNA repair is mediated through several highly coordinated pathways that maintain genomic stability by correcting distinct types of DNA damage (17). Nucleotide excision repair (NER) primarily removes bulky helix-distorting lesions such as UV-induced photoproducts and chemical adducts, thereby preventing transcriptional arrest and replication errors (18). In contrast, base excision repair (BER) and single-strand break repair pathways correct oxidative DNA damage, alkylation, and spontaneous base loss, which frequently occur during normal cellular metabolism and exposure to environmental agents (19). Functional polymorphisms within genes encoding key repair proteins may alter enzymatic activity, protein stability, or DNA-binding affinity, potentially reducing repair efficiency. Impaired repair capacity can lead to the accumulation of somatic mutations, chromosomal instability, and dysregulated hematopoietic proliferation, thereby contributing to leukemogenesis (20).

Among these mechanisms, NER is particularly significant. NER serves as a vital defense against carcinogenesis by removing a wide range of structurally diverse DNA lesions (21, 22). Enzymes within this pathway have been implicated in cancer development (23). The NER system is composed of approximately 30 genes (24), including seven key genes (*XPA–XPG*) associated with the genetic disorder Xeroderma pigmentosum (XP) (25, 26). Furthermore, recent meta-analyses suggest that polymorphisms in XP genes may contribute to the risk of various malignancies (27–30).

Members of the X-ray repair cross-complementary (XRCC) gene family function predominantly within base excision repair and single-strand break repair pathways, where they act as scaffolding or enzymatic cofactors facilitating efficient repair of oxidative and endogenous DNA lesions (31). The XRCC gene family comprises an important class of DNA damage repair genes that encode proteins that play important roles in DNA single-strand breakage and DNA base damage repair (32, 33). The dysfunction of the *XRCC* gene family is associated with the development of various tumors (33). Most of these genes were identified through their ability to correct DNA damage hypersensitivity in rodent cell lines and they represent components of several different repair pathways (34).

Three meta-analyses (three reported in 2014 and one in 2018) have identified an association between *XPD Lys751Gln* polymorphisms and the risk of leukemia (35–37), with the most recent meta-analysis incorporating 14 studies (35). No meta-analyses have examined the association between other polymorphisms (*excision repair cross-complementing 1 (ERCC1), XPC, XPD Asp312Asn, XPF/ERCC4*, and *XPG/ERCC5*) and leukemia risk. In addition, one meta-analysis (38) reported the association of *XRCC1* polymorphisms with the risk of leukemia in 2013 and two meta-analyses reported *Arg194Trp* (39) and *Arg399Gln* (40) with the risk of hematological malignancies. One meta-analysis in 2021, reported *XRCC3 Thr241Met* polymorphism in leukemia patients (41). Therefore, this study aimed to conduct a comprehensive systematic review and meta-analysis to evaluate the association of *XPD Lys751Gln, XRCC1 Arg194Trp, XRCC1 Arg399Gln, XRCC1 Arg280His*, and *XRCC3 Thr241Met* polymorphisms with leukemia susceptibility, including more studies than previous analyses. In addition, we assessed *ERCC1, XPC, XPD Asp312Asn, XPF/ERCC4*, and *XPG/ERCC5* polymorphisms for the first time based on our knowledge, and integrated multi-dataset gene expression analyses to explore subtype-specific transcriptional patterns in leukemia.

## 2 Materials and methods

This study was not registered in any public databases. This meta-analysis was conducted following the PECO model to clearly define the research question. The Population (P) included patients diagnosed with leukemia of any subtype (e.g., acute lymphoblastic leukemia, acute myeloid leukemia, chronic leukemia). The Exposure (E) was the presence of *XRCC1* polymorphisms (*Arg194Trp, Arg399Gln, Arg280His*) or the *XRCC3 Thr241Met* polymorphism. The Comparison (C) consisted of cancer-free individuals, including population-based healthy controls and hospital-based non-cancer patients, depending on the original study. The Outcome (O) was the risk of developing leukemia, assessed using effect estimates such as odds ratios. This meta-analysis was conducted in accordance with the Preferred Reporting Items for Systematic Reviews and Meta-Analyses (PRISMA) guidelines (42).

### 2.1 Literature search

A systematic literature search was conducted in PubMed/Medline, Scopus, Cochrane Library, and Web of Science databases for studies published up to December 3, 2025, without language restriction. The search terms incorporated various synonyms and abbreviations for “xeroderma pigmentosum” and “excision repair cross-complementing” genes in combination with “leukemia”. To ensure comprehensive coverage, reference lists of retrieved articles were manually screened. Two authors (H.Y. and J.F.) independently reviewed the search and selection process, and any discrepancies were resolved by consulting a third author (P.J.).

To verify claims of novelty, in addition to screening primary genetic association studies, we conducted a targeted search for existing meta-analyses on each polymorphism and leukemia susceptibility. No published meta-analysis on *ERCC1, XPC, XPD Asp312Asn, XPF/ERCC4*, or *XPG/ERCC5* polymorphisms in leukemia was found through December 3, 2025.

### 2.2 Eligibility criteria

Studies were considered eligible if they met the following criteria: (a) original human case-control studies investigating polymorphisms in *ERCC1, XPC, XPD/ERCC2, XPF/ERCC4, XPG/ERCC5, XRCC1*, or *XRCC3* in individuals diagnosed with leukemia compared to control subjects; (b) keukemia diagnosis confirmed through clinical or pathological criteria, with cases representing primary leukemia patients and not individuals with other concurrent malignancies unless leukemia-specific data were reported separately; and (c) control groups consisting of either healthy individuals or subjects without any malignancy.

Exclusion criteria included: (a) non-original studies (reviews, meta-analyses, editorials, or animal studies); (b) studies lacking complete data or a control group; (c) duplicate publications; (d) cases undergoing treatment at the time of study participation; and (e) studies including participants with other malignancies or severe concurrent systemic diseases without separate analysis for leukemia cases.

### 2.3 Study selection, data extraction and quality assessment

Two authors (H.Y. and P.J.) screened titles, abstracts, and full texts to identify eligible studies and extracted data using a standardized form. Any discrepancies between the reviewers were resolved through discussion and consensus. If consensus could not be reached, a third senior reviewer (J.F.) was consulted to make the final decision.

The methodological quality of the included studies was evaluated using the Newcastle-Ottawa Scale (NOS) (43). Two reviewers (P.J. and J.F.) independently assessed the scores for each study, and discrepancies were resolved through discussion. The NOS assigns scores ranging from 0 to 9, with higher scores indicating better study quality.

### 2.4 Statistical analysis

The odds ratio (OR) with a 95% confidence interval (CI) was calculated to assess the association between gene polymorphisms and leukemia risk using Review Manager 5.3 (RevMan 5.3). A significance threshold of *p* < 0.05 was set for all analyses. To evaluate heterogeneity across studies, both the I² statistic and the Chi-square test were used. The I² statistic quantifies the proportion of variability attributed to true heterogeneity rather than random chance, while the Chi-square test detects the overall presence of heterogeneity. Substantial heterogeneity was considered when p < 0.10 and I² > 50%, in which case a random-effects model (DerSimonian, 2015 #33) was applied. If heterogeneity was low or moderate, a fixed-effect model (Mantel, 1959 #34) was used instead. To address heterogeneity, we performed subgroup analyses based on ethnicity, leukemia subtype, age group, and study quality.

Meta-regression analysis was conducted using a random-effects model to explore potential sources of heterogeneity by examining study-level covariates that could influence effect sizes. Sensitivity analyses were performed by systematically omitting individual studies or modifying analytical methods to assess the robustness of the findings. Both analyses were carried out using Comprehensive Meta-Analysis version 3.0 (CMA 3.0). Additionally, publication bias was evaluated using Begg’s and Egger’s tests. Begg’s test utilizes rank correlation (Kendall’s tau) to assess the relationship between effect estimates and their variances, while Egger’s test employs regression analysis to detect small-study effects. A two-sided p-value < 0.10 was considered indicative of potential publication bias, with all analyses conducted in CMA 3.0.

To minimize the risk of false-positive or false-negative findings in meta-analyses (44), Trial Sequential Analysis (TSA) was performed using TSA software (version 0.9.5.10 beta) (45). TSA estimates the Required Information Size (RIS), which represents the optimal sample size needed to achieve reliable conclusions while controlling for Type I (α = 5%) and Type II (β = 80%) errors. If the Z-curve reaches or exceeds the RIS and crosses the sequential monitoring boundary, the results are considered conclusive.

Hardy–Weinberg equilibrium (HWE) in control groups was evaluated using the chi-square test. Studies with control groups deviating from HWE (p < 0.05) were considered to have potential methodological limitations. To assess the robustness of pooled estimates, additional sensitivity analyses were performed after excluding studies violating HWE assumptions.

### 2.5 Functional analysis

To examine the functional interactions among the studied genes, the protein-protein interaction (PPI) network was analyzed using the STRING database (https://string-db.org/, accessed on July 20, 2024). The analysis was restricted to *Homo sapiens* to ensure relevance to human biology. An interaction score threshold of 0.400, corresponding to the medium confidence level, was applied to filter interactions.

### 2.6 Gene expression analysis in leukemia via leukemiaDB, GEPIA2, and bloodSpot

Gene expression profiling was performed using LeukemiaDB (http://guolab.wchscu.cn/LeukemiaDB), GEPIA2 (http://gepia2.cancer-pku.cn), and BloodSpot (https://www.fobinf.com) to investigate transcriptional alterations associated with leukemia. LeukemiaDB, a curated transcriptomic resource comprising over 3000 RNA-seq samples from 14 leukemia subtypes, 53 cell lines, and 92 normal controls, was utilized to explore subtype-specific expression patterns of protein-coding and non-coding RNAs. Data normalization was conducted using ComBat to correct for batch effects, and subtype-enriched genes were identified via SEGtool. Complementarily, GEPIA2 was employed for differential expression analysis between AML patients (TCGA-LAML dataset) and matched normal tissues from the GTEx project, applying a |Log2FC| cutoff of ≥1 and p-value threshold of ≤0.01 to establish statistical significance. Visualization enhancements such as jitter sizing (0.4) were applied to refine interpretability. This dual-platform approach enabled robust identification of dysregulated genes and provided stratified insights into leukemia subtype heterogeneity.

Within BloodSpot’s MILE dataset, expression profiling of *ERCC1, ERCC2, ERCC4, ERCC5, XRCC1*, and *XRCC3* revealed distinct transcriptional patterns across leukemia subtypes, highlighting their expression and correlations across leukemia subtypes from the MILE dataset.

### 2.7 Integrative functional and transcriptomic analyses

To provide biological context for DNA repair gene polymorphisms identified in the meta-analysis, integrative transcriptomic and functional analyses were performed using publicly available leukemia gene expression resources and bioinformatic platforms. These analyses were conducted as exploratory functional annotations rather than primary statistical endpoints.

#### 2.7.1 Gene expression profiling across leukemia subtypes

Gene expression patterns of DNA repair genes demonstrating significant pooled genetic associations were explored using publicly available leukemia transcriptomic databases, including GEPIA2, BloodSpot, and LeukemiaDB. Expression profiles were examined across multiple leukemia subtypes (AML, ALL, CML, and CLL) to identify differences in transcriptional levels between leukemia samples and reference controls when available. Analyses were performed using default platform parameters, and findings were interpreted qualitatively to identify consistent patterns of differential gene expression.

#### 2.7.2 Functional pathway enrichment analysis

DNA repair genes included in the meta-analysis were subjected to functional enrichment analysis to evaluate their involvement in biological pathways and molecular processes. Gene Ontology (GO) biological process and Kyoto Encyclopedia of Genes and Genomes (KEGG) pathway enrichment analyses were conducted using publicly available enrichment tools such as DAVID, Enrichr, or g:Profiler. Pathways with adjusted p-values < 0.05 were considered significantly enriched.

#### 2.7.3 Protein–protein interaction network analysis

Protein–protein interaction (PPI) networks among DNA repair proteins were evaluated using the STRING database (Search Tool for the Retrieval of Interacting Genes/Proteins). Interaction networks were constructed to assess functional connectivity and identify central hub proteins within DNA repair pathways. Default confidence score thresholds were applied, and network topology was visually assessed to determine key interaction clusters and pathway convergence.

## 3 Results

### 3.1 Study selection

**Figure 1:**
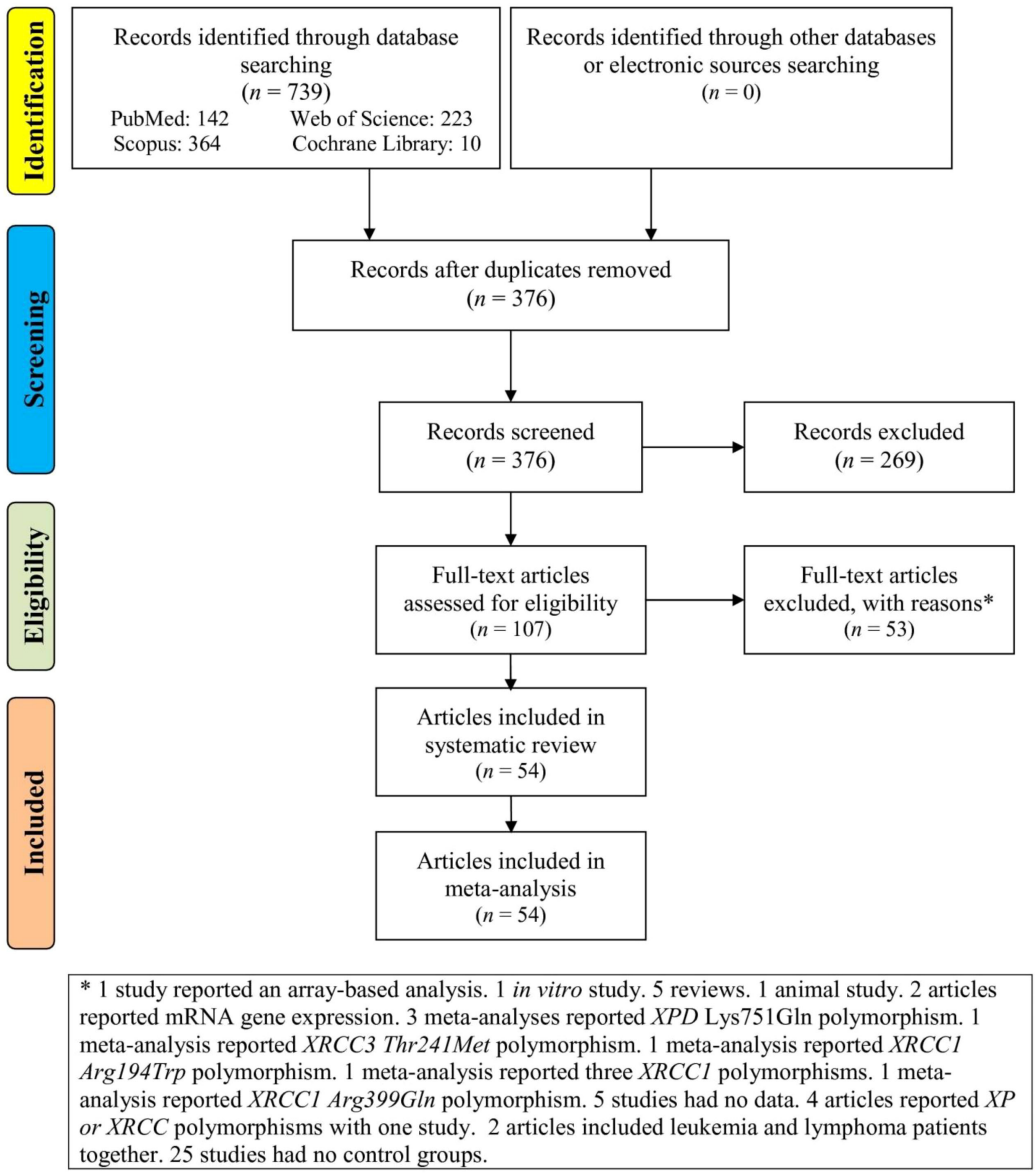
Flowchart of the study selection.

**Figure 1** provides the study selection process for the systematic review or meta-analysis. The flowchart starts with records identified through database searching (739 records) and other sources (0 records). Specific databases mentioned include PubMed (142 records), Web of Science (223 records), Scopus (364 records), and Cochrane Library (10 records). After removing duplicates, 376 records remain. All 376 records are screened. 269 records are excluded at this stage. 107 full-text articles are assessed for eligibility. 53 articles are excluded for various reasons. At last, 54 articles (46–99) are included in the systematic review and the same articles are included in the meta-analysis.

### 3.2 Studies’ characteristics

**Table 1** summarizes the characteristics of studies included in the meta-analysis. Studies are from various countries with different ethnic groups (Caucasian, Asian, and Mixed). The cancer subtypes studied include CML, CLL, AML, and ALL. Most studies focus on adults, with some including children or having no age limit. Controls are either population-based (PB) or hospital-based (HB). The primary method used is PCR-RFLP, with a few studies using TaqMan. Quality scores range from 6 to 9, indicating the methodological quality of the studies (**Table 2**).

**Table 1:** Characteristics of the studies included in the meta-analysis.

The first author, publication year	Country	Ethnicity	Subtype	Age group	Control source	Genotyping method
Abdalhabib, 2021 (46)	Saudi Arabia	Caucasian	CML	Adult	PB	PCR-RFLP
Abramenko, 2012 (47)	Ukraine	Caucasian	CLL	Adult	HB	PCR-RFLP
Allan, 2004 (48)	UK	Caucasian	AML	Adult	HB	PCR-RFLP
Annamaneni, 2013 (49)	India	Asian	CML	Adult	PB	PCR-RFLP
Banescu, 2013 (50)	Romania	Caucasian	AML	Adult	PB	PCR-RFLP
Banescu, 2014 (51)	Romania	Caucasian	CML	Adult	PB	PCR-RFLP
Banescu, 2016 (52)	Romania	Caucasian	AML	Adult	PB	PCR-RFLP
Batar, 2009 (53)	Turkey	Caucasian	ALL	Child	PB	PCR-RFLP
Bhatla, 2008 (54)	USA	Mixed	AML	Child	PB	TaqMan
Canalle, 2011 (55)	Brazil	Mixed	ALL	Child	HB	PCR-RFLP
Deligezer, 2007 (56)	Turkey	Caucasian	AML and CML	≥ 16 years	PB	PCR
Dincer, 2015 (57)	Turkey	Caucasian	ALL	Child	HB	PCR-RFLP
Douzi, 2015 (58)	Tunisia	Mixed	ALL	No age limit	PB	PCR-RFLP
Duman, 2012 (59)	Turkey	Caucasian	CLL	Adult	PB	PCR
El-Din, 2012 (60)	Egypt	Caucasian	AML	Adult	PB	PCR-RFLP
Erčulj, 2012 (61)	Slovenia	Caucasian	ALL	Child	PB	TaqMan
Fekry, 2018 (62)	Egypt	Caucasian	Leukemia	Adult and Child	HB	PCR-RFLP
Ganster, 2009 (63)	Austria	Caucasian	CLL	Adult	PB	PCR-RFLP
Goričar, 2015 (64)	Slovenia	Caucasian	ALL	Child	PB	PCR
Hamdy, 2011 (65)	Egypt	Caucasian	AML	≥ 14 years	PB	Direct sequencing
Joseph, 2005 (66)	India	Asian	ALL	Child	HB	PCR-RFLP
Kagita, 2018 (67)	India	Asian	CML	Adult	HB	PCR-RFLP
Kim, 2012 (68)	Korea	Asian	AML	≥ 15 years	PB	PCR-RFLP
Lakkireddy, 2021 (69)	India	Asian	CML	Adult	PB	PCR-RFLP
Li, 2011 (70)	China	Asian	ALL	Child	PB	PCR-RFLP
Liu, 2011 (71)	China	Asian	AML	Adult	PB	PCR-RFLP
Matullo, 2006 (72)	Europe	Caucasian	Unclassified	Adult	PB	TaqMan
Mehta, 2006 (73)	USA	Mixed	AML	Child	HB	TaqMan
Meza-Espinoza, 2009 (74)	Mexico	Mixed	ALL	Child	PB	PCR-RFLP
Miao, 2015 (75)	China	Asian	AML	Adult	PB	TaqMan
Mortada, 2019 (76)	Egypt	Caucasian	AML	Adult	PB	PCR-RFLP
Mutlu, 2015 (77)	Turkey	Caucasian	CLL	Not reported	HB	TaqMan
Ozcan, 2011 (78)	Turkey	Caucasian	AML	Adult	PB	PCR-RFLP
Ozdilli, 2021 (79)	Turkey	Caucasian	CML	Adult	PB	PCR-RFLP
Pakakasama, 2007 (80)	Thailand	Asian	ALL	Child	PB	PCR-RFLP
Pei, 2018 (81)	Taiwan	Asian	Leukemia	Child	PB	PCR-RFLP
Salimizand, 2016 (82)	Iran	Caucasian	CML	Adult	PB	PCR
Santhi, 2017 (83)	Canada	Mixed	AML	Adult	HB	PCR-RFLP
Seedhouse, 2002 (84)	UK	Caucasian	AML	Adult	PB	PCR-RFLP
Seedhouse, 2004 (85)	UK	Caucasian	AML	Adult	PB	PCR-RFLP
Shi, 2011 (86)	China	Asian	AML	Adult	PB	PCR-RFLP
Smolkova, 2014 (87)	Germany	Caucasian	ALL	Child	PB	TaqMan
Sorour, 2013 (88)	Egypt	Mixed	AML	Adult	HB	PCR-RFLP
Stanczyk, 2011 (89)	Poland	Caucasian	ALL	Child	PB	PCR-RFLP
Tumer, 2010 (90)	Turkey	Caucasian	ALL	Child	PB	PCR-RFLP
Wang, 2006 (91)	China	Asian	ALL	Child	HB	PCR-RFLP
Wang, 2009 (92)	China	Asian	AML	Adult	HB	PCR-RFLP
Wilson, 2023 (93)	Egypt	Caucasian	ALL	Child	PB	PCR-RFLP
Yalcin, 2014 (94)	Romania	Caucasian	CML	Adult	PB	PCR-RFLP
Yang, 2011 (95)	China	Asian	AML	Adult	PB	TaqMan
Zehtab, 2022 (96)	Iran	Caucasian	ALL	Child	HB	Real-time PCR
Zhang, 2009 (97)	China	Asian	ALL	Child	PB	PCR-RFLP
Zhu, 2005 (98)	China	Asian	Acute leukemia	Child	PB	PCR-RFLP
Zhu, 2008 (99)	China	Asian	Leukemia	Child	HB	PCR-RFLP

CML, Chronic Myeloid Leukemia; CLL, Chronic Lymphocytic Leukemia; AML, Acute Myeloid Leukemia; ALL, Acute Lymphoblastic Leukemia; PB, Population-based; HB, Hospital-based; PCR-RFLP, Polymerase chain reaction-restriction fragment length polymorphism.

**Table 2:** Quality score of each study with maximum 9 points.

Study	Selection	Comparability	Exposure	Total score
Abdalhabib, 2021 (46)	★★★	★★	★★★	8
Abramenko, 2012 (47)	★★★	★★	★★★	8
Allan, 2004 (48)	★★★	★★	★★★	8
Annamaneni, 2013 (49)	★★★★	★★	★★★	9
Banescu, 2013 (50)	★★★	★	★★★	7
Banescu, 2014 (51)	★★★★	★★	★★★	9
Banescu, 2016 (52)	★★★★	★★	★★★	9
Batar, 2009 (53)	★★★★	★★	★★★	9
Bhatla, 2008 (54)	★★★★	★★	★★★	9
Canalle, 2011 (55)	★★★	★	★★★	7
Deligezer, 2007 (56)	★★★	★	★★★	7
Dincer, 2015 (57)	★★	★	★★★	6
Douzi, 2015 (58)	★★★	★★	★★★	8
Duman, 2012 (59)	★★★	★★	★★★	8
El-Din, 2012 (60)	★★★	★★	★★★	8
Erčulj, 2012 (61)	★★★	★	★★★	7
Fekry, 2018 (62)	★★★	★★	★★★	8
Ganster, 2009 (63)	★★★★	★★	★★★	9
Goričar, 2015 (64)	★★★★	★★	★★★	9
Hamdy, 2011 (65)	★★	★	★★★	6
Joseph, 2005 (66)	★★★	★★	★★★	8
Kagita, 2018 (67)	★★★	★★	★★★	8
Kim, 2012 (68)	★★★	★	★★★	7
Lakkireddy, 2021 (69)	★★★★	★★	★★★	9
Li, 2011 (70)	★★★	★	★★★	7
Liu, 2011 (71)	★★★	★	★★★	7
Matullo, 2006 (72)	★★★★	★★	★★★	9
Mehta, 2006 (73)	★★★★	★★	★★★	9
Meza-Espinoza, 2009 (74)	★★★	★	★★★	7
Miao, 2015 (75)	★★★★	★★	★★★	9
Mortada, 2019 (76)	★★★★	★★	★★★	9
Mutlu, 2015 (77)	★★★	★★	★★★	8
Ozcan, 2011 (78)	★★★	★	★★★	7
Ozdilli, 2021 (79)	★★★	★	★★★	7
Pakakasama, 2007 (80)	★★★	★	★★★	7
Pei, 2018 (81)	★★★	★	★★★	7
Salimizand, 2016 (82)	★★★★	★★	★★★	9
Santhi, 2017 (83)	★★★	★★	★★★	8
Seedhouse, 2002 (84)	★★★	★★	★★★	8
Seedhouse, 2004 (85)	★★★	★★	★★★	8
Shi, 2011 (86)	★★★★	★★	★★★	9
Smolkova, 2014 (87)	★★★★	★★	★★★	9
Sorour, 2013 (88)	★★★	★★	★★★	8
Stanczyk, 2011 (89)	★★★	★★	★★★	8
Tumer, 2010 (90)	★★★	★★	★★★	8
Wang, 2006 (91)	★★★	★★	★★★	8
Wang, 2009 (92)	★★★	★★	★★★	8
Wilson, 2023 (93)	★★★	★★	★★★	8
Yalcin, 2014 (94)	★★★	★★	★★★	8
Yang, 2011 (95)	★★★	★	★★★	7
Zehtab, 2022 (96)	★★★	★★	★★★	8
Zhang, 2009 (97)	★★★	★★	★★★	8
Zhu, 2005 (98)	★★★	★	★★★	7
Zhu, 2008 (99)	★★★	★	★★★	7

Each star means one point/score.

### 3.3 Genotype distribution

**Table 3** summarizes the distribution of genotypes for seven polymorphisms across various studies. Across studies evaluating *XPD/ERCC2 Lys751Gln*, heterozygous genotypes were most frequent in both cases and controls, while homozygous variant genotypes were relatively less common. HWE was generally maintained across control groups, indicating acceptable genetic distribution quality.

**Table 3:** Distribution of genotypes of seven polymorphisms of *XP*,.

The first author, publication year	Case/control	*ERCC1 C118T (Asn118Asn, 19007 C/T, rs11615)*						*p*-value of HWE
		CC	CT	TT	CC	CT	TT	
Kagita, 2018 (67)	174/174	51	94	29	49	87	38	0.957
Matullo, 2006 (72)	169/1093	26	82	61	188	503	402	0.156
The first author, publication year	Case/control	*ERCC1 8092C>A (rs3212986)*						*p*-value of HWE
		AA	AC	CC	AA	AC	CC	
Kim, 2012 (68)	410/1698	128	207	75	440	863	395	0.478
Wang, 2006 (91)	183/190	15	57	111	13	83	94	0.350
The first author, publication year	Case/control	*XPD/ERCC2 Lys751Gln* (*35931 A/C*, *rs13181*)						*p*-value of HWE
		Lys/Lys	Lys/Gln	Gln/Gln	Lys/Lys	Lys/Gln	Gln/Gln	
Abdalhabib, 2021 (46)	150/150	94	36	20	112	30	8	**0.005**
Abramenko, 2012 (47)	166/103	54	89	23	47	44	12	0.729
Allan, 2004 (48)	474/696	180	216	78	293	299	104	**0.056**
Bănescu, 2014 (51)	165/180	51	77	28	82	79	19	0.996
Bănescu, 2016 (52)	108/163	34	59	15	88	47	28	**< 0.001**
Batar, 2009 (53)	70/75	26	33	11	27	35	13	0.775
Canalle, 2011 (55)	162/223	72	78	12	105	101	17	0.276
Dincer, 2015 (57)	30/30	11	12	7	9	17	4	0.364
Douzi, 2015 (58)	85/206	33	43	9	92	93	21	0.723
Ganster, 2009 (63)	444/444	157	222	65	186	194	64	0.248
Kim, 2012 (68)	413/1700	363	47	3	1516	179	5	0.906
Matullo, 2006 (72)	169/1094	70	79	20	397	504	193	0.133
Mehta, 2006 (73)	313/432	116	142	55	183	194	55	0.748
Özcan, 2011 (78)	35/100	18	16	1	41	41	18	0.179
Ozdilli, 2021 (79)	62/70	28	24	10	37	24	9	0.124
Pakakasama, 2007 (80)	108/317	87	19	2	260	56	1	0.263
Santhi, 2017 (83)	208/211	86	89	33	139	61	11	0.216
Seedhouse, 2002 (84)	122/73	44	59	19	30	32	11	0.610
Shi, 2011 (86)	303/554	243	58	2	480	70	4	0.415
Sorour, 2013 (88)	90/60	33	45	12	27	30	3	0.140
The first author, publication year	Case/control	*XPD/ERCC2 Asp312Asn (23591 G/A, rs1799793)*						*p*-value of HWE
		Asp/Asp	Asp/Asn	Asn/Asn	Asp/Asp	Asp/Asn	Asn/Asn	
Batar, 2009 (53)	70/75	26	31	13	26	36	13	0.929
Canalle, 2011 (55)	113/150	62	47	4	84	57	9	0.870
Matullo, 2006 (72)	169/1094	71	76	22	418	506	170	0.411
Pakakasama, 2007 (80)	108/347	97	11	0	272	43	2	0.832
The first author, publication year	Case/control	*XPC Lys939Gln (2920A > C, rs2228001)*						*p*-value of HWE
		Lys/Lys	Lys/Gln	Gln/Gln	Lys/Lys	Lys/Gln	Gln/Gln	
Bănescu, 2016 (52)	108/163	21	63	24	36	88	39	0.306
Douzi, 2015 (58)	206/206	61	104	41	85	98	23	0.507
Kim, 2012 (68)	413/1700	153	186	74	650	812	238	0.539
Lakkireddy, 2021 (69)	212/212	111	82	19	112	82	18	0.588
The first author, publication year	Case/control	XPG/ERCC5 *Asp1104His (3507G > C, rs17655)*						*p*-value of HWE
		Asp/Asp	Asp/His	His/His	Asp/Asp	Asp/His	His/His	
Bănescu, 2016 (52)	108/163	83	21	4	97	58	8	0.858
Douzi, 2015 (58)	206/206	87	96	23	89	94	23	0.807
The first author, publication year	Case/control	*XPF/ERCC4 Arg415Gln (-673C > T, rs1800067)*						*p*-value of HWE
		Arg/Arg	Arg/Gln	Gln/Gln	Arg/Arg	Arg/Gln	Gln/Gln	
Bănescu, 2016 (52)	108/163	57	35	16	71	76	16	0.504
Ganster, 2009 (63)	265/265	229	31	5	220	41	4	0.204

HWE, Hardy-Weinberg equilibrium. Bold number donates statistical significance (*p* < 0.05).

**Table 4** summarizes the distribution of genotypes for seven polymorphisms across various studies that *XRCC1 Arg194Trp* with 22 studies, *XRCC1 Arg399Gln* with 31 studies, *XRCC1 Arg280His* with 8 studies, and *XRCC3 Thr241Met* with 21 studies.

**Table 4:** Distribution of genotypes of four polymorphisms of *XRCC*.

The first author, publication year	Case/control	*XRCC1 Arg194Trp (rs1799782)*						HWE
		Case			Control			
		Arg/Arg	Arg/Trp	Trp/Trp	Arg/Arg	Arg/Trp	Trp/Trp	
Abdalhabib, 2021 (100)	186/186	168	16	2	171	12	3	**< 0.001**
Annamaneni, 2013 (49)	350/350	12	81	257	7	113	230	0.103
Banescu, 2014 (51)	69/147	28	29	12	94	45	8	0.401
Batar, 2009 (53)	70/75	52	16	2	64	11	0	0.493
Canalle, 2011 (55)	201/361	168	32	1	298	59	4	0.577
Dincer, 2015 (57)	30/30	22	8	0	28	2	0	0.850
Duman, 2012 (59)	73/50	64	8	1	41	9	0	0.484
Erčulj, 2012 (61)	20/39	16	3	1	32	6	1	0.305
Fekry, 2018 (62)	48/48	2	28	18	31	9	8	**< 0.001**
Ganster, 2009 (63)	439/439	371	63	5	389	45	5	**0.007**
Goričar, 2015 (64)	121/184	103	18		157	27		–
Joseph, 2005 (66)	117/117	77	32	8	91	22	4	0.085
Kim, 2012 (68)	50/2196	213	285	62	992	989	215	0.164
Li, 2011 (70)	280/200	152	96	32	100	82	18	0.839
Matullo, 2006 (72)	169/1094	145	23	1	951	141	2	0.171
Meza-Espinoza, 2009 (74)	120/120	80	34	6	86	31	3	0.917
Mortada, 2019 (76)	92/92	67	12	13	70	18	4	0.063
Pakakasama, 2007 (80)	108/317	62	44	2	150	145	22	0.097
Salimizand, 2016 (82)	70/140	12	5	53	91	14	35	**< 0.001**
Seedhouse, 2002 (84)	126/87	112	14	0	78	7	2	**0.002**
Shi, 2011 (86)	307/558	236	66	5	445	109	4	0.335
Tumer, 2010 (90)	167/190	140	27	0	159	26	5	**0.005**
Yalcin, 2014 (94)	156/180	119	31	6	129	45	6	0.405
Zehtab, 2022 (96)	50/50	47	0	3	45	0	5	**< 0.001**
Zhu, 2005 (98)	63/66	25	38		30	36		–
Zhu, 2008 (99)	105/108	52	43	10	42	48	18	0.499
The first author, publication year	Case/control	*XRCC1 Arg399Gln (rs25487)*						*p*-value of HWE
		Case			Control			
		Arg/Arg	Arg/Gln	Gln/Gln	Arg/Arg	Arg/Gln	Gln/Gln	
Abdalhabib, 2021 (100)	186/186	131	9	46	153	6	27	**< 0.001**
Abramenko, 2012 (47)	169/94	67	82	20	38	41	15	0.484
Annamaneni, 2013 (49)	350/350	79	191	80	61	235	54	**< 0.001**
Banescu, 2014 (51)	69/147	33	23	13	91	46	10	0.219
Batar, 2009 (53)	70/75	24	37	9	24	37	14	0.968
Canalle, 2011 (55)	201/361	112	72	17	186	152	23	0.272
Deligezer, 2007 (56)	254/226	103	121	30	96	101	29	0.762
Dincer, 2015 (57)	30/30	11	14	5	7	19	4	0.125
Duman, 2012 (59)	73/50	7	50	16	19	26	5	0.36
El-Din, 2012 (60)	40/20	20	16	4	16	2	2	**0.006**
Erčulj, 2012 (61)	20/39	11	7	2	22	13	4	0.339
Ganster, 2009 (63)	429/429	173	192	64	184	193	52	0.899
Goričar, 2015 (64)	121/184	64	57		79	105		–
Joseph, 2005 (66)	117/117	55	46	16	75	33	9	0.061
Kim, 2012 (68)	415/1698	234	155	26	914	693	91	**0.005**
Matullo, 2006 (72)	169/1094	67	74	28	484	482	128	0.632
Meza-Espinoza, 2009 (74)	120/120	57	51	12	65	47	8	0.899
Mortada, 2019 (76)	92/92	56	25	11	55	28	9	0.071
Mutlu, 2015 (77)	25/30	6	11	8	10	8	12	**0.011**
Ozcan, 2011 (78)	45/100	22	22	1	42	43	15	0.469
Ozdilli, 2021 (79)	62/70	25	22	15	25	24	21	**0.009**
Pakakasama, 2007 (80)	108/317	39	60	9	175	124	18	0.514
Santhi, 2017 (83)	211/211	55	140	16	103	79	29	**0.033**
Seedhouse, 2002 (84)	167/178	70	69	28	55	76	47	0.054
Shi, 2011 (86)	306/558	173	114	19	316	213	29	0.369
Sorour, 2013 (88)	90/60	54	27	9	33	27	0	**0.024**
Stanczyk, 2011 (89)	97/131	34	45	18	50	57	24	0.281
Tumer, 2010 (90)	167/190	63	77	27	92	78	20	0.569
Yalcin, 2014 (94)	156/180	71	69	16	91	73	16	0.804
Zhu, 2005 (98)	63/66	18	45		34	32		–
Zhu, 2008 (99)	105/108	45	44	16	62	39	7	0.796
The first author, publication year	Case/control	*XRCC1 Arg280His (rs25489)*						*p*-value of HWE
		Case			Control			
		Arg/Arg	Arg/His	His/His	Arg/Arg	Arg/His	His/His	
Abdalhabib, 2021 (100)	186/186	174	11	1	173	12	1	0.135
Annamaneni, 2013 (49)	350/350	346	4	0	338	11	1	**0.009**
Joseph, 2005 (66)	117/117	76	38	3	85	30	2	0.726
Meza-Espinoza, 2009 (74)	120/120	87	31	2	88	31	1	0.328
Pakakasama, 2007 (80)	108/317	94	14	0	272	42	3	0.342
Shi, 2011 (86)	307/558	236	66	5	445	109	4	0.335
Yalcin, 2014 (94)	156/180	82	64	10	112	58	10	0.496
Zhu, 2005 (98)	63/66	49	14		54	12		–
The first author, publication year	Case/control	*XRCC3 Thr241Met (rs861539)*						*p*-value of HWE
		Case			Control			
		Thr/Thr	Thr/Met	Met/Met	Thr/Thr	Thr/Met	Met/Met	
Abramenko, 2012 (47)	159/73	74	60	25	30	33	10	0.967
Banescu, 2013 (50)	78/121	36	30	12	85	27	9	**0.004**
Banescu, 2014 (51)	156/180	64	70	22	85	79	16	0.697
Bhatla, 2008 (54)	405/646	160	190	55	253	309	84	0.493
Erčulj, 2012 (61)	20/39	6	8	6	15	19	5	0.789
Fekry, 2018 (62)	48/48	42	4	2	41	5	2	**0.007**
Goričar, 2015 (64)	111/184	89	32		117	67		–
Hamdy, 2011 (65)	50/50	22	20	8	18	9	3	0.273
Liu, 2011 (71)	105/704	55	39	11	627	73	4	0.245
Matullo, 2006 (72)	169/1094	61	90	18	383	544	167	0.249
Miao, 2015 (75)	518/1033	470	45	3	902	130	1	0.094
Mutlu, 2015 (77)	25/30	9	12	4	13	11	6	0.219
Pei, 2018 (81)	266/266	214	39	13	241	19	6	**< 0.001**
Seedhouse, 2002 (84)	154/175	65	65	24	92	64	19	0.129
Seedhouse, 2004 (85)	260/175	119	103	38	92	64	19	0.129
Smolkova, 2014 (87)	459/549	178	216	65	216	256	77	0.934
Sorour, 2013 (88)	90/60	24	63	3	12	42	6	**0.001**
Wilson, 2023 (93)	96/103	53	40	3	58	27	18	**< 0.001**
Yalcin, 2014 (94)	156/180	64	70	22	85	79	16	0.697
Yang, 2011 (95)	379/704	311	57	11	627	73	4	0.245
Zhang, 2009 (97)	148/458	133	13	2	403	46	9	**< 0.001**

HWE, Hardy-Weinberg equilibrium. Bold number donates statistical significance (*p* < 0.05).

### 3.4 Pooled analysis

The forest plots are presented in **Supplementary Data Sheet 1**, while **Table 5** provides a summary of the pooled analysis results for eleven polymorphisms across various genetic models. For *ERCC1 C118T*, no significant associations were observed in the allelic, homozygous, heterozygous, dominant, or recessive models (p-values > 0.05), with low heterogeneity (I² = 0%). In contrast, *ERCC1 8092C>A* showed a significant association in the homozygous model (OR = 0.69, p = 0.01) with low heterogeneity (I² = 6%) and in the dominant model (OR = 0.78, p = 0.03) with no heterogeneity (I² = 0%). Other models for this polymorphism showed no significant associations, with varying heterogeneity. For *XPD/ERCC2 Lys751Gln*, significant associations were found across all genetic models (p-values < 0.05), with moderate heterogeneity (I² ranging from 42% to 63%). In contrast, *XPD/ERCC2 Asp312Asn* showed no significant associations in any model (p-values > 0.05), with no heterogeneity (I² = 0%).

**Table 5:** Summary of the results of pooled analysis of each genetic model for each polymorphism.

Polymorphism	Genetic model	Number of studies	OR	95%CI		*p*-value	I^2^
				Min.	Max.		
*ERCC1* C118T	Allelic	2	0.97	0.80	1.16	0.72	0%
	Homozygous	2	0.94	0.64	1.38	0.75	0%
	Heterozygous	2	1.11	0.79	1.56	0.55	0%
	Dominant	2	1.04	0.76	1.44	0.79	0%
	Recessive	2	0.89	0.67	1.19	0.43	0%
*ERCC1 8092C>A*	Allelic	2	1.00	0.64	1.57	1.00	84%
	Homozygous	2	0.69	0.52	0.93	**0.01**	6%
	Heterozygous	2	0.80	0.63	1.02	0.07	0%
	Dominant	2	0.78	0.62	0.97	**0.03**	0%
	Recessive	2	1.06	0.51	2.23	0.88	89%
*XPD/ERCC2 Lys751Gln*	Allelic	20	1.24	1.10	1.40	**0.0006**	63%
	Homozygous	20	1.45	1.12	1.87	**0.005**	51%
	Heterozygous	20	1.30	1.1	1.43	**< 0.0001**	42%
	Dominant	20	1.33	1.15	1.53	**0.0001**	52%
	Recessive	20	1.18	1.02	1.36	**0.02**	44%
*XPD/ERCC2 Asp312Asn*	Allelic	4	0.89	0.74	1.06	0.19	0%
	Homozygous	4	0.77	0.51	1.17	0.23	0%
	Heterozygous	4	0.91	0.71	1.16	0.44	0%
	Dominant	4	0.88	0.69	1.11	0.28	0%
	Recessive	4	0.83	0.56	1.22	0.33	0%
*XPC* Lys939Gln	Allelic	4	1.15	1.02	1.29	**0.02**	43%
	Homozygous	4	1.39	1.09	1.78	**0.007**	36%
	Heterozygous	4	1.07	0.90	1.28	0.44	2%
	Dominant	4	1.14	0.96	1.35	0.12	29%
	Recessive	4	1.32	1.06	1.64	**0.01**	26%
*XPF/ERCC4* Arg415Gln	Allelic	2	0.87	0.66	1.15	0.34	0%
	Homozygous	2	1.23	0.63	2.41	0.54	0%
	Heterozygous	2	0.65	0.45	0.94	**0.02**	0%
	Dominant	2	0.73	0.52	1.03	0.07	0%
	Recessive	2	1.51	0.79	2.88	0.21	0%
*XPG* 3507G > C	Allelic	2	0.75	0.40	1.44	0.39	82%
	Homozygous	2	0.90	0.51	1.59	0.72	0%
	Heterozygous	2	0.68	0.28	1.65	0.39	84%
	Dominant	2	0.69	0.30	1.60	0.39	84%
	Recessive	2	0.94	0.54	1.63	0.83	0%
*XRCC1 Arg194Trp (rs1799782)*	Allelic	24	1.33	1.07	1.65	**0.01**	83%
	Homozygous	24	1.52	0.94	2.47	0.09	73%
	Heterozygous	24	1.11	0.91	1.35	0.29	59%
	Dominant	26	1.27	1.02	1.57	**0.03**	73%
	Recessive	24	1.49	1.03	2.16	**0.04**	65%
*XRCC1 Arg399Gln (rs25487)*	Allelic	29	1.17	1.05	1.30	**0.004**	61%
	Homozygous	29	1.27	1.12	1.46	**0.0003**	45%
	Heterozygous	29	1.18	1.00	1.40	0.05	66%
	Dominant	31	1.22	1.04	1.42	**0.01**	68%
	Recessive	29	1.20	1.06	1.35	**0.005**	36%
*XRCC1 Arg280His (rs25489)*	Allelic	7	1.13	0.95	1.35	0.16	24%
	Homozygous	7	1.38	0.75	2.54	0.30	0%
	Heterozygous	7	1.14	0.93	1.39	0.22	9%
	Dominant	8	1.15	0.95	1.40	0.15	9%
	Recessive	7	1.27	0.69	2.31	0.44	0%
*XRCC3 Thr241Met (rs861539)*	Allelic	20	1.29	1.04	1.60	**0.02**	87%
	Homozygous	20	1.54	1.06	2.24	**0.02**	72%
	Heterozygous	20	1.30	1.03	1.64	**0.03**	76%
	Dominant	21	1.29	1.02	1.64	**0.04**	82%
	Recessive	20	1.41	1.01	1.96	**0.04**	68%

Bold number donates statistical significance (*p* < 0.05).

For *XPC Lys939Gln*, significant associations were observed in the allelic, homozygous, and recessive models (p-values < 0.05), with low to moderate heterogeneity (I² ranging from 26% to 43%), while other models showed no significant associations, with low heterogeneity. *XPF/ERCC4 Arg415Gln* exhibited a significant association in the heterozygous model (OR = 0.65, p = 0.02) with no heterogeneity (I² = 0%), whereas other models showed no significant associations, with no heterogeneity. Lastly, *XPG 3507G>C* showed no significant associations in any model (p-values > 0.05), though some models exhibited high heterogeneity (I² up to 84%).

The meta-analysis reveals that *XRCC1 Arg194Trp, XRCC1 Arg399Gln*, and *XRCC3 Thr241Met* polymorphisms are significantly associated with disease risk, particularly in allelic, dominant, and recessive models. The *XRCC3 Thr241Met* variant shows the strongest association (OR = 1.54, p = 0.02 in the homozygous model), while *XRCC1 Arg399Gln* also exhibits consistent significance across multiple models. In contrast, *XRCC1 Arg280His* does not show any significant association, indicating it may not contribute to disease susceptibility.

Notably, the high heterogeneity (I² > 60%) in many models suggests variability across studies, likely due to differences in populations, methodologies, or sample sizes. Despite this, the findings support the potential role of *XRCC1* and *XRCC3* genetic variations in leukemia susceptibility. Heterogeneity was further explored using subgroup analyses based on ethnicity, leukemia subtype, control source, age group, and sample size; however, substantial residual heterogeneity remained in several models.

### 3.5 Subgroup analysis

**Table 6** shows the results of subgroup analyses of the association of *ERCC2 Lys751Gln*, *ERCC2 Asp312Asn, XPC Lys939Gln*, *XRCC1 Arg194Trp, XRCC1 Arg399Gln*, *XRCC1 Arg280His*, and *XRCC3 Thr241Met* polymorphisms with the risk of leukemia. The results reported that the ethnicity, the sample size, the control source, and the subtype of leukemia were effective factors for *ERCC2 Lys751Gln* and *XPC Lys939Gln* polymorphisms and none of them were not effective factors for *ERCC2 Asp312Asn* polymorphism. We didn’t analyze polymorphisms with two and four studies, because the numbers of studies were not sufficient.

**Table 6:** Subgroup analysis of the association of polymorphisms with the risk of leukemia.

Polymorphism (N)	Subgroup (N)	Variables	Allelic	Homozygous	Heterozygous	Dominant	Recessive
*ERCC2 Lys751Gln* (20)	Ethnicity						
	Asian (3)	OR (95%CI)	1.28 (1.04, 1.59)	2.05 (078, 5.40)	1.25 (1.00, 1.58)	1.28 (1.02, 1.60)	1.97 (0.75, 5.19)
		*p*-value	**0.02**	0.15	0.05	**0.03**	0.17
		I^2^	0%	0%	3%	8%	0%
	Caucasian (12)	OR (95%CI)	1.15 (0.99, 1.34)	1.19 (0.99, 1.43)	1.29 (1.13, 1.46)	1.27 (1.05, 1.55)	1.04 (0.88, 1.23)
		*p*-value	0.07	0.06	**0.0001**	**0.01**	0.65
		I^2^	57%	48%	48%	52%	38%
	Mixed (5)	OR (95%CI)	1.39 (1.03, 1.88)	1.90 (1.08, 3.36)	1.38 (1.03, 1.85)	1.47 (1.04, 2.08)	1.63 (1.02, 2.59)
		*p*-value	**0.03**	**0.03**	**0.03**	**0.03**	**0.04**
		I^2^	77%	63%	53%	69%	51%
	Sample size						
	≥ 400 (7)	OR (95%CI)	1.25 (1.01, 1.55)	1.41 (0.91, 2.19)	1.29 (1.06, 1.58)	1.32 (1.04, 1.67)	1.25 (0.87, 1.79)
		*p*-value	**0.04**	0.12	**0.01**	**0.02**	0.23
		I^2^	82%	74%	62%	75%	66%
	< 400 (13)	OR (95%CI)	1.23 (1.10, 1.38)	1.47 (1.14, 1.90)	1.36 (1.16, 1.60)	1.36 (1.17, 1.59)	1.22 (0.96, 1.54)
		*p*-value	0.0003	0.003	0.0002	< 0.0001	0.11
		I^2^	24%	19%	29%	22%	24%
	Control source						
	HB (7)	OR (95%CI)	1.35 (1.08, 1.67)	1.74 (1.17, 2.58)	1.33 (1.05, 1.70)	1.42 (1.09, 1.85)	1.40 (1.14, 1.72)
		*p*-value	**0.007**	**0.006**	**0.02**	**0.01**	**0.002**
		I^2^	71%	55%	52%	64%	43%
	PB (13)	OR (95%CI)	1.17 (1.01, 1.35)	1.17 (0.95, 1.45)	1.29 (1.14, 1.47)	1.27 (1.12, 1.43)	1.01 (0.83, 1.23)
		*p*-value	**0.04**	0.14	**< 0.0001**	**0.0001**	0.92
		I^2^	55%	48%	42%	46%	40%
	Type of leukemia						
	AML (9)	OR (95%CI)	1.31 (1.07, 1.60)	1.61 (1.06, 2.45)	1.44 (1.13, 1.83)	1.44 (1.13, 1.84)	1.33 (0.91, 1.96)
		*p*-value	**0.009**	**0.03**	**0.003**	**0.003**	0.14
		I^2^	72%	58%	64%	68%	55%
	ALL (5)	OR (95%CI)	1.08 (0.89, 1.30)	1.14 (0.72, 1.82)	1.08 (0.83, 1.40)	1.10 (0.86, 1.41)	1.11 (0.72, 1.72)
		*p*-value	0.44	0.57	0.56	0.46	0.63
		I^2^	0%	0%	0%	0%	0%
	CML (3)	OR (95%CI)	1.47 (1.17, 1.84)	2.31 (1.44, 3.69)	1.47 (1.06, 2.03)	1.54 (1.15, 2.07)	1.87 (1.20, 2.91)
		*p*-value	**0.0008**	**0.0005**	**0.02**	**0.004**	**0.006**
		I^2^	1%	0%	0%	0%	0%
	CLL (2)	OR (95%CI)	1.20 (1.02, 1.42)	1.29 (0.90, 1.85)	1.44 (1.12, 1.85)	1.40 (1.10, 1.78)	1.06 (0.76, 1.47)
		*p*-value	**0.03**	0.17	**0.005**	**0.006**	0.75
		I^2^	0%	0%	0%	0%	0%
	Age group						
	Child (5)	OR (95%CI)	1.15 (0.98, 1.34)	1.38 (0.98, 1.94)	1.08 (0.87, 1.34)	1.14 (0.93, 1.40)	1.33 (0.97, 1.82)
		*p*-value	0.08	0.07	0.48	0.21	0.08
		I^2^	0%	0%	0%	0%	0%
	Adult (13)	OR (95%CI)	1.22 (1.00, 1.50)	1.45 (1.04, 2.01)	1.45 (1.20, 1.76)	1.44 (1.17, 1.77)	1.23 (0.91, 1.65)
		*p*-value	0.05	**0.03**	**0.0001**	**0.0005**	0.18
		I^2^	74%	63%	54%	66%	58%
*ERCC2 Asp312Asn* (4)	Ethnicity						
	Asian (1)	OR (95%CI)	0.67 (0.34, 1.32)	0.56 (0.03, 11.75)	0.72 (0.36, 1.45)	0.69 (0.34, 1.38)	0.58 (0.03, 12.21)
		*p*-value	0.25	0.71	0.35	0.29	0.73
		I^2^	–	–	–	–	–
	Caucasian (2)	OR (95%CI)	0.89 (0.72, 1.10)	0.81 (0.52, 1.26)	0.88 (0.64, 1.20)	0.86 (0.64, 1.16)	0.87 (0.58, 1.31)
		*p*-value	0.30	0.35	0.42	0.33	0.51
		I^2^	0%	0%	0%	0%	0%
	Mixed (1)	OR (95%CI)	0.96 (0.65, 1.44)	0.60 (0.18, 2.05)	1.12 (0.67, 1.85)	1.05 (0.64, 1.71)	0.57 (0.17, 1.92)
		*p*-value	0.86	0.42	0.67	0.85	0.37
		I^2^	–	–	–	–	–
	Sample size						
	≥ 400 (2)	OR (95%CI)	0.85 (0.68, 1.06)	0.75 (0.46, 1.25)	0.85 (0.62, 1.15)	0.82 (0.61, 1.10)	0.81 (0.50, 1.29)
		*p*-value	0.14	0.27	0.29	0.18	0.37
		I^2^	0%	0%	0%	0%	0%
	< 400 (2)	OR (95%CI)	0.97 (0.71, 1.31)	0.82 (0.39, 1.72)	1.03 (0.68, 1.56)	0.99 (0.67, 1.48)	0.87 (0.44, 1.73)
		*p*-value	0.84	0.60	0.90	0.97	0.69
		I^2^	0%	0%	0%	0%	0%
	Control source						
	HB (1)	OR (95%CI)	0.96 (0.65, 1.44)	0.60 (0.18, 2.05)	1.12 (0.67, 1.85)	1.05 (0.64, 1.71)	0.57 (0.17, 1.92)
		*p*-value	0.86	0.42	0.67	0.85	0.37
		I^2^	–	–	–	–	–
	PB (3)	OR (95%CI)	0.87 (0.71, 1.06)	0.80 (0.52, 1.25)	0.85 (0.64, 1.13)	0.83 (0.63, 1.09)	0.86 (0.57, 1.30)
		*p*-value	0.17	0.33	0.26	0.18	0.48
		I^2^	0%	0%	0%	0%	0%
	Type of leukemia						
	ALL (3)	OR (95%CI)	0.91 (0.69, 1.19)	0.80 (0.39, 1.64)	0.93 (0.65, 1.33)	0.90 (0.64, 1.27)	0.85 (0.44, 1.66)
		*p*-value	0.48	0.54	0.69	0.55	0.64
		I^2^	0%	0%	0%	0%	0%
	Age group						
	Child (3)	OR (95%CI)	0.91 (0.69, 1.19)	0.80 (0.39, 1.64)	0.93 (0.65, 1.33)	0.90 (0.64, 1.27)	0.85 (0.44, 1.66)
		*p*-value	0.48	0.54	0.69	0.55	0.64
		I^2^	0%	0%	0%	0%	0%
	Adult (1)	OR (95%CI)	0.87 (0.69, 1.11)	0.76 (0.46, 1.27)	0.88 (0.62, 1.25)	0.85 (0.61, 1.19)	0.81 (0.50, 1.31)
		*p*-value	0.27	0.30	0.49	0.35	0.40
		I^2^	–	–	–	–	–
*XPC* Lys939Gln (4)	Ethnicity						
	Asian (2)	OR (95%CI)	1.09 (0.95, 1.26)	1.27 (0.95, 1.70)	0.98 (0.80, 1.21)	1.04 (0.86, 1.26)	1.29 (0.99, 1.68)
		*p*-value	0.20	0.10	0.86	0.66	0.06
		I^2^	0%	0%	0%	0%	0%
	Caucasian (1)	OR (95%CI)	1.02 (0.72, 1.44)	1.05 (0.50, 2.21)	1.23 (0.66, 2.30)	1.17 (0.64, 2.15)	0.91 (0.51, 1.62)
		*p*-value	0.91	0.89	0.52	0.60	0.75
		I^2^	–	–	–	–	–
	Mixed (1)	OR (95%CI)	1.53 (1.16, 2.03)	2.48 (1.35, 4.56)	1.48 (0.96, 2.27)	1.67 (1.11, 2.51)	1.98 (1.14, 3.43)
		*p*-value	**0.003**	**0.003**	0.07	**0.01**	**0.02**
		I^2^	–	–	–	–	–
	Sample size						
	≥ 400 (3)	OR (95%CI)	1.19 (0.96, 1.48)	1.50 (0.97, 2.31)	1.06 (0.88, 1.27)	1.18 (0.89, 1.55)	1.41 (1.11, 1.78)
		*p*-value	0.10	0.07	0.54	0.24	**0.005**
		I^2^	57%	51%	30%	52%	11%
	< 400 (1)	OR (95%CI)	1.02 (0.72, 1.44)	1.05 (0.50, 2.21)	1.23 (0.66, 2.30)	1.17 (0.64, 2.15)	0.91 (0.51, 1.62)
		*p*-value	0.91	0.89	0.52	0.60	0.75
		I^2^	–	–	–	–	–
	Control source						
	PB (4)	OR (95%CI)	1.15 (0.02, 1.29)	1.39 (1.09, 1.78)	1.07 (0.90, 1.28)	1.14 (0.96, 1.35)	1.32 (1.06, 1.64)
		*p*-value	**0.02**	**0.007**	0.44	0.12	**0.01**
		I^2^	43%	36%	2%	29%	26%
	Type of leukemia						
	AML (2)	OR (95%CI)	1.10 (0.95, 1.26)	1.27 (0.95, 1.70)	1.00 (0.80, 1.25)	1.07 (0.87, 1.31)	1.24 (0.96, 1.60)
		*p*-value	0.20	0.10	0.98	0.55	0.11
		I^2^	0%	0%	0%	0%	28%
	ALL (1)	OR (95%CI)	1.53 (1.16, 2.03)	2.48 (1.35, 4.56)	1.48 (0.96, 2.27)	1.67 (1.11, 2.51)	1.98 (1.14, 3.43)
		*p*-value	**0.003**	**0.003**	0.07	**0.01**	**0.02**
		I^2^	–	–	–	–	–
	CML (1)	OR (95%CI)	1.02 (0.76, 1.38)	1.07 (0.53, 2.14)	1.01 (0.67, 1.51)	1.02 (0.70, 1.49)	1.06 (0.54, 2.08)
		*p*-value	0.88	0.86	0.97	0.92	0.86
		I^2^	–	–	–	–	–
	Age group						
	Adult (2)	OR (95%CI)	1.02 (0.82, 1.28)	1.06 (0.64, 1.76)	1.07 (0.76, 1.50)	1.06 (0.77, 1.46)	0.97 (0.63, 1.50)
		*p*-value	0.85	0.82	0.70	0.72	0.89
		I^2^	0%	0%	0%	0%	0%
*XRCC1 Arg194Trp (rs1799782)*	Ethnicity						
	Asian (7)*	OR (95%CI)	1.05 (0.86, 1.29)	0.97 (0.60, 1.58)	0.97 (0.73, 1.28)	1.01 (0.78, 1.30)	1.19 (0.98, 1.44)
		*p*-value	0.64	0.92	0.81	0.95	0.08
		I^2^	70%	57%	67%	65%	47%
	Caucasian (15)*	OR (95%CI)	1.59 (1.05, 2.40)	2.16 (0.97, 4.79)	1.33 (0.94, 1.87)	1.59 (1.09, 2.33)	1.84 (0.96, 3.54)
		*p*-value	**0.03**	0.06	0.11	**0.02**	0.07
		I^2^	86%	71%	63%	77%	62%
	Mixed (2)	OR (95%CI)	1.06 (0.78, 1.46)	1.28 (0.43, 3.83)	1.04 (0.73, 1.50)	1.06 (0.74, 1.50)	1.25 (0.42, 3.74)
		*p*-value	0.70	0.66	0.82	0.76	0.69
		I^2^	19%	29%	0%	0%	24%
	Sample size						
	≥ 400 (7)*	OR (95%CI)	1.17 (1.06, 1.29)	1.24 (0.96, 1.60)	1.08 (0.87, 1.34)	1.04 (0.85, 1.28)	1.28 (1.05, 1.56)
		*p*-value	**0.002**	0.11	0.50	0.68	**0.01**
		I^2^	0%	0%	52%	54%	0%
	< 400 (17)*	OR (95%CI)	1.45 (0.97, 2.17)	1.65 (0.75, 3.64)	1.22 (0.89, 1.68)	1.52 (1.06, 2.20)	1.45 (0.75, 2.80)
		*p*-value	0.07	0.21	0.21	**0.02**	0.27
		I^2^	88%	78%	64%	77%	72%
	Control source						
	PB (18)**	OR (95%CI)	1.30 (1.04, 1.64)	1.55 (0.93, 2.59)	1.11 (0.99, 1.24)	1.22 (0.99, 1.50)	1.64 (1.07, 2.51)
		*p*-value	**0.02**	0.09	0.08	0.06	**0.02**
		I^2^	83%	70%	33%	68%	68%
	HB (6)	OR (95%CI)	1.48 (0.73, 3.00)	1.55 (0.34, 7.15)	2.30 (0.91, 5.82)	1.87 (0.80, 4.37)	1.07 (0.46, 2.51)
		*p*-value	0.27	0.57	0.08	0.15	0.87
		I^2^	88%	83%	86%	85%	59%
	Type of leukemia						
	AML (4)	OR (95%CI)	1.21 (1.08, 1.37)	1.43 (1.07, 1.92)	1.26 (1.07, 1.48)	1.29 (1.10, 1.51)	1.55 (0.69, 3.49)
		*p*-value	**0.002**	**0.02**	**0.006**	**0.002**	0.29
		I^2^	0%	40%	0%	0%	53%
	ALL (10)*	OR (95%CI)	1.07 (0.83, 1.39)	0.93 (0.62, 1.39)	1.02 (0.84, 1.24)	1.01 (0.85, 1.21)	0.98 (0.68, 1.45)
		*p*-value	0.59	0.73	0.84	0.87	0.92
		I^2^	54%	36%	37%	38%	30%
	CML (5)	OR (95%CI)	1.91 (0.88, 4.13)	2.10 (0.59, 7.43)	0.97 (0.51, 1.83)	1.69 (0.67, 4.22)	2.31 (0.90, 5.96)
		*p*-value	0.10	0.25	0.92	0.26	0.08
		I^2^	94%	86%	59%	90%	86%
	CLL (2)	OR (95%CI)	1.26 (0.90, 1.76)	1.15 (0.36, 3.63)	1.03 (0.42, 2.53)	1.10 (0.53, 2.29)	1.11 (0.35, 3.52)
		*p*-value	0.19	0.82	0.94	0.80	0.85
		I^2^	27%	0%	64%	53%	0%
	Age group						
	Child (11)*	OR (95%CI)	1.01 (0.79, 1.29)	0.82 (0.7, 1.18)	1.03 (0.81, 1.31)	1.04 (0.83, 1.30)	0.87 (0.61, 1.24)
		*p*-value	0.91	0.28	0.82	0.73	0.44
		I^2^	56%	41%	35%	40%	34%
	Adult (12)*	OR (95%CI)	1.62 (1.10, 2.38)	2.39 (1.09, 5.26)	1.15 (0.81, 1.63)	1.49 (0.99, 2.26)	2.19 (1.25, 3.83)
		*p*-value	**0.01**	**0.03**	0.43	0.06	**0.006**
		I^2^	88%	75%	69%	82%	68%
*XRCC1 Arg399Gln (rs25487)*	Ethnicity						
	Asian (6)*	OR (95%CI)	1.26 (1.01, 1.56)	1.39 (1.08, 1.78)	1.17 (0.83, 1.66)	1.36 (0.97, 1.91)	1.49 (1.18, 1.87)
		*p*-value	**0.04**	**0.01**	0.37	0.07	**0.0007**
		I^2^	74%	30%	80%	81%	0%
	Caucasian (19)*	OR (95%CI)	1.13 (0.97, 1.31)	1.21 (0.91, 1.61)	1.14 (1.00, 1.29)	1.15 (0.96, 1.37)	1.10 (0.94, 1.29)
		*p*-value	0.12	0.19	**0.04**	0.14	0.23
		I^2^	63%	56%	25%	54%	33%
	Mixed (4)	OR (95%CI)	1.18 (1.00, 1.39)	1.38 (0.91, 2.09)	1.21 (0.56, 2.59)	1.27 (0.70, 2.30)	1.20 (0.53, 2.70)
		*p*-value	0.05	0.12	0.63	0.44	0.67
		I^2^	32%	3%	90%	85%	67%
	Sample size						
	≥ 400 (8)	OR (95%CI)	1.07 (0.99, 1.16)	1.21 (1.00, 1.45)	1.06 (0.81, 1.40)	1.09 (0.87, 1.36)	1.22 (1.03, 1.45)
		*p*-value	0.09	0.05	0.66	0.47	**0.02**
		I^2^	15%	0%	82%	76%	37%
	< 400 (21)**	OR (95%CI)	1.22 (1.03, 1.44)	1.41 (1.01, 1.95)	1.27 (1.11, 1.46)	1.29 (1.05, 1.59)	1.17 (0.98, 1.40)
		*p*-value	**0.02**	**0.04**	**0.0006**	**0.02**	0.09
		I^2^	67%	59%	45%	63%	38%
	Control source						
	PB (21)**	OR (95%CI)	1.15 (1.02, 1.30)	1.29 (1.02, 1.62	1.12 (0.96, 1.31)	1.18 (1.00, 1.39)	1.21 (1.06, 1.39)
		*p*-value	**0.03**	**0.03**	0.15	0.05	**0.005**
		I^2^	65%	51%	52%	65%	27%
	HB (8)	OR (95%CI)	1.21 (1.06, 1.39)	1.40 (1.02, 1.92)	1.27 (0.79, 2.07)	1.31 (0.88, 1.95)	1.16 (0.71, 1.91)
		*p*-value	**0.006**	**0.04**	0.33	0.19	0.55
		I^2^	50%	31%	81%	75%	56%
	Type of leukemia						
	AML (8)	OR (95%CI)	1.00 (0.81, 1.23)	0.93 (0.72, 1.21)	1.12 (0.75, 1.68)	1.08 (0.76, 1.53)	0.86 (0.55, 1.33)
		*p*-value	0.98	0.60	0.59	0.66	0.49
		I^2^	65%	49%	83%	79%	55%
	ALL (9)*	OR (95%CI)	1.22 (1.07, 1.39)	1.45 (1.09, 1.95)	1.24 (0.93, 1.65)	1.17 (0.88, 1.56)	1.31 (1.00, 1.73)
		*p*-value	**0.002**	**0.01**	0.15	0.27	0.05
		I^2^	48%	0%	54%	64%	0%
	CML (5)	OR (95%CI)	1.29 (0.96, 1.74)	1.47 (0.94, 2.30)	1.02 (0.69, 1.50)	1.20 (0.80, 1.80)	1.48 (1.15, 1.91)
		*p*-value	0.10	0.09	0.92	0.39	**0.002**
		I^2^	74%	54%	51%	70%	14%
	CLL (2)	OR (95%CI)	1.30 (0.71, 2.37)	1.83 (0.43, 7.84)	2.24 (0.80, 6.24)	2.03 (0.68, 6.08)	1.00 (0.60, 1.67)
		*p*-value	0.40	0.42	0.12	0.21	0.99
		I^2^	74%	80%	72%	79%	51%
	Age group						
	Child (10)**	OR (95%CI)	1.26 (1.05, 1.51)	1.56 (1.18, 2.06)	1.26 (1.06, 1.50)	1.29 (0.98, 1.69)	1.39 (1.07, 1.81)
		*p*-value	**0.01**	**0.002**	**0.009**	0.07	**0.01**
		I^2^	0%	3%	49%	65%	0%
	Adult (16)	OR (95%CI)	1.16 (0.99, 1.35)	1.26 (0.93, 1.71)	1.18 (0.91, 1.52)	1.21 (0.96, 1.53)	1.12 (0.86, 1.46)
		*p*-value	0.06	0.13	0.21	0.10	0.39
		I^2^	68%	61%	74%	73%	56%
*XRCC1 Arg280His (rs25489)*	Ethnicity						
	Asian (3)*	OR (95%CI)	1.00 (0.66, 1.50)	1.37 (0.55, 3.37)	1.08 (0.84, 1.40)	1.11 (0.87, 1.41)	1.09 (0.85, 1.41)
		*p*-value	0.98	0.50	0.54	0.42	0.50
		I^2^	56%	0%	35%	32%	48%
	Caucasian (2)	OR (95%CI)	1.25 (0.91, 1.72)	1.32 (0.55, 3.17)	1.34 (0.90, 2.01)	1.33 (0.91, 1.96)	1.33 (0.91, 1.96)
		*p*-value	0.17	0.53	0.15	0.14	0.14
		I^2^	0%	0%	5%	5%	5%
	Sample size						
	≥ 400 (3)	OR (95%CI)	0.82 (0.44, 1.52)	1.27 (0.45, 3.63)	1.01 (0.75, 1.35)	0.86 (0.49, 1.52)	0.86 (0.49, 1.52)
		*p*-value	0.53	0.65	0.95	0.61	0.61
		I^2^	66%	4%	45%	58%	58%
	< 400 (4)*	OR (95%CI)	1.23 (0.97, 1.57)	1.44 (0.68, 3.04)	1.27 (0.96, 1.69)	1.28 (0.99, 1.67)	1.28 (0.97, 1.69)
		*p*-value	0.08	0.34	0.10	0.06	0.08
		I^2^	0%	0%	0%	0%	0%
	Control source						
	PB (1)	OR (95%CI)	1.36 (0.83, 2.22)	1.68 (0.27, 10.31)	1.42 (0.80, 2.50)	1.43 (0.82, 2.50)	1.43 (0.82, 2.50)
		*p*-value	0.22	0.58	0.23	0.20	0.20
		I^2^	–	–	–	–	–
	HB (6)*	OR (95%CI)	1.10 (0.91, 1.33)	1.35 (0.71, 2.57)	1.10 (0.88, 1.37)	1.11 (0.87, 1.40)	1.11 (0.90, 1.37)
		*p*-value	0.31	0.36	0.39	0.40	0.34
		I^2^	32%	0%	17%	15%	28%
	Age group						
	Child (3)*	OR (95%CI)	1.11 (0.82, 1.50)	1.26 (0.37, 4.24)	1.13 (0.80, 1.59)	1.15 (0.84, 1.57)	1.20 (0.36, 4.05)
		*p*-value	0.51	0.71	0.49	0.39	0.77
		I^2^	0%	0%	0%	0%	0%
	Adult (4)	OR (95%CI)	1.05 (0.72, 1.54)	1.43 (0.71, 2.87)	1.14 (0.89, 1.47)	1.06 (0.70, 1.62)	1.29 (0.64, 2.58)
		*p*-value	0.79	0.32	0.31	0.77	0.47
		I^2^	53%	0%	47%	53%	0%
*XRCC3 Thr241Met (rs861539)*	Ethnicity						
	Asian (5)	OR (95%CI)	1.79 (0.80, 3.79)	4.49 (1.26, 15.99)	1.66 (0.75, 3.66)	1.78 (0.78, 4.10)	4.02 (1.30, 12.40)
		*p*-value	0.15	**0.02**	0.21	0.17	**0.02**
		I^2^	95%	78%	93%	95%	72%
	Caucasian (13)*	OR (95%CI)	1.17 (1.00, 1.37)	1.19 (0.97, 1.45)	1.18 (1.03, 1.35)	1.15 (1.01, 1.30)	1.10 (0.91, 1.33)
		*p*-value	**0.04**	0.09	**0.02**	**0.03**	0.31
		I^2^	54%	50%	7%	34%	48%
	Mixed (2)	OR (95%CI)	0.97 (0.82, 1.15)	0.62 (0.16, 2.37)	0.95 (0.73, 1.22)	0.95 (0.75, 1.21)	0.70 (0.23, 2.16)
		*p*-value	0.72	0.49	0.67	0.69	0.53
		I^2^	16%	67%	0%	0%	62%
	Sample size						
	≥ 400 (9)	OR (95%CI)	1.39 (0.96, 2.00)	1.95 (1.08, 3.53)	1.34 (0.93, 1.94)	1.40 (0.94, 2.09)	1.76 (1.04, 2.97)
		*p*-value	0.08	**0.03**	0.12	0.10	**0.04**
		I^2^	93%	83%	88%	91%	80%
	< 400 (11)*	OR (95%CI)	1.20 (0.97, 1.48)	1.30 (0.81, 2.07)	1.27 (1.04, 1.54)	1.21 (1.02, 1.43)	1.19 (0.77, 1.84)
		*p*-value	0.09	0.28	**0.02**	**0.03**	0.42
		I^2^	54%	53%	16%	41%	51%
	Control source						
	PB (17)*	OR (95%CI)	1.37 (1.08, 1.74)	1.73 (1.16, 2.58)	1.39 (1.09, 1.79)	1.38 (1.07, 1.79)	1.53 (1.06, 2.19)
		*p*-value	**0.010**	**0.007**	**0.009**	**0.01**	**0.02**
		I^2^	88%	75%	79%	84%	72%
	HB (3)	OR (95%CI)	0.85 (0.64, 1.15)	0.75 (0.38, 1.48)	0.75 (0.47, 1.17)	0.77 (0.50, 1.18)	0.86 (0.46, 1.63)
		*p*-value	0.29	0.41	0.20	0.23	0.65
		I^2^	0%	19%	0%	0%	22%
	Type of leukemia						
	AML (8)	OR (95%CI)	1.47 (0.94, 2.30)	2.37 (1.10, 5.13)	1.40 (0.88, 2.22)	1.49 (0.90, 2.46)	2.08 (1.06, 4.09)
		*p*-value	0.09	**0.03**	0.16	0.12	**0.03**
		I^2^	93%	83%	89%	91%	79%
	ALL (2)*	OR (95%CI)	0.99 (0.84, 1.18)	1.00 (0.69, 1.45)	1.00 (0.78, 1.28)	0.93 (0.76, 1.15)	0.99 (0.70, 1.40)
		*p*-value	0.93	0.99	0.98	0.52	0.95
		I^2^	0%	0%	0%	0%	0%
	CML (2)	OR (95%CI)	1.29 (1.03, 1.62)	1.83 (1.10, 3.04)	1.18 (0.85, 1.63)	1.29 (0.95, 1.75)	1.68 (1.04, 2.73)
		*p*-value	**0.03**	**0.02**	0.32	0.11	**0.03**
		I^2^	0%	0%	0%	0%	0%
	CLL (2)	OR (95%CI)	0.96 (0.67, 1.37)	1.00 (0.48, 2.10)	0.86 (0.51, 1.47)	0.90 (0.55, 1.47)	1.06 (0.53, 2.09)
		*p*-value	0.82	1.00	0.59	0.66	0.87
		I^2^	0%	0%	21%	0%	0%
	Age group						
	Child (6)*	OR (95%CI)	1.10 (0.85, 1.42)	1.03 (0.62, 1.70)	1.10 (0.93, 1.30)	1.07 (0.83, 1.38)	1.01 (0.61, 1.67)
		*p*-value	0.48	0.92	0.25	0.59	0.98
		I^2^	70%	59%	48%	54%	64%
	Adult (11)	OR (95%CI)	1.40 (0.99, 1.98)	2.03 (1.14, 3.61)	1.34 (0.93, 1.92)	1.42 (0.96, 2.09)	1.81 (0.09, 3.00)
		*p*-value	0.05	**0.02**	0.11	0.08	**0.02**
		I^2^	91%	79%	85%	88%	76%

Bold number donates statistical significance (*p* < 0.05). OR, Odds ratio; CI, Confidence interval; CML, Chronic Myeloid Leukemia; AML, Acute Myeloid Leukemia; ALL, Acute Lymphoblastic Leukemia. * There is one more study in dominant model. ** There are two more studies in dominant model.

The *XRCC1 Arg194Trp* variant shows varying levels of association depending on ethnicity, with Caucasians exhibiting a significant increased risk in dominant (OR = 1.59, p = 0.02) and allelic (OR = 1.59, p = 0.03) models, while Asians and mixed populations show no significant associations. Sample size also plays a role, as studies with ≥400 participants report stronger associations (p = 0.002 in the allelic model) compared to smaller studies. For leukemia subtypes, AML shows the most consistent risk increase (p < 0.05 in most models), whereas ALL, CML, and CLL exhibit weaker or non-significant associations.

Similarly, for *XRCC1 Arg399Gln*, Asians demonstrate a significant risk increase in recessive (OR = 1.49, p = 0.0007) and homozygous (OR = 1.39, p = 0.01) models, whereas Caucasians show weaker associations (p > 0.05 in most models). Studies with smaller sample sizes (<400) report stronger associations, suggesting potential sample size effects. Among leukemia types, ALL exhibits a significant risk increase across multiple genetic models (p < 0.05), while AML and CML show weaker or inconsistent associations. The *XRCC1 Arg280His* variant appears to have minimal impact, with no significant findings across ethnicities or leukemia subtypes. Overall, these results suggest that *XRCC1* polymorphisms may contribute to leukemia susceptibility, with varying effects based on genetic model, ethnicity, and leukemia type.

The *XRCC3 Thr241Met* polymorphism is associated with an increased risk of leukemia, particularly in Asians, where the dominant and recessive models show significant associations (p = 0.02). In Caucasians, significant findings are observed in the homozygous, dominant, and allele models, while no association is found in mixed populations. Larger sample studies (≥400) and PB controls show stronger associations, while smaller studies and HB controls HB do not. Among leukemia subtypes, AML and CML exhibit significant associations, while ALL and CLL do not. High heterogeneity (I² > 70%) in several analyses suggests variability among studies, warranting cautious interpretation. Overall, this polymorphism appears to contribute to leukemia risk, particularly in Asians, larger studies, and specific subtypes (AML, CML).

Across the polymorphisms analyzed for association with leukemia risk, age-stratified results revealed distinct patterns: *ERCC2 Lys751Gln, XRCC1 Arg194Trp*, and *XRCC3 Thr241Met* showed significant associations predominantly in adults (with multiple genetic models reaching *p* < 0.05), whereas *ERCC2 Asp312Asn* and *XRCC1 Arg280His* did not show significant associations in either age group. In contrast, *XRCC1 Arg399Gln* was significantly associated with increased leukemia risk in children (not adults), and *XPC Lys939Gln* did not demonstrate a significant age-specific association, with available data in adults showing no significant effects. Overall, adult subjects tended to show more frequent significant genetic associations for certain DNA repair gene polymorphisms, while only *XRCC1 Arg399Gln* was significant in childhood leukemia.

### 3.6 Meta-regression analysis

**Table 7** presents the random-effects meta-regression analyses evaluating the influence of study-level characteristics—including publication year, sample size, and methodological quality score—on the association between *ERCC2 Lys751Gln, XRCC1 Arg194Trp, XRCC1 Arg399Gln, XRCC1 Arg280His*, and *XRCC3 Thr241Met* polymorphisms and leukemia risk. Polymorphisms represented by fewer than five studies were not included due to insufficient statistical power.

**Table 7:** Random-effect meta-regression analyses the association of *ERCC2 Lys751Gln* polymorphism with the risk of leukemia.

Polymorphism (N)	Variable	Model	Coefficient	95% lower	95% upper	Z-value	*p*-value
*ERCC2 Lys751Gln* (20)	Publication year	Allelic	< 0.0001	-0.0006	0.0006	0.03	0.9739
		Homozygous	0.0006	-0.0008	0.0019	0.82	0.4146
		Heterozygous	-0.0003	-0.0009	0.0004	-0.75	0.4529
		Dominant	-0.0001	-0.0009	0.0006	-0.41	0.6785
		Recessive	0.0008	-0.0004	0.0020	1.28	0.1995
	Sample size	Allelic	-0.0001	-0.0003	0.0001	-0.89	0.3719
		Homozygous	-0.0002	-0.0008	0.0003	-0.77	0.4403
		Heterozygous	-0.0002	-0.0004	0.0001	-1.32	0.1865
		Dominant	-0.0002	-0.0004	0.0001	-1.23	0.2183
		Recessive	-0.0001	-0.0006	0.0004	-0.43	0.6669
	Quality score	Allelic	0.0313	-0.1163	0.1789	0.42	0.6780
		Homozygous	-0.0743	-0.4038	0.2551	-0.44	0.6583
		Heterozygous	0.1111	-0.0517	0.2740	1.34	0.1810
		Dominant	0.0850	-0.0871	0.2571	0.97	0.3331
		Recessive	-0.1555	-0.4509	0.1399	-1.03	0.3021
*XRCC1 Arg194Trp (rs1799782)*	Publication year	Allelic	- 0.0026	- 0.0052	< 0.0001	- 1.93	0.0541
		Homozygous	- 0.0022	- 0.0047	0.0003	- 1.73	0.0836
		Heterozygous	- < 0.0001	- 0.0010	0.0009	- 0.10	0.9238
		Dominant	- 0.0005	- 0.0016	0.0005	- 0.96	0.3385
		Recessive	- 0.0022	- 0.0042	- 0.0003	- 2.23	**0.0257**
	Sample size	Allelic	- 0.0016	- 0.0027	- 0.0006	- 3.15	**0.0016**
		Homozygous	- < 0.0001	- 0.0008	0.0007	- 0.13	0.8993
		Heterozygous	< 0.0001	- 0.0003	0.0003	0.13	0.8945
		Dominant	- 0.0001	- 0.0005	0.0003	- 0.44	0.6615
		Recessive	< 0.0001	- 0.0006	0.0006	0.05	0.9581
	Quality score	Allelic	0.0063	- 0.6539	0.6665	0.02	0.9850
		Homozygous	0.6065	- 0.0023	1.2134	1.95	0.0509
		Heterozygous	0.0241	- 0.2126	0.2608	0.20	0.8418
		Dominant	0.1661	- 0.0957	0.4278	1.24	0.2137
		Recessive	0.6063	0.1293	1.0780	2.49	**0.0126**
*XRCC1 Arg399Gln (rs25487)*	Publication year	Allelic	- 0.0001	- 0.0007	0.0004	- 0.52	0.6020
		Homozygous	- 0.0003	- 0.0013	0.0007	- 0.54	0.5886
		Heterozygous	0.0001	- 0.0007	0.0010	0.31	0.7564
		Dominant	0.0005	- 0.0003	0.0013	1.31	0.1902
		Recessive	- 0.0003	- 0.0012	0.0005	- 0.73	0.4632
	Sample size	Allelic	- 0.0001	- 0.0003	0.0001	- 0.81	0.4204
		Homozygous	- < 0.0001	- 0.0005	0.0004	- 0.19	0.8523
		Heterozygous	- 0.0002	- 0.0006	0.0001	- 1.19	0.2332
		Dominant	- 0.0001	- 0.0005	0.0002	- 0.67	0.5014
		Recessive	0.0001	- 0.0002	0.0005	0.76	0.4496
	Quality score	Allelic	0.0599	- 0.0755	0.1953	0.87	0.3861
		Homozygous	0.1042	- 0.1525	0.3609	0.80	0.4263
		Heterozygous	0.0002	- 0.2142	0.2145	0.00	0.9988
		Dominant	- 0.1096	- 0.3130	0.0939	- 1.06	0.2913
		Recessive	0.0886	- 0.1226	0.2998	0.82	0.4108
*XRCC1 Arg280His (rs25489)*	Publication year	Allelic	- 0.0004	- 0.0025	0.0016	- 0.41	0.6849
		Homozygous	- 0.0009	- 0.0079	0.0061	- 0.25	0.7994
		Heterozygous	- 0.0004	- 0.0025	0.0016	- 0.41	0.6815
		Dominant	- 0.0005	- 0.0025	0.0014	- 0.52	0.6012
		Recessive	- 0.0018	- 0.0086	0.0051	- 0.50	0.6141
	Sample size	Allelic	- 0.0006	- 0.0024	0.0012	- 0.69	0.4920
		Homozygous	- 0.0001	- 0.0049	0.0048	- 0.02	0.9826
		Heterozygous	- 0.0007	- 0.0025	0.0011	- 0.80	0.4262
		Dominant	- 0.0008	- 0.0024	0.0009	- 0.89	0.3753
		Recessive	0.0001	- 0.0047	0.0049	0.05	0.9627
	Quality score	Allelic	0.1526	- 0.4409	0.7460	0.50	0.6143
		Homozygous	0.2703	- 1.6686	2.2092	0.27	0.7847
		Heterozygous	0.1629	- 0.4279	0.7537	0.54	0.5890
		Dominant	0.1884	- 0.3736	0.7504	0.66	0.5112
		Recessive	0.4714	- 1.4285	2.3713	0.49	0.6267
*XRCC3 Thr241Met (rs861539)*	Publication year	Allelic	0.0020	0.0011	0.0029	4.57	**< 0.0001**
		Homozygous	0.0032	0.0015	0.0049	3.77	**0.0002**
		Heterozygous	0.0019	0.0010	0.0028	4.03	**0.0001**
		Dominant	0.0021	0.0012	0.0030	4.68	**< 0.0001**
		Recessive	0.0028	0.0013	0.0044	3.52	**0.0004**
	Sample size	Allelic	0.0003	- 0.0001	0.0008	1.62	0.1051
		Homozygous	0.0011	0.0002	0.0020	2.34	**0.0193**
		Heterozygous	0.0002	- 0.0003	0.0006	0.67	0.5044
		Dominant	0.0003	- 0.0002	0.0007	1.17	0.2438
		Recessive	0.0010	0.0002	0.0018	2.33	**0.019**
	Quality score	Allelic	- 0.4963	- 0.7233	- 0.2693	- 4.29	**< 0.0001**
		Homozygous	- 0.8309	- 1.2775	- 0.3843	- 3.65	**0.0003**
		Heterozygous	- 0.4511	- 0.6931	- 0.2090	- 3.65	**0.0003**
		Dominant	- 0.5163	- 0.7475	- 0.2851	- 4.38	**< 0.0001**
		Recessive	- 0.7346	- 1.1532	- 0.3160	- 3.44	**0.0006**

Bold number donates statistical significance (*p* < 0.05).

For *ERCC2 Lys751Gln*, none of the examined covariates significantly influenced effect sizes, indicating that publication year, sample size, and study quality were not major sources of heterogeneity.

For *XRCC1 Arg194Trp*, publication year significantly influenced the recessive model (p = 0.0257), suggesting a temporal trend in reported effect sizes. Sample size significantly affected the allelic model (p = 0.0016), indicating that larger studies tended to report smaller effect estimates. Additionally, quality score significantly influenced the recessive model (p = 0.0126), suggesting that methodological rigor may affect observed associations.

In contrast, *XRCC1 Arg399Gln* and *XRCC1 Arg280His* showed no significant relationships with publication year, sample size, or quality score across genetic models, indicating relative stability of effect estimates across study characteristics.

For *XRCC3 Thr241Met*, publication year was significantly associated with effect sizes across all models (p < 0.0001 to p = 0.0004), suggesting consistent reporting trends over time. Sample size significantly influenced the homozygous (p = 0.0193) and recessive (p = 0.0190) models, indicating potential small-study effects. Notably, quality score demonstrated significant negative associations across models (p < 0.0001 to p = 0.0006), suggesting that studies with lower methodological quality reported larger effect sizes.

### 3.7 Sensitivity analysis

The “cumulative” and “one-study-removed” sensitivity analyses were conducted to assess the reliability of the pooled results for the polymorphism. Both approaches demonstrated that excluding individual studies or progressively accumulating data did not alter the overall effect estimates. This consistency suggests that the pooled results were stable and not unduly influenced by any single study.

Several studies showed deviation from HWE in control groups (**Tables 3**, **4**). To evaluate the potential influence of these deviations on pooled outcomes, sensitivity analyses were conducted excluding studies with HWE violations. The direction and magnitude of pooled effect estimates remained largely unchanged across genetic models, indicating that deviations from HWE did not materially affect the overall conclusions of the meta-analysis.

### 3.8 Trial sequential analysis

The TSA plots for the *ERCC2 Lys751Gln, ERCC2 Asp312Asn, XPC Lys939Gln, XRCC1 Arg194Trp, XRCC1 Arg399Gln, XRCC1 Arg280His*, and *XRCC3 Thr241Met* polymorphisms (each with more than three studies) are provided in **Supplementary Data Sheet 2**. The results indicate that the z-curve crossed the required information size (RIS) line in five genetic models of *ERCC2 Lys751Gln*, the homozygous and dominant models of *ERCC2 Asp312Asn*, the homozygous and recessive models of *XRCC1 Arg194Trp*, and the allelic, homozygous, dominant, and recessive models of *XRCC1 Arg399Gln*, demonstrated a sufficient level of evidence, and no further study. In contrast, the Z-curve did not reach the RIS boundary for other analyses, suggesting that the available evidence remains inconclusive and that additional well-designed studies are required to confirm these associations.

### 3.9 Publication bias

The funnel plots are provided in **Supplementary Data Sheet 3**. For the *ERCC2 Lys751Gln, ERCC2 Asp312Asn, XPC Lys939Gln*, and *XRCC3 Thr241Met* polymorphisms (each with more than three studies), the p-values for both Egger’s and Begg’s tests were greater than 0.10, indicating no evidence of publication bias.

However, publication bias was detected in specific models of certain polymorphisms. For *XRCC1 Arg194Trp*, both tests in the allelic model yielded p-values below 0.10. Similarly, for *XRCC1 Arg399Gln*, Begg’s test in the heterozygous model indicated bias. In the case of *XRCC1 Arg280His*, both tests in the allelic and dominant models and Begg’s test in the homozygous model had p-values below 0.10, suggesting publication bias for these polymorphisms.

**Table 8** shows the trim-and-fill analyses for *XRCC1 Arg194Trp* in allelic model*, XRCC1 Arg399Gln* in heterozygous model*, XRCC1 Arg280Hi* in allelic and dominant models. For *XRCC1 Arg194Trp* in the allelic model, no missing studies were imputed and the adjusted effect size remained unchanged, indicating that publication bias had minimal impact on the pooled estimate. Similarly, for *XRCC1 Arg280His* in both allelic and dominant models, trim-and-fill analysis did not identify any missing studies and the pooled estimates were stable after adjustment. In contrast, for *XRCC1 Arg399Gln* in the heterozygous model, seven studies were imputed and the adjusted pooled estimates were attenuated, with confidence intervals crossing unity, suggesting that the initial association may have been influenced by publication bias and should be interpreted with caution.

**Table 8:** Trim-and-fill analyses for *XRCC1* polymorphisms.

Polymorphism	Genetic model	Studies	Studies trimmed	Fixed effects			Random effects			Q value
				Point estimate	Lower limit	Upper limit	Point estimate	Lower limit	Upper limit	
*XRCC1 Arg194Trp*	Allelic	Observed studies	-	0.00259	0.00145	0.00462	0.00259	0.00145	0.00462	19.98218
		Adjusted studies	0	0.00259	0.00145	0.00462	0.00259	0.00145	0.00462	19.98218
*XRCC1 Arg399Gln*	Heterozygous	Observed studies	-	1.10499	1.01187	1.20668	1.18413	1.00323	1.39764	81.93790
		Adjusted studies	7	0.97706	0.89922	1.06164	0.98543	0.81310	1.19427	156.42747
*XRCC1 Arg280Hi*	Allelic	Observed studies	-	1.14593	0.95931	1.36886	1.1494	0.89755	1.38496	7.91721
		Adjusted studies	0	1.14593	0.95931	1.36886	1.1494	0.89755	1.38496	7.91721
*XRCC1 Arg280Hi*	Dominant	Observed studies	-	1.16207	0.95682	1.41134	1.15301	0.93554	1.42103	7.67626
		Adjusted studies	0	1.16207	0.95682	1.41134	1.15301	0.93554	1.42103	7.67626

### 3.10 STRING results

**Figure 2:**
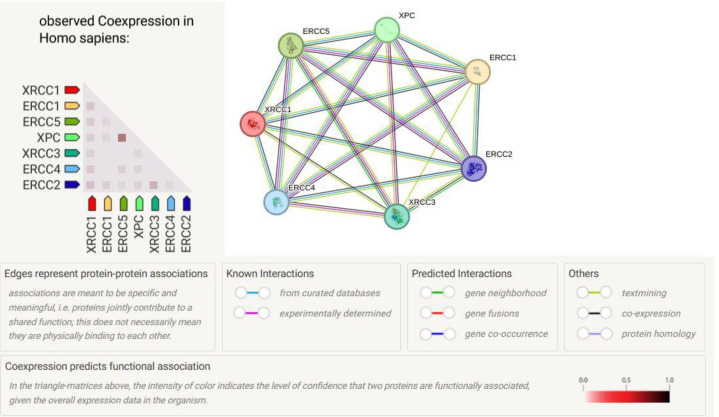
Protein-protein interaction (PPI) network graph and heatmap for *ERCC1, XPC, XPD/ERCC2, XPF/ERCC4, XPG/ERCC5, XRCC1*, and *XRCC3* genes from STRING database. The network graph represents gene interactions in the medium confidence (0.400) including the number of nodes: 7, number of edges: 21, an average of degree: 6, PPI enrichment *p-*value: < 1.0e-0.16. Each node (colored circle) represents a gene, and each edge (line) represents an interaction between two genes. The heatmap represents observed coexpression in Homo sapiens for the genes represented in the graph. Each row and column represents a gene, and each cell is colored according to the level of coexpression between the two genes it represents. Darker colors typically indicate higher levels of coexpression.

**Figure 2** shows the PPI network graph and heatmap for *ERCC1, XPC, XPD/ERCC2, XPF/ERCC4, XPG/ERCC5*, *XRCC1*, and *XRCC3* genes from the STRING database. There is curated interaction of *ERCC1* with *ERCC4, ERCC5, ERCC2*, and *XPC, ERCC4* with *ERCC5, ERCC2, XPC*, and *XPC* with *ERCC5, ERCC2, ERCC2* with *ERCC5, XRCC1* with *ERCC4, ERCC1*, *XPC*, *ERCC5*, and *ERCC2*,. In addition, there is an experimental interaction of *ERCC1* with *ERCC4, ERCC4* with *XPC*, and *ERCC2* with *ERCC5*. A coexpression analysis showed strong coexpression of *XPC* and *ERCC5, ERCC2* with *XRCC3*, and *ERCC2* with *XRCC1* (coexpression score > 0.200).

### 3.11 Gene expressions

*ERCC1, XPC, ERCC4, ERCC5*, and *XRCC1* expressions is relatively consistent across most leukemia subtypes, except in atypical CML (aCML), where it appears notably downregulated. Their reduced expression in aCML might correlate with altered DNA repair pathways or therapeutic responses. *XRCC3* and *ERCC2* expression is markedly elevated in aCML and reduced in other subtypes. The high *XRCC3* and *ERCC2* expressions in aCML may suggest a compensatory activation of DNA repair systems, possibly linked to disease aggressiveness or response to DNA-damaging therapies **Supplementary Data Sheet 4**; **Supplementary Figures S1–S7**.

Although the gene expressions (based on GEPIA2) of *ERCC1, ERCC4, ERCC5*, and *XRCC3* appears elevated in AML samples compared to normal controls and the gene expressions of *ERCC2, XPC*, and *XRCC1* appears reduced, the difference was not statistically significant based on p-value threshold (p > 0.01) (**Supplementary Data Sheet 4**; **Supplementary Figures 8**–**S14**).

Expression profiling of DNA repair genes across leukemia subtypes using BloodSpot’s MILE dataset revealed distinct transcriptional landscapes (**Supplementary Data Sheet 5**). *ERCC1* showed peak expression in Pro-B-ALL with t(11q23)/MLL, suggesting heightened nucleotide excision repair activity in this stem-like, high-risk subtype, while its lowest levels were observed in CML, indicating reduced repair demand in more differentiated lineages. *ERCC2* was most elevated in AML with MLL rearrangement, aligning with its role in transcription-coupled repair under genomic stress, and least expressed in ALL t (1, 19). *ERCC4* peaked in AML t (8, 21), a subtype with intermediate prognosis, and was lowest in Pro-B-ALL t(11q23)/MLL, hinting at subtype-specific modulation of the *ERCC1-XPF* complex. *ERCC5* and *XPC*, both involved in damage recognition, were most expressed in CLL, possibly reflecting chronic DNA damage accumulation, while their lowest expression occurred in AML inv (16) and CML, respectively. *XRCC1* showed highest expression in ALL t (1, 19) and lowest in AML with complex karyotype, suggesting a link between base excision repair activity and cytogenetic stability. Finally, *XRCC3* was most upregulated in ALL hyperdiploid, a subtype with favorable prognosis, and downregulated in AML t (15, 17), known for its distinct differentiation block. These subtype-specific expression patterns underscore the functional heterogeneity of DNA repair pathways in leukemogenesis and may inform biomarker development for risk stratification and therapeutic targeting.

### 3.12 Integrative functional and transcriptomic analyses

#### 3.12.1 Gene expression patterns across leukemia subtypes

To provide biological context for significant DNA repair polymorphisms identified in the meta-analysis, publicly available leukemia transcriptomic datasets were explored using GEPIA2, BloodSpot, and LeukemiaDB platforms. Several genes demonstrating significant genetic associations, including *ERCC2, XRCC1, XRCC3*, and *XPC*, showed variable expression patterns across leukemia subtypes. *ERCC2* and *XRCC3* tended to exhibit altered transcriptional levels in acute leukemia datasets, whereas *XRCC1* displayed heterogeneous expression profiles depending on leukemia subtype and cohort characteristics. These findings provide supportive transcriptional evidence for the involvement of DNA repair mechanisms in leukemia biology.

#### 3.12.2 Functional pathway enrichment analysis

Functional enrichment analysis of DNA repair genes included in the meta-analysis revealed significant overrepresentation of pathways related to nucleotide excision repair, base excision repair, DNA damage response signaling, and genomic stability regulation. GO biological processes highlighted cellular responses to DNA damage stimulus, chromatin organization, and replication stress pathways. These findings indicate that the genes investigated converge within core DNA repair networks implicated in leukemogenesis.

#### 3.12.3 Protein–protein interaction network analysis

Protein–protein interaction analysis using STRING demonstrated strong connectivity among ERCC1, ERCC2, ERCC4, ERCC5, XPC, XRCC1, and XRCC3 proteins, forming an interconnected DNA repair module. Central hub interactions were observed around ERCC2 and XRCC1, supporting their potential functional importance within nucleotide and base excision repair pathways. The network topology suggests coordinated regulation of DNA repair responses contributing to leukemia susceptibility.

## 4 Discussion

Consistent with the pooled analyses, high between-study heterogeneity was observed in several genetic models, particularly for *XRCC1 Arg194Trp, XRCC1 Arg399Gln*, and *XRCC3 Thr241Met* polymorphisms (I² > 60%). Multiple methodological and biological factors may contribute to this variability. First, substantial differences in ethnicity and genetic background across included populations (Asian, Caucasian, age group, and mixed populations) may influence allele frequencies and gene–environment interactions, leading to inconsistent effect sizes. Second, the included studies encompassed heterogeneous leukemia subtypes (AML, ALL, CML, and CLL), which have distinct molecular mechanisms and risk profiles; pooling these entities may increase statistical variability. Third, variations in study design, including hospital-based versus population-based controls, differences in age groups (adult vs pediatric populations), and disparities in sample size, may also introduce heterogeneity. Fourth, methodological differences such as genotyping techniques (PCR-RFLP, TaqMan, sequencing) and deviations from HWE in some control groups could further contribute to between-study inconsistencies. Although subgroup analyses partially reduced heterogeneity in certain comparisons, residual variability remained, suggesting that additional unmeasured factors, such as environmental exposures, lifestyle factors, and gene–gene interactions, may influence the associations. Therefore, the pooled estimates should be interpreted with caution, particularly for models with high heterogeneity. The use of random-effects models helps account for variability; however, the observed heterogeneity may limit the generalizability and robustness of some conclusions.

We examined the associations between various DNA repair gene polymorphisms and leukemia risk, highlighting the significant roles of *ERCC2 Lys751Gln, XPC Lys939Gln, XRCC1 Arg194Trp, XRCC1 Arg399Gln*, and *XRCC3 Thr241Met* in different populations and leukemia subtypes. Our findings suggest that ethnicity, sample size, age group, and leukemia subtype influence these associations, emphasizing the complexity of genetic susceptibility. While some polymorphisms demonstrated robust links to leukemia risk, others showed no significant effects, reinforcing the need for cautious interpretation due to study heterogeneity and potential publication bias. These results support the importance of genetic variations in DNA repair pathways in leukemia development and highlight the need for further research with larger, well-controlled studies to validate these associations and explore their functional implications.

Expression profiling across leukemia subtypes using BloodSpot’s MILE dataset revealed distinct transcriptional landscapes for DNA repair genes, reinforcing their subtype-specific modulation. Notably, *aCML* exhibited a dual dysregulation pattern—marked downregulation of *ERCC1*, *XPC*, *ERCC4*, *ERCC5*, and *XRCC1*, alongside upregulation of *XRCC3* and *ERCC2*—suggesting compensatory shifts in repair pathway engagement. Complementary analysis highlighted peak expression of *ERCC1* in Pro-B-ALL with t(11q23)/MLL and lowest in CML, while *XRCC3* was most elevated in ALL hyperdiploid and suppressed in AML t (15, 17), underscoring functional heterogeneity across cytogenetic contexts. Although AML samples showed directional trends compared to healthy bone marrow, statistical insignificance (p > 0.01) limits broader inference, warranting further validation in expanded cohorts.

The multi-dataset gene expression component of this study was intended as an exploratory and hypothesis-generating analysis rather than a formal integrative meta-analysis. Publicly available datasets were analyzed descriptively to identify transcriptional patterns across leukemia subtypes, without quantitative cross-platform normalization, pooled statistical modeling, or effect size aggregation. Therefore, the expression findings should be interpreted cautiously and considered complementary to the genetic meta-analysis rather than confirmatory evidence. Differences in dataset composition, sample processing, and platform heterogeneity may limit direct comparability across cohorts. Future studies employing standardized multi-cohort integration pipelines and independent validation datasets are required to establish robust transcriptomic–genetic relationships.

NER is a versatile system that monitors and repairs DNA damage caused by both internal and external factors. Polymorphisms in DNA repair genes may alter the function and/or efficiency of DNA repair, contributing to individual variations in DNA repair capacity (25, 101, 102). Although alterations in NER pathway activity may have therapeutic implications, the present study does not directly evaluate therapeutic targeting or inhibitor development, and such interpretations should be considered speculative. Polymorphisms in core *NER* genes can influence NER capacity by affecting the expression and function of key proteins, thereby impacting the survival of leukemia patients (48, 52, 73). Variations in the coding and regulatory regions of DNA repair genes may modify gene expression, leading to changes in DNA repair capacity and potentially contributing to a cell’s susceptibility to genotoxic agents and malignancy (103).

ERCC1/XPF is essential for removing DNA interstrand cross-links (ICLs), and deficiencies in XPF and ERCC1 result in extreme hypersensitivity to ICLs (104, 105). In NER, ERCC1/XPF is responsible for making an incision 5′ to the lesion (106), and in ICL repair, it is required for the unhooking incision of ICLs (107). Analyses indicate that XPF-ERCC1 has significant functions beyond its central role in NER crosslink repair, which is necessary to prevent endogenous DNA damage (108). In addition, several studies showed that both *XRCC1* and *ERCC1* variants can be associated with the risk of cancers (109–111). This supports the protective role of these polymorphisms in leukemia, especially when combined, as STRING analysis has shown interactions between them.

A 2013 meta-analysis (38) examined the association between three *XRCC1* polymorphisms (*Arg194Trp, Arg399Gln*, and *Arg280His*) and leukemia risk. The findings suggested that these polymorphisms might not be linked to overall leukemia risk. Another meta-analysis (41) investigated the *XRCC3 Thr241Met* polymorphism and its potential connection to leukemia. The results indicated that while this polymorphism did not increase leukemia risk in African and Asian populations, it was associated with a higher risk in Caucasian populations. However, our meta-analysis yielded conflicting results, revealing that *XRCC1 Arg194Trp, XRCC1 Arg399Gln*, and *XRCC3 Thr241Met* polymorphisms were linked to an overall increased risk of leukemia. Additionally, the *XRCC3 Thr241Met* polymorphism was found to contribute to a higher leukemia risk in both Asian and Caucasian populations.

The findings of the present meta-analysis differ from those of the 2013 meta-analysis (38), which reported no overall association between *XRCC1* polymorphisms and leukemia risk. Several methodological and evidence-based factors may explain these discrepancies. First, the current analysis includes a substantially larger and more recent pool of studies, increasing statistical power and improving the precision of pooled estimates compared with the earlier analysis, which included only 19 studies for *Arg399Gln, 12 for Arg194Trp*, and *6 for Arg280His*. Second, differences in ethnic composition and expanded representation of certain populations in the present study may have influenced the observed associations, given the known variability in allele frequencies and gene–environment interactions across ethnic groups. Third, the earlier meta-analysis combined heterogeneous leukemia subtypes with relatively small subgroup sample sizes, which may have diluted subtype-specific effects, whereas the current study incorporated more detailed stratified analyses that allowed detection of associations within specific leukemia types. Finally, advances in analytical methodology, including more comprehensive heterogeneity assessment, updated publication bias correction, and expanded sensitivity analyses, may have contributed to the identification of associations not observed in earlier work. These differences highlight the importance of continuously updating genetic meta-analyses as new data emerge.

The meta-regression analyses provide important insights into the sources of heterogeneity and the reliability of observed associations. The negative association between methodological quality and effect size for XRCC3 Thr241Met suggests that lower-quality studies may overestimate genetic risk, emphasizing the importance of rigorous study design in genetic association research. Similarly, the influence of sample size on XRCC1 Arg194Trp and XRCC3 Thr241Met indicates potential small-study effects, where smaller investigations may report inflated associations. The significant effect of publication year further suggests temporal trends, possibly reflecting improvements in study methodology, genotyping accuracy, or reporting standards over time. Collectively, these findings indicate that variability in methodological rigor and study characteristics contributes to heterogeneity and should be considered when interpreting pooled effect estimates.

A number of included studies demonstrated deviation from HWE in control groups, which may reflect potential genotyping errors, population stratification, selection bias, or non-representative sampling. Because HWE deviation is considered an important indicator of methodological quality in genetic association studies, additional sensitivity analyses were performed after excluding these studies. The pooled estimates remained stable, suggesting that the observed associations were robust and not driven by studies with potential methodological concerns. Nevertheless, the presence of HWE deviation highlights the need for cautious interpretation and emphasizes the importance of rigorous genotyping procedures and appropriate control selection in future research.

Interactions between polymorphisms in *XPC* and *XPD* may act as a risk factor for cancers (112). One study found that dual variant genotypes of the *XPD* gene continued to confer an increased risk of AML, even when combined with the variant *XPC* gene alone or with both *XPC* and *XPG* variant genotypes (113). This aligns with the increased risk of these polymorphisms for leukemia, particularly in combination, as STRING analysis has shown interactions between them.

The results indicate that further investigations across different ethnicities are necessary to clarify this issue. Evidence suggests that socioeconomic status and birthplace may influence susceptibility to leukemia, and clinical types might also play key roles in its development (114). There is ethnic variability in the allelic distribution of *XPD* polymorphisms (80). Our meta-analysis found that ethnicity, sample size, control source, age group, and leukemia subtype were significant factors for *ERCC2 Lys751Gln* and *XPC Lys939Gln* polymorphisms. The observed stronger association of the *XRCC1 Arg194Trp* polymorphism in Caucasian populations may reflect ethnic differences in allele frequencies, linkage disequilibrium patterns with functional variants, and gene–environment interactions that influence DNA repair capacity. Population-specific environmental exposures and genetic backgrounds may modify the functional impact of *XRCC1* variants, leading to differential susceptibility across ethnic groups. In addition, the more pronounced association observed in AML compared with other leukemia subtypes may be explained by the biological characteristics of AML, which is marked by high levels of genomic instability and a strong dependence on base excision repair mechanisms. *XRCC1* plays a central role in coordinating single-strand break repair; therefore, functional alterations associated with the *Arg194Trp* variant may disproportionately affect hematopoietic progenitor cells undergoing rapid proliferation and differentiation in AML. These subtype-specific molecular contexts may amplify the impact of impaired DNA repair capacity on leukemogenesis. Additionally, another meta-analysis reported that ethnicity and leukemia subtype were significant factors for the association between the *ERCC2 Lys751Gln* polymorphism and leukemia risk (36).

While our meta-analysis identified significant associations between several DNA repair gene polymorphisms (ERCC1 8092C>A, ERCC2 Lys751Gln, XPC Lys939Gln, XRCC1 Arg194Trp/Arg399Gln, and XRCC3 Thr241Met) and leukemia susceptibility, and gene expression data provided exploratory insights into subtype-specific transcriptional pattern, we cannot directly infer a causal genotype-phenotype relationship. The observed overlap between risk alleles and differential gene expression is hypothesis-generating, suggesting that inherited variations in DNA repair genes may influence transcriptional activity and leukemia subtype characteristics, but experimental validation is required. Future studies integrating genetic, transcriptomic, and functional assays are necessary to clarify whether these polymorphisms directly modulate gene expression, DNA repair capacity, and therapeutic response across leukemia subtypes.

However, our research also has some limitations (1): The number of original studies included in the meta-analysis is relatively small for several polymorphisms, particularly those with only two studies (e.g., *ERCC1 8092C>A*), which limits the reliability and generalizability of the pooled estimates. Future studies with larger sample sizes and more diverse populations, considering ethnicity, age, and leukemia subtype, are needed to further corroborate our findings (2). Heterogeneity was high in some models, likely due to differences in sample sizes, study design, and population characteristics. Notably, heterogeneity estimates (e.g., I²) may be unreliable for polymorphisms with very few studies, reducing confidence in the pooled results (3). The multi-dataset expression analysis was exploratory and did not involve formal quantitative integration across cohorts. Differences in study design, platform technology, and sample characteristics may introduce bias and limit reproducibility. Consequently, these findings should be interpreted as descriptive observations requiring independent validation.

## 6 Conclusions

This study provides a comprehensive synthesis of the associations between DNA repair gene polymorphisms and leukemia susceptibility, highlighting several variants—*XPD/ERCC2 Lys751Gln, XPC Lys939Gln, XRCC1 Arg194Trp, XRCC1 Arg399Gln*, and *XRCC3 Thr241Met*—that consistently show increased leukemia risk across specific genetic models. Subgroup analyses reveal that ethnicity, leukemia subtype, age group, and sample size significantly modulate these associations, underscoring the complexity of genetic susceptibility in hematological malignancies. TSA further strengthened the interpretation of the meta-analysis. Robust cumulative evidence was confirmed for polymorphism–model combinations where the Z-curve crossed the monitoring boundary and required information size, indicating sufficient statistical power. However, several genetic models did not reach the required information size, highlighting that some observed associations remain preliminary and require further investigation through larger and methodologically rigorous studies.

Integration of transcriptomic data indicates subtype-specific expression patterns—for example, upregulation of XRCC3 and ERCC2 in aCML and reduced ERCC1, XPC, and XRCC1 in certain AML and CML subtypes—highlighting potential functional heterogeneity and altered DNA repair dynamics. While these patterns suggest avenues for biomarker development and therapeutic stratification, causal genotype–phenotype relationships remain to be experimentally validated.

Clinically, *XPD/ERCC2 Lys751Gln, XPC Lys939Gln, XRCC1 Arg194Trp, XRCC1 Arg399Gln*, and *XRCC3 Thr241Met* polymorphisms may serve as risk markers for leukemia, particularly in Caucasian and Asian populations and in AML and ALL subtypes where associations were strongest. Individuals carrying these risk alleles could benefit from enhanced surveillance for early detection, and knowledge of their genotype may inform personalized treatment strategies, such as predicting sensitivity to DNA-damaging chemotherapeutics targeting impaired repair pathways. However, due to high heterogeneity in some models, limited study numbers for certain variants, and potential publication bias, these applications should be considered preliminary until validated in larger, multi-ethnic cohorts with standardized methodologies.

Future research should focus on large, multi-ethnic cohorts, standardized genotyping methodologies, and functional studies to clarify the biological impact of these polymorphisms. Combining genetic, transcriptomic, and environmental data could enable more accurate risk assessment models and guide precision medicine strategies in leukemia management.

## Data Availability

The original contributions presented in the study are included in the article/**Supplementary Material**, Further inquiries can be directed to the corresponding author.
