# Supplementary material for: A meta-analysis and expression profiling of DNA repair gene polymorphisms in leukemia
Source: Front Oncol. 2026 Apr 23;16:1777198. doi: 10.3389/fonc.2026.1777198 (PMC13149071; doi:10.3389/fonc.2026.1777198)
Supplement: Supplementary file 1 [file DataSheet1.docx]

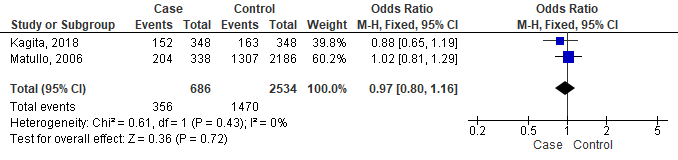

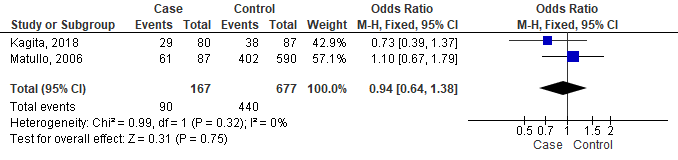

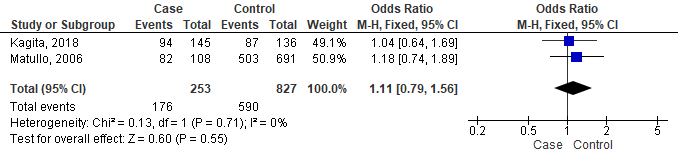

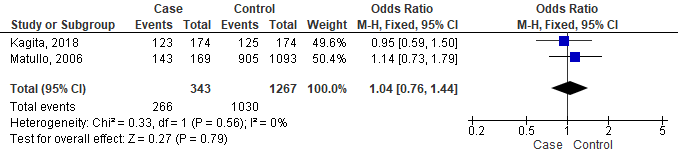

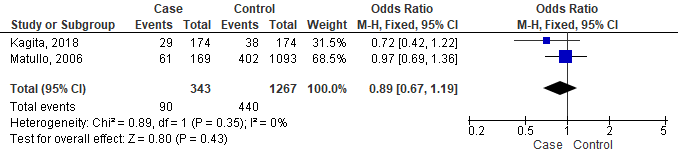


**E**

**D**

**C**

**B**

**A**

**Figure 1S**: Forest plot analysis of the association between *ERCC1 C118T* polymorphism and the risk of leukemia in: A) allelic model, B) homozygous model, C) heterozygous model, D) dominant model, and E) recessive model.


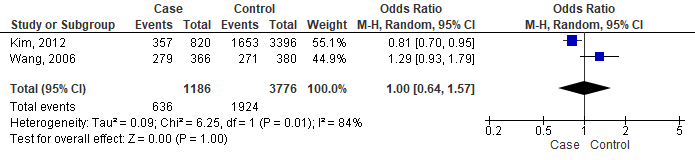

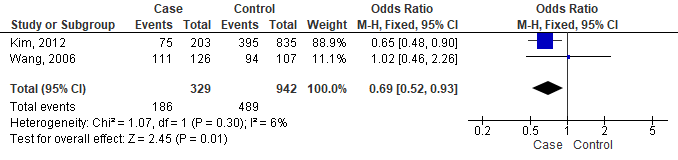

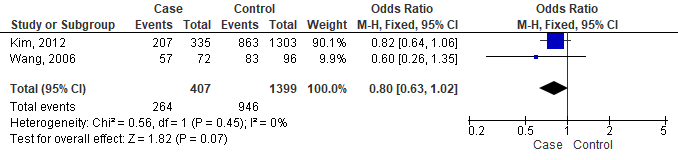

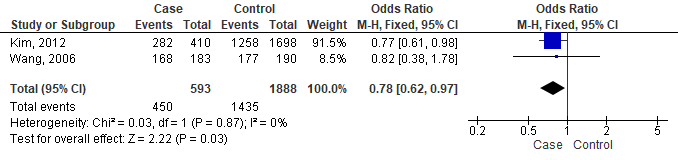

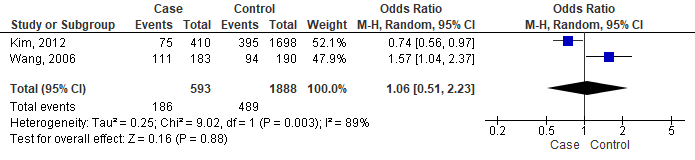


**E**

**D**

**C**

**B**

**A**

**Figure 2S**: Forest plot analysis of the association between *ERCC1 8092C>A* polymorphism and the risk of leukemia in: A) allelic model, B) homozygous model, C) heterozygous model, D) dominant model, and E) recessive model.


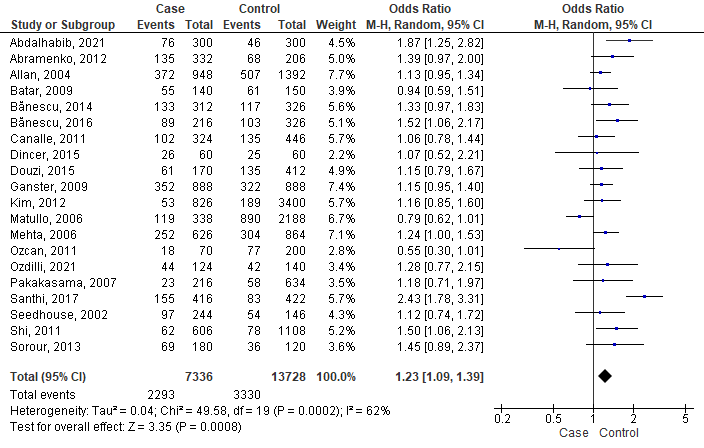


**Figure 3S**: Forest plot analysis of the association between *XPD/ERCC2 Lys751Gln* polymorphism and the risk of leukemia in allelic model


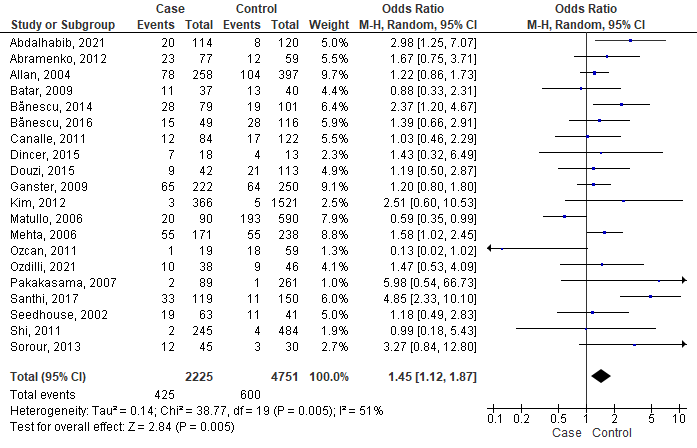


**Figure 4S**: Forest plot analysis of the association between *XPD/ERCC2 Lys751Gln* polymorphism and the risk of leukemia in homozygous model


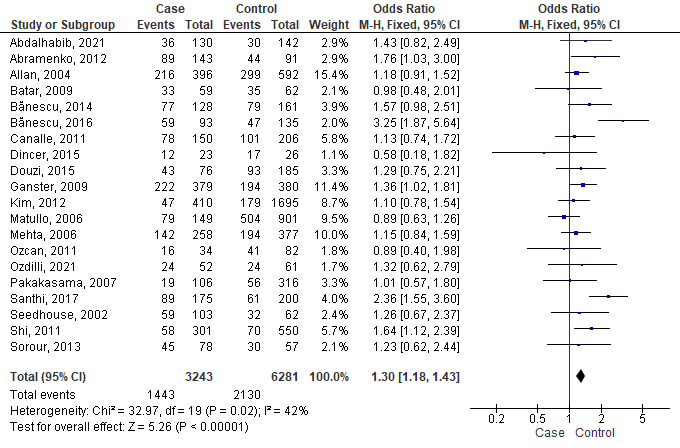


**Figure 5S**: Forest plot analysis of the association between *XPD/ERCC2 Lys751Gln* polymorphism and the risk of leukemia in heterozygous model


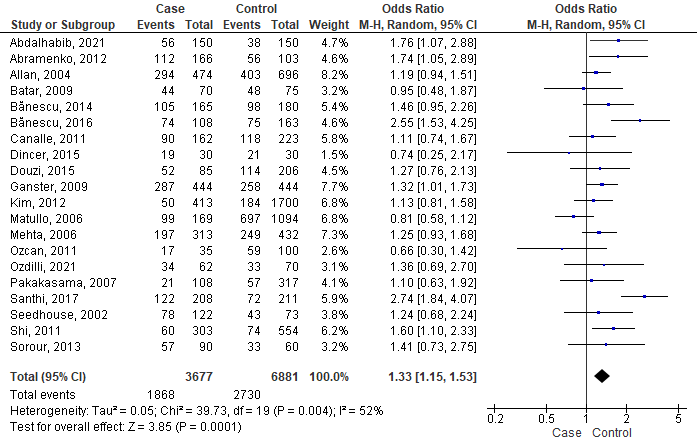


**Figure 6S**: Forest plot analysis of the association between *XPD/ERCC2 Lys751Gln* polymorphism and the risk of leukemia in dominant model


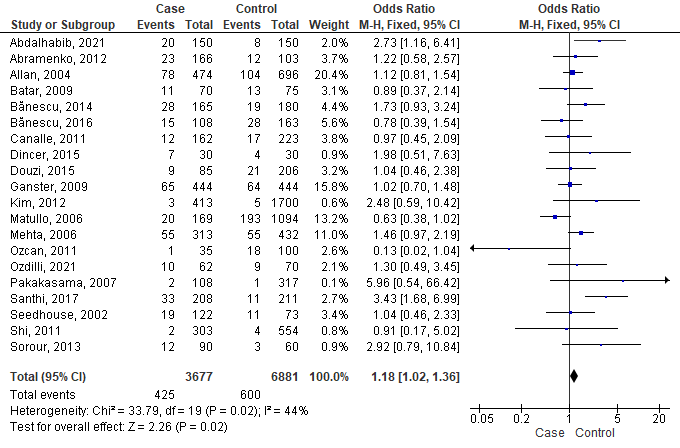


**Figure 7S**: Forest plot analysis of the association between *XPD/ERCC2 Lys751Gln* polymorphism and the risk of leukemia in recessive model


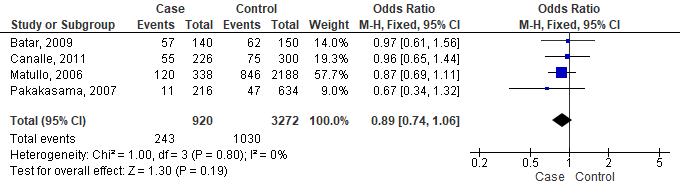

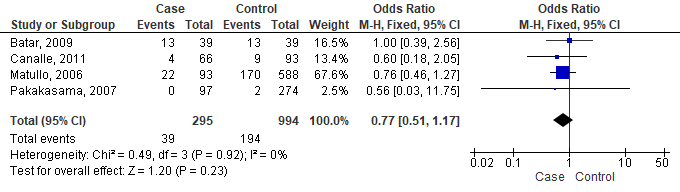

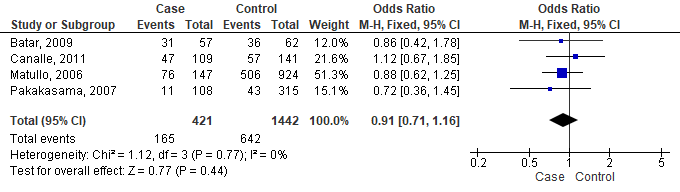

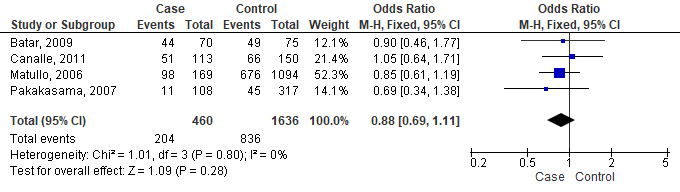

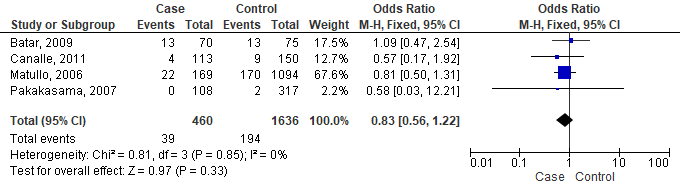


**E**

**D**

**C**

**B**

**A**

**Figure 8S**: Forest plot analysis of the association between *XPD/ERCC2 Asp312Asn* polymorphism and the risk of leukemia in: A) allelic model, B) homozygous model, C) heterozygous model, D) dominant model, and E) recessive model.


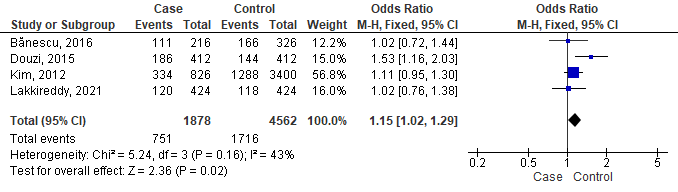

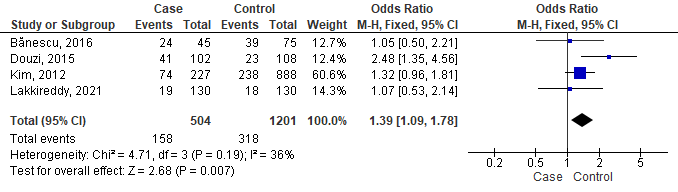

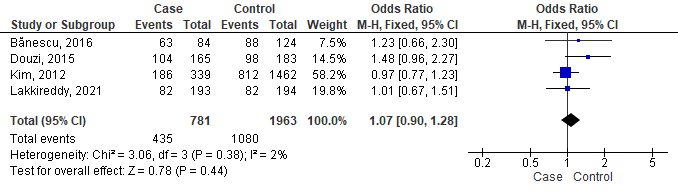

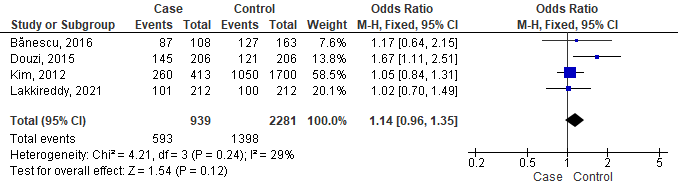

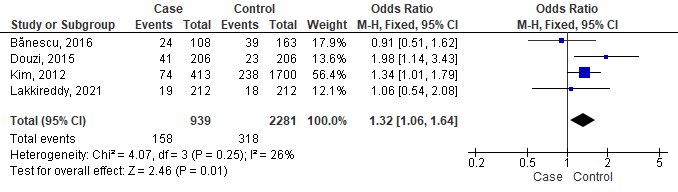


**E**

**D**

**C**

**B**

**A**

**Figure 9S**: Forest plot analysis of the association between *XPC Lys939Gln* polymorphism and the risk of leukemia in: A) allelic model, B) homozygous model, C) heterozygous model, D) dominant model, and E) recessive model.


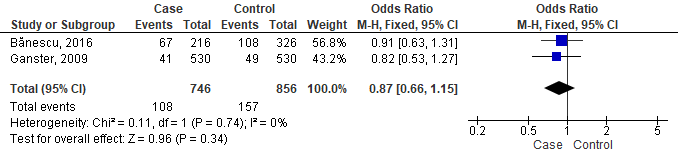

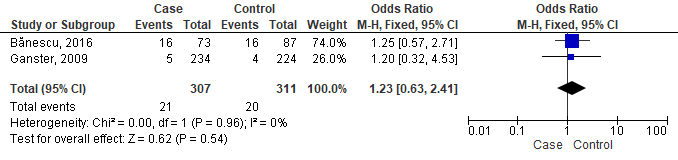

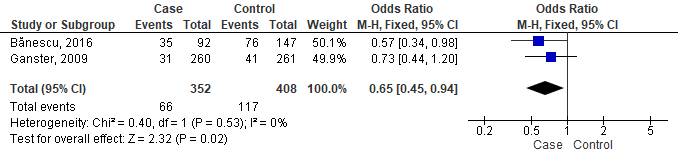

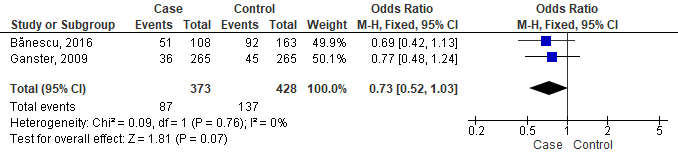

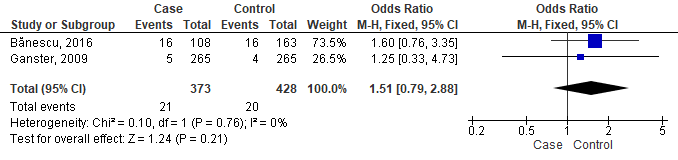


**E**

**D**

**C**

**B**

**A**

**Figure 10S**: Forest plot analysis of the association between *XPG 3507G > C* polymorphism and the risk of leukemia in: A) allelic model, B) homozygous model, C) heterozygous model, D) dominant model, and E) recessive model.


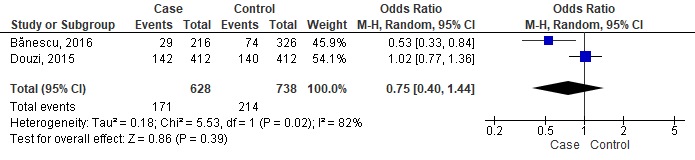

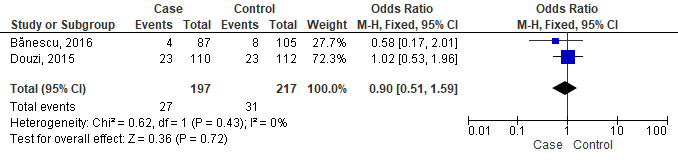

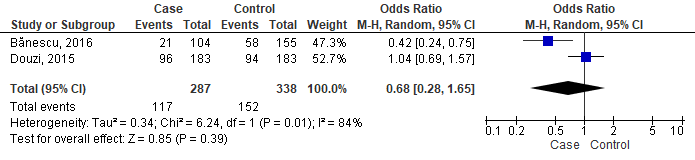

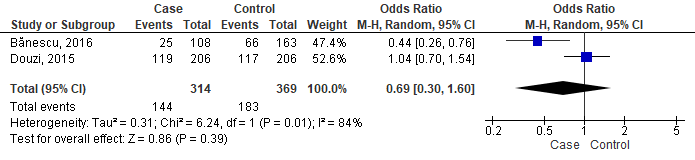

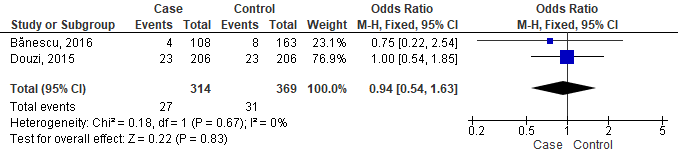


**E**

**D**

**C**

**B**

**A**

**Figure 11S**: Forest plot analysis of the association between *XPF/ERCC4 Arg415Gln* polymorphism and the risk of leukemia in: A) allelic model, B) homozygous model, C) heterozygous model, D) dominant model, and E) recessive model.

**
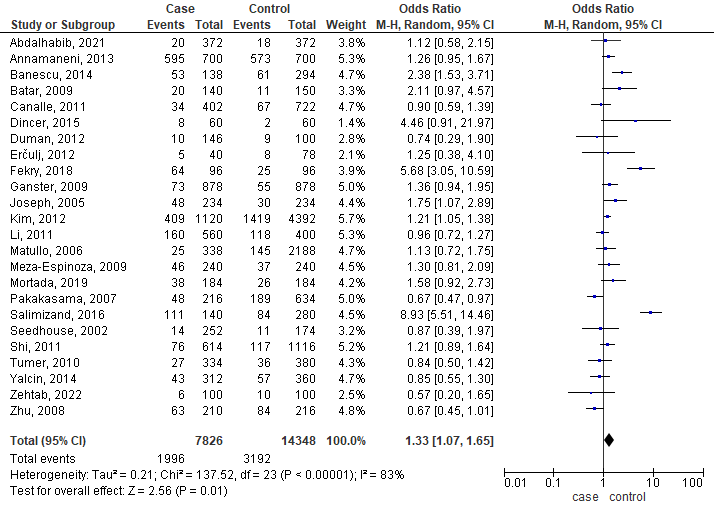
**

**Figure 12S**: Forest plot analysis of the association between *XRCC1 Arg194Trp (rs1799782)* polymorphism and the risk of leukemia in allelic model


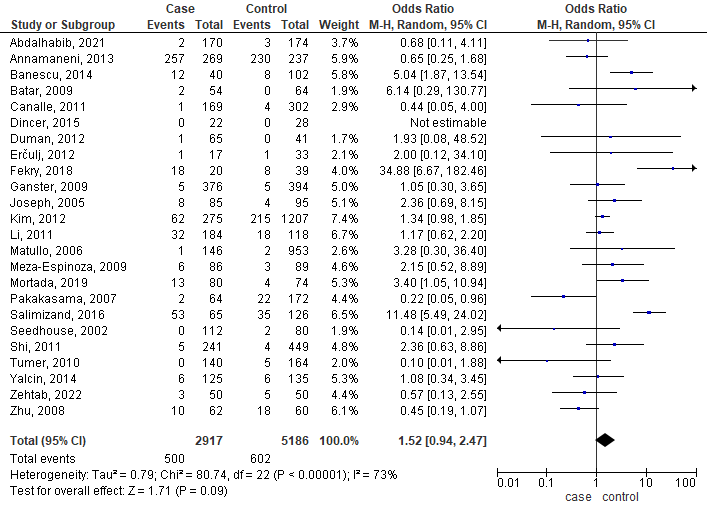
 **Figure 13S**: Forest plot analysis of the association between *XRCC1 Arg194Trp (rs1799782)* polymorphism and the risk of leukemia in homozygous model


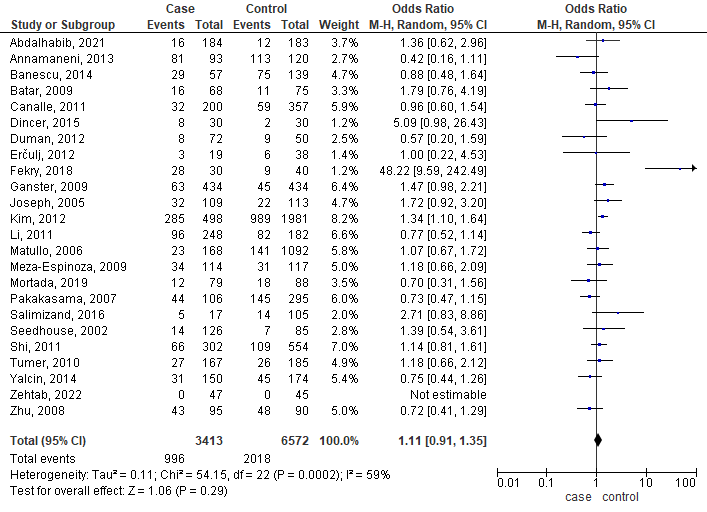


**Figure 14S**: Forest plot analysis of the association between *XRCC1 Arg194Trp (rs1799782)* polymorphism and the risk of leukemia in heterozygous model


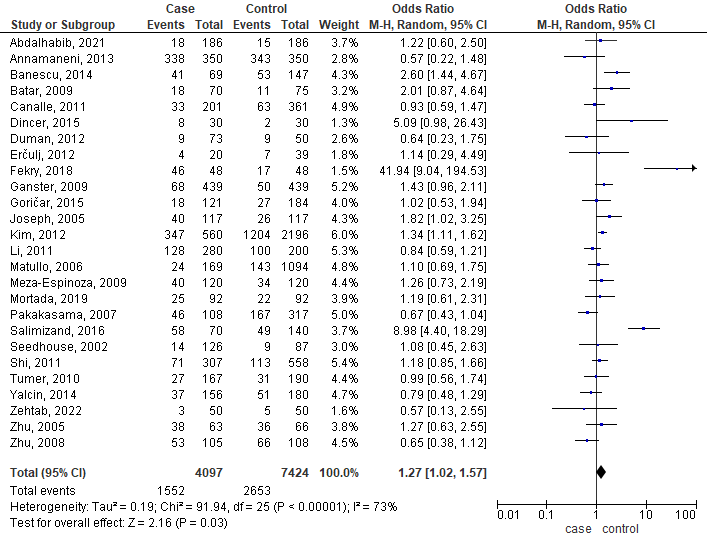


**Figure 15S**: Forest plot analysis of the association between *XRCC1 Arg194Trp (rs1799782)* polymorphism and the risk of leukemia in dominant model


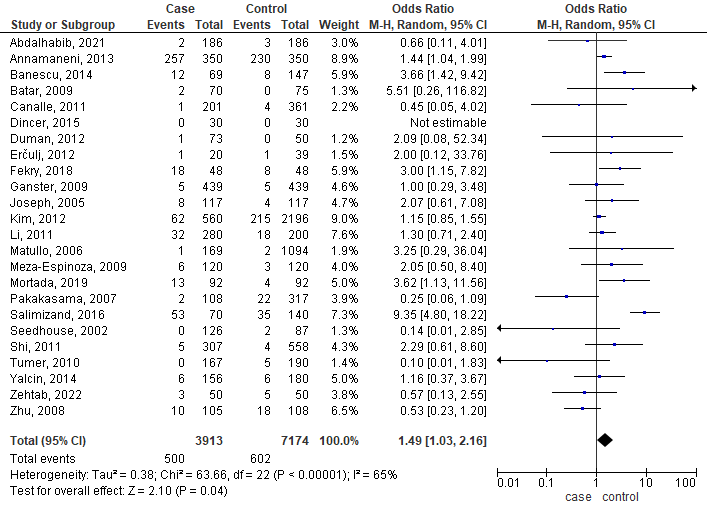


**Figure 16S**: Forest plot analysis of the association between *XRCC1 Arg194Trp (rs1799782)* polymorphism and the risk of leukemia in recessive model


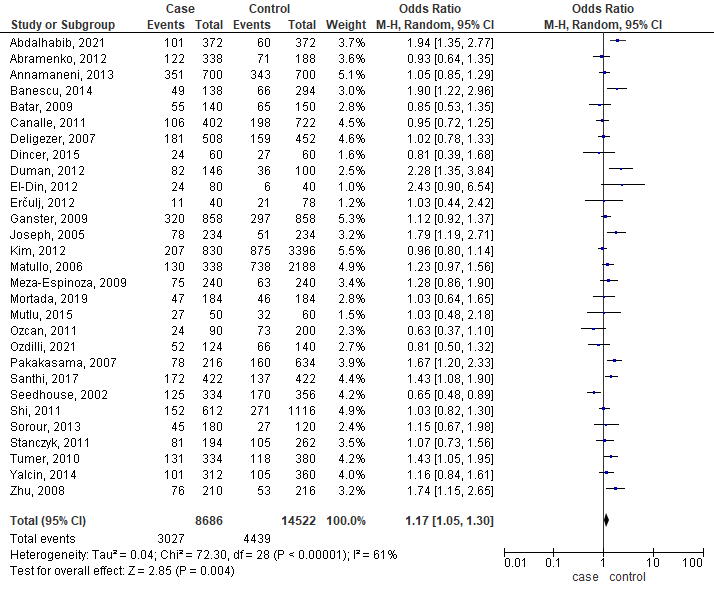


**Figure 17S**: Forest plot analysis of the association between *XRCC1 Arg399Gln (rs25487)* polymorphism and the risk of leukemia in allelic model


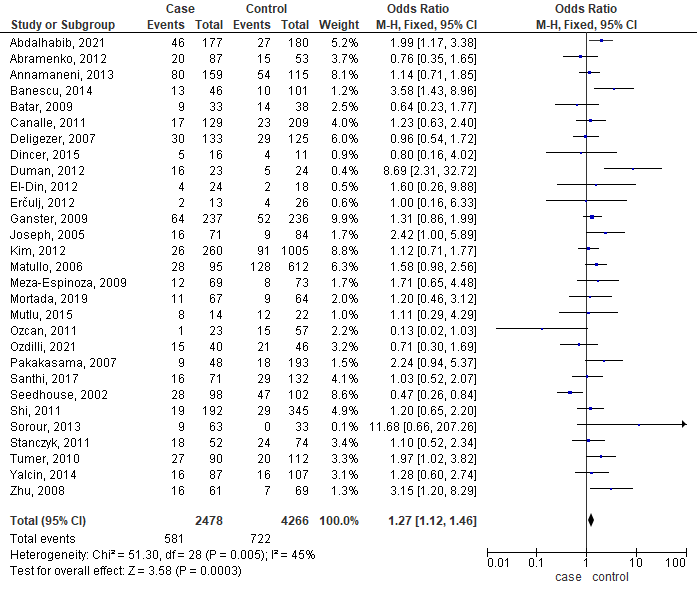
 **Figure 18S**: Forest plot analysis of the association between *XRCC1 Arg399Gln (rs25487)* polymorphism and the risk of leukemia in homozygous model


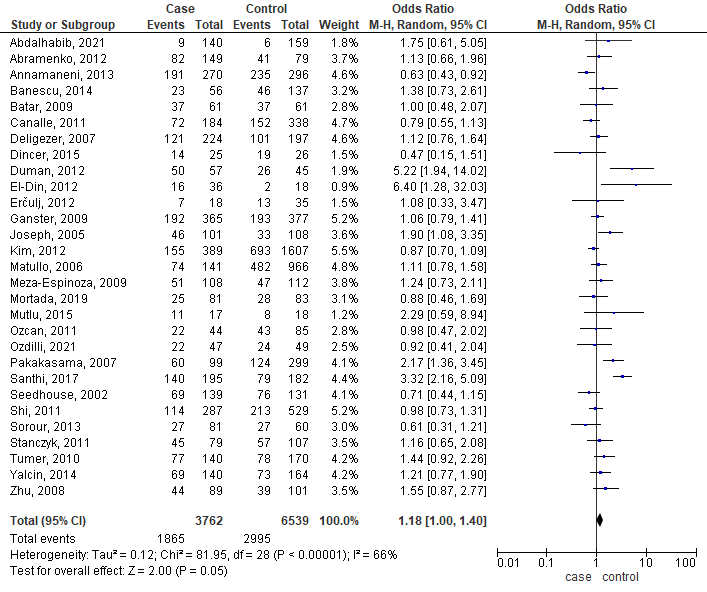


**Figure 19S**: Forest plot analysis of the association between *XRCC1 Arg399Gln (rs25487)* polymorphism and the risk of leukemia in heterozygous model


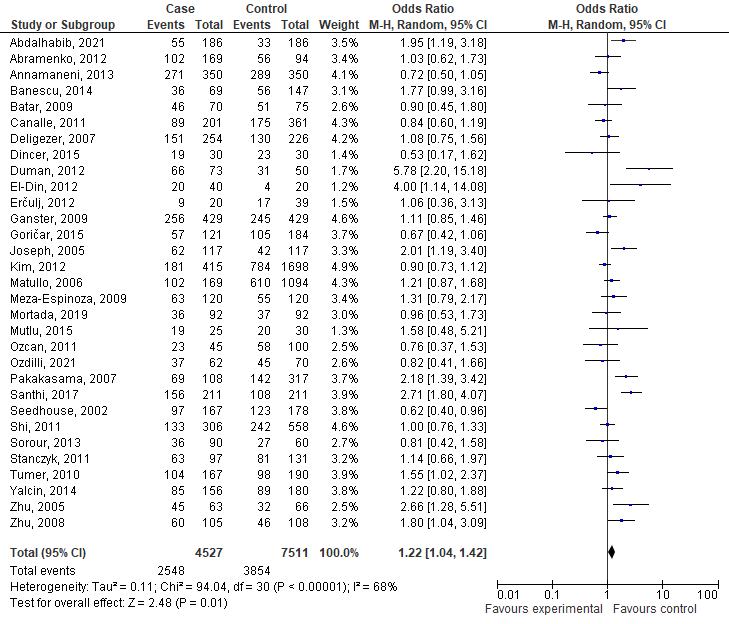


**Figure 20S**: Forest plot analysis of the association between *XRCC1 Arg399Gln (rs25487)* polymorphism and the risk of leukemia in dominant model


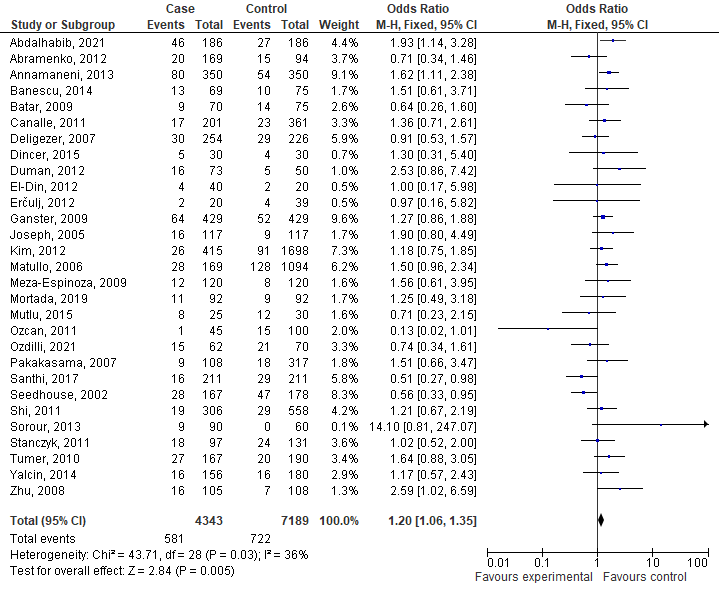


**Figure 21S**: Forest plot analysis of the association between *XRCC1 Arg399Gln (rs25487)* polymorphism and the risk of leukemia in recessive model


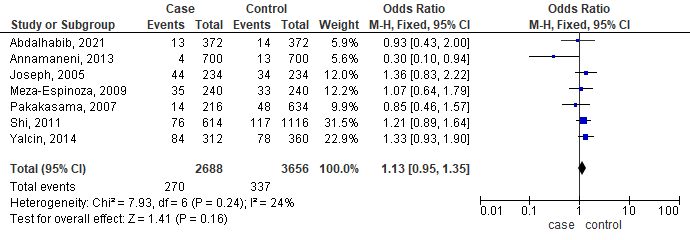


**Figure 22S**: Forest plot analysis of the association between *XRCC1 Arg280His (rs25489)* polymorphism and the risk of leukemia in allelic model


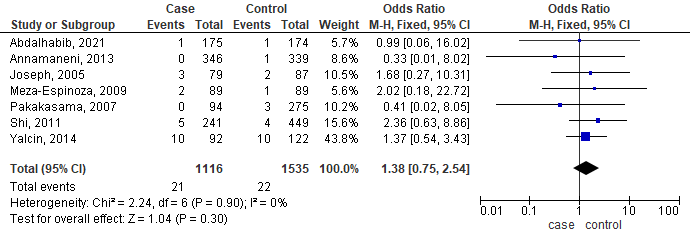


**Figure 23S**: Forest plot analysis of the association between *XRCC1 Arg280His (rs25489)* polymorphism and the risk of leukemia in homozygous model


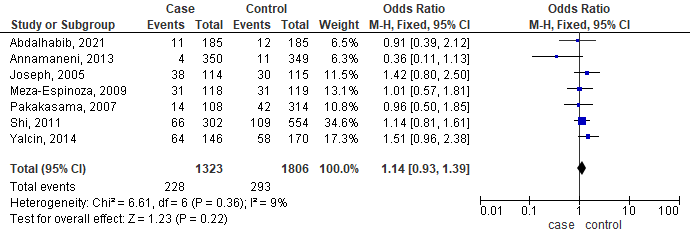


**Figure 24S**: Forest plot analysis of the association between *XRCC1 Arg280His (rs25489)* polymorphism and the risk of leukemia in heterozygous model


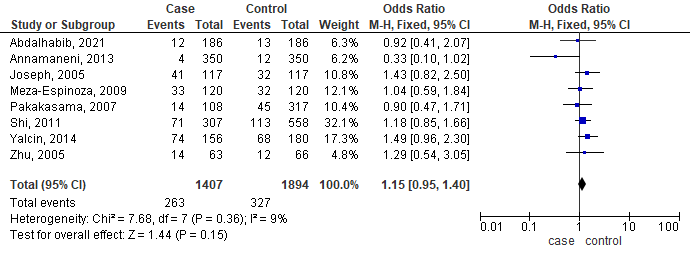


**Figure 25S**: Forest plot analysis of the association between *XRCC1 Arg280His (rs25489)* polymorphism and the risk of leukemia in dominant model


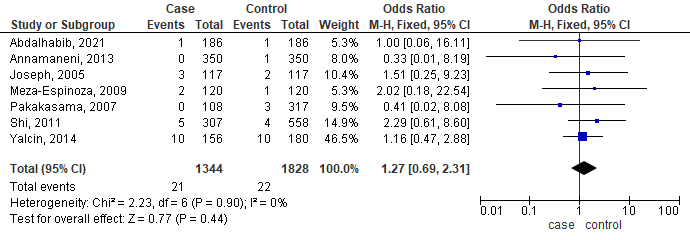


**Figure 26S**: Forest plot analysis of the association between *XRCC1 Arg280His (rs25489)* polymorphism and the risk of leukemia in recessive model


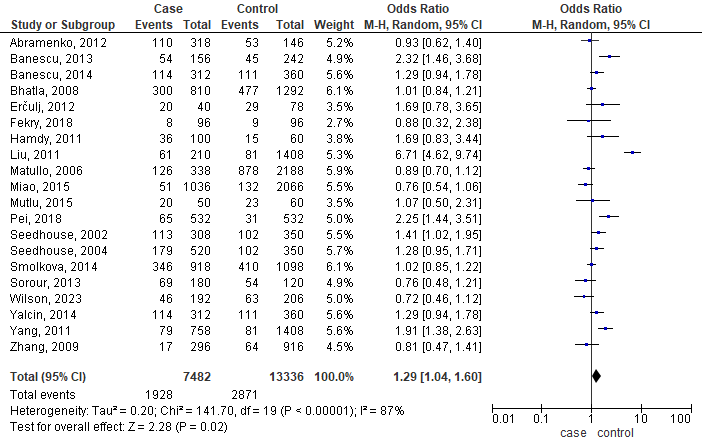


**Figure 27S**: Forest plot analysis of the association between *XRCC3 Thr241Met (rs861539)* polymorphism and the risk of leukemia in allelic model


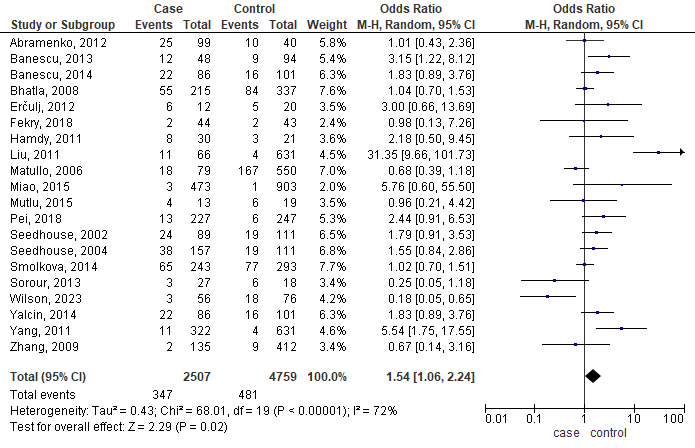


**Figure 28S**: Forest plot analysis of the association between *XRCC3 Thr241Met (rs861539)* polymorphism and the risk of leukemia in homozygous model


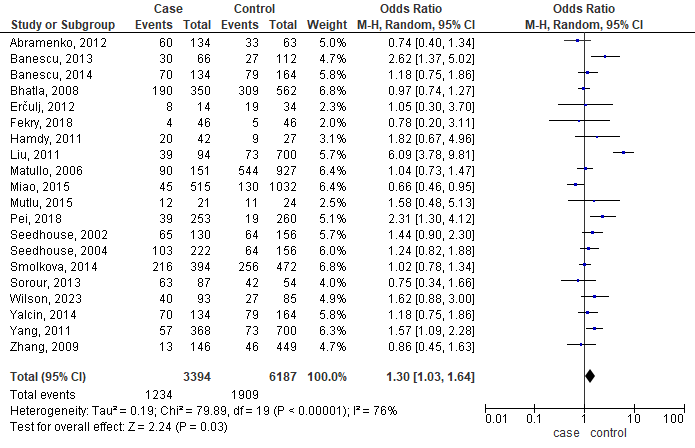


**Figure 29S**: Forest plot analysis of the association between *XRCC3 Thr241Met (rs861539)* polymorphism and the risk of leukemia in heterozygous model


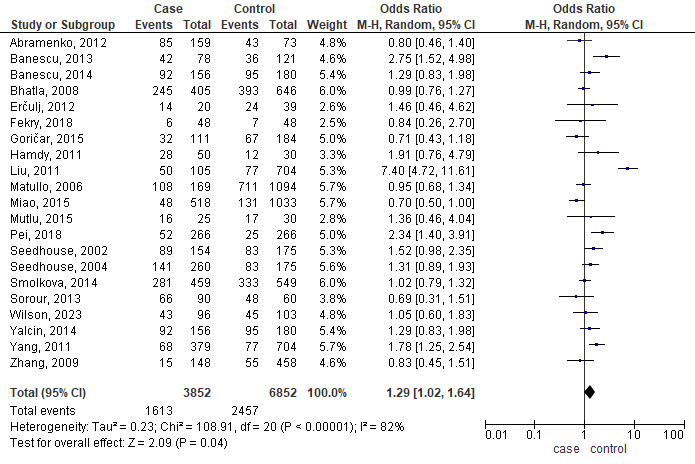


**Figure 30S**: Forest plot analysis of the association between *XRCC3 Thr241Met (rs861539)* polymorphism and the risk of leukemia in dominant model


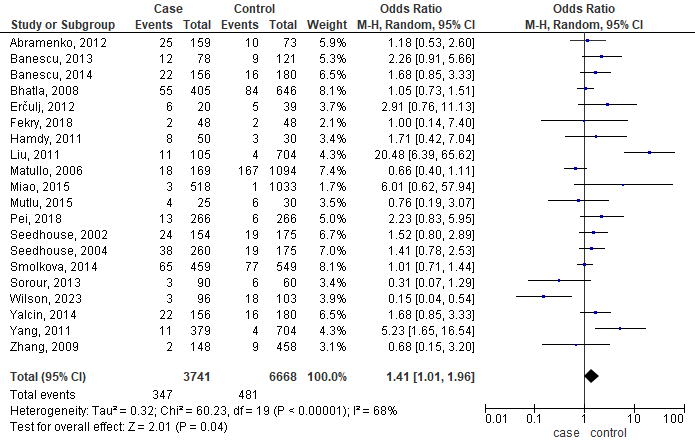


**Figure 31S**: Forest plot analysis of the association between *XRCC3 Thr241Met (rs861539)* polymorphism and the risk of leukemia in recessive model
