# Supplementary material for: A meta-analysis and expression profiling of DNA repair gene polymorphisms in leukemia
Source: Front Oncol. 2026 Apr 23;16:1777198. doi: 10.3389/fonc.2026.1777198 (PMC13149071; doi:10.3389/fonc.2026.1777198)
Supplement: Supplementary file 2 [file DataSheet2.docx]

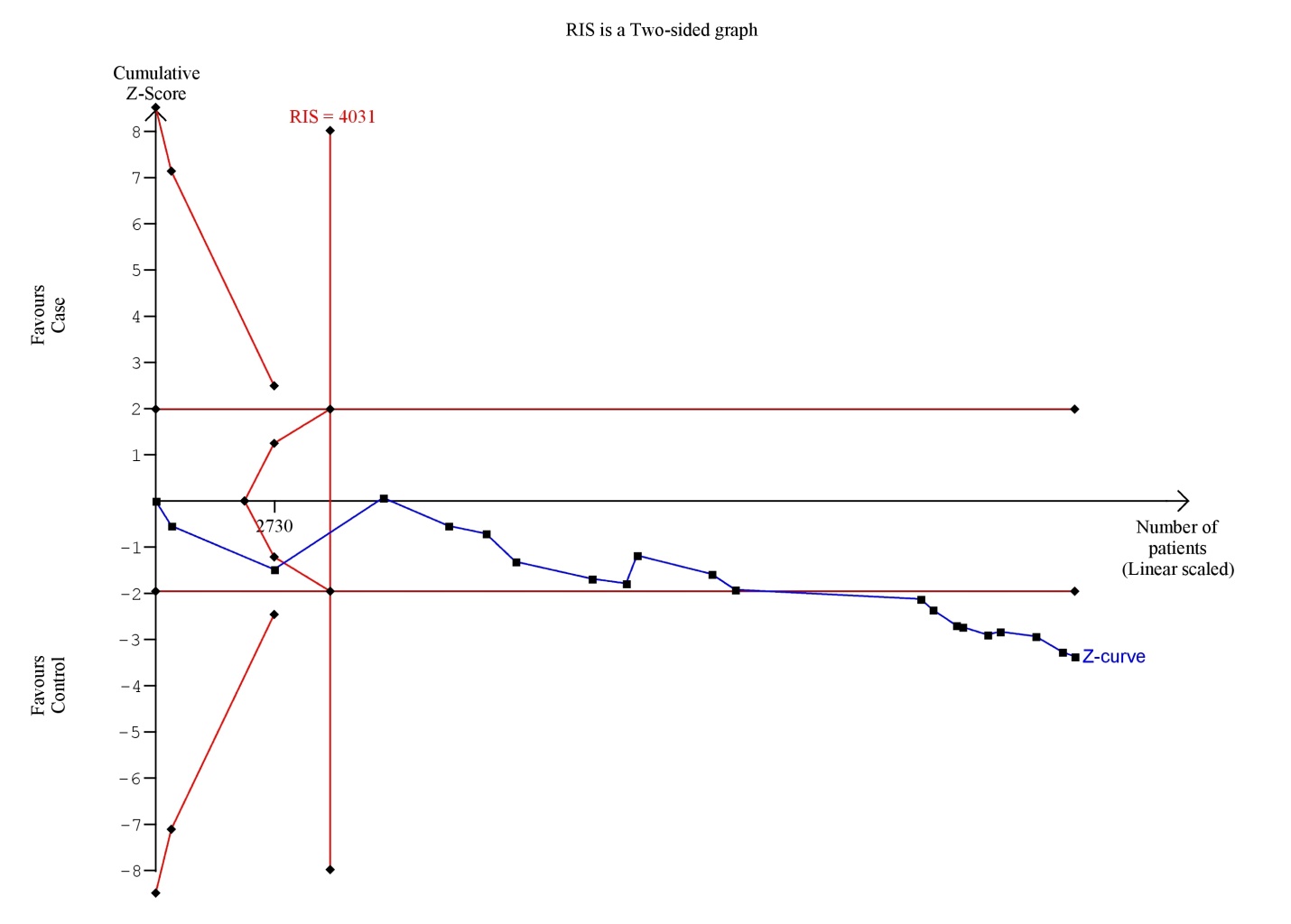


**Figure 1S**: Trial sequential analysis of the association between *ERCC2* *Lys751Gln* polymorphism and the risk of leukemia in allelic model


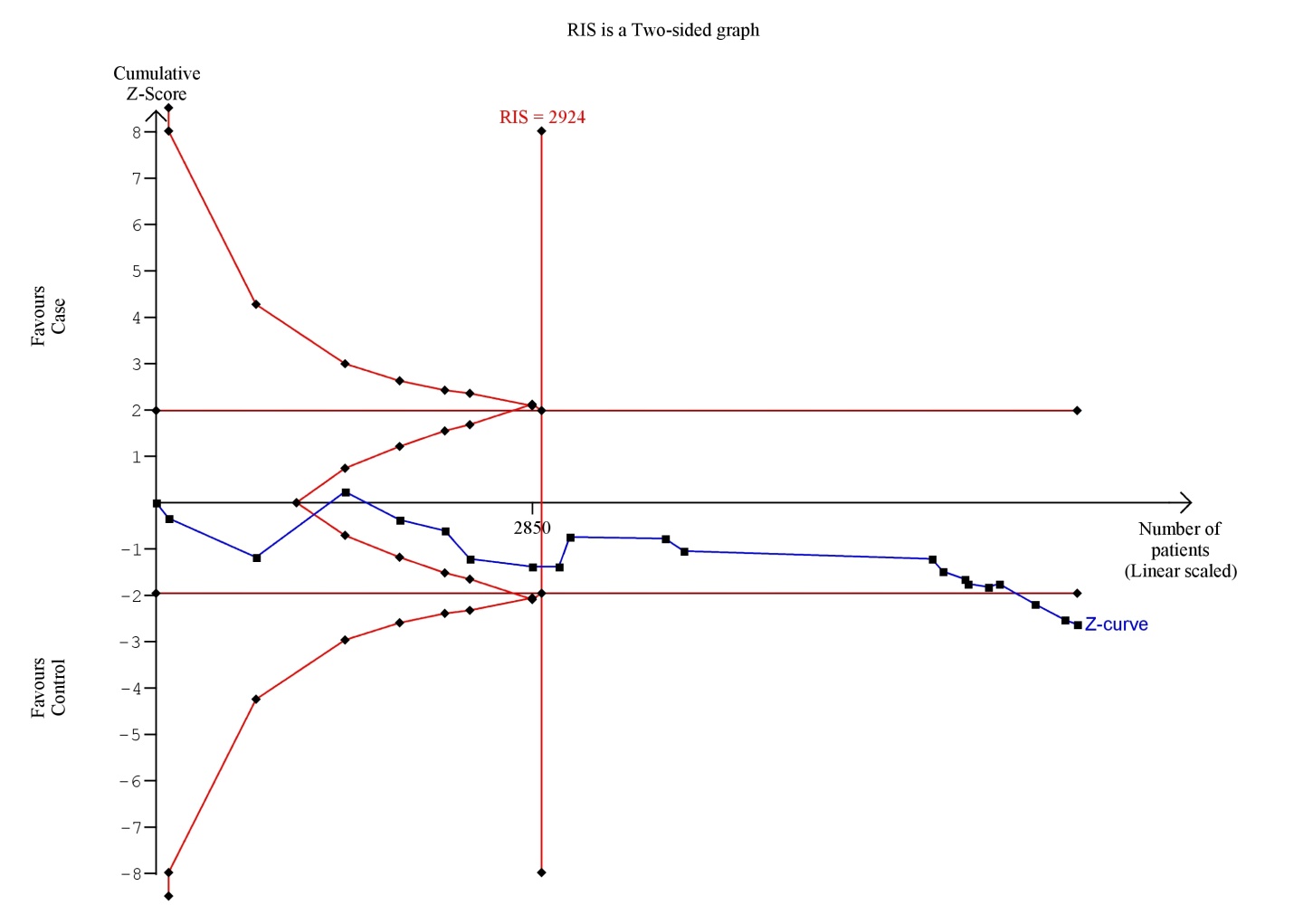


**Figure 2S**: Trial sequential analysis of the association between *ERCC2* *Lys751Gln* polymorphism and the risk of leukemia in homozygous model


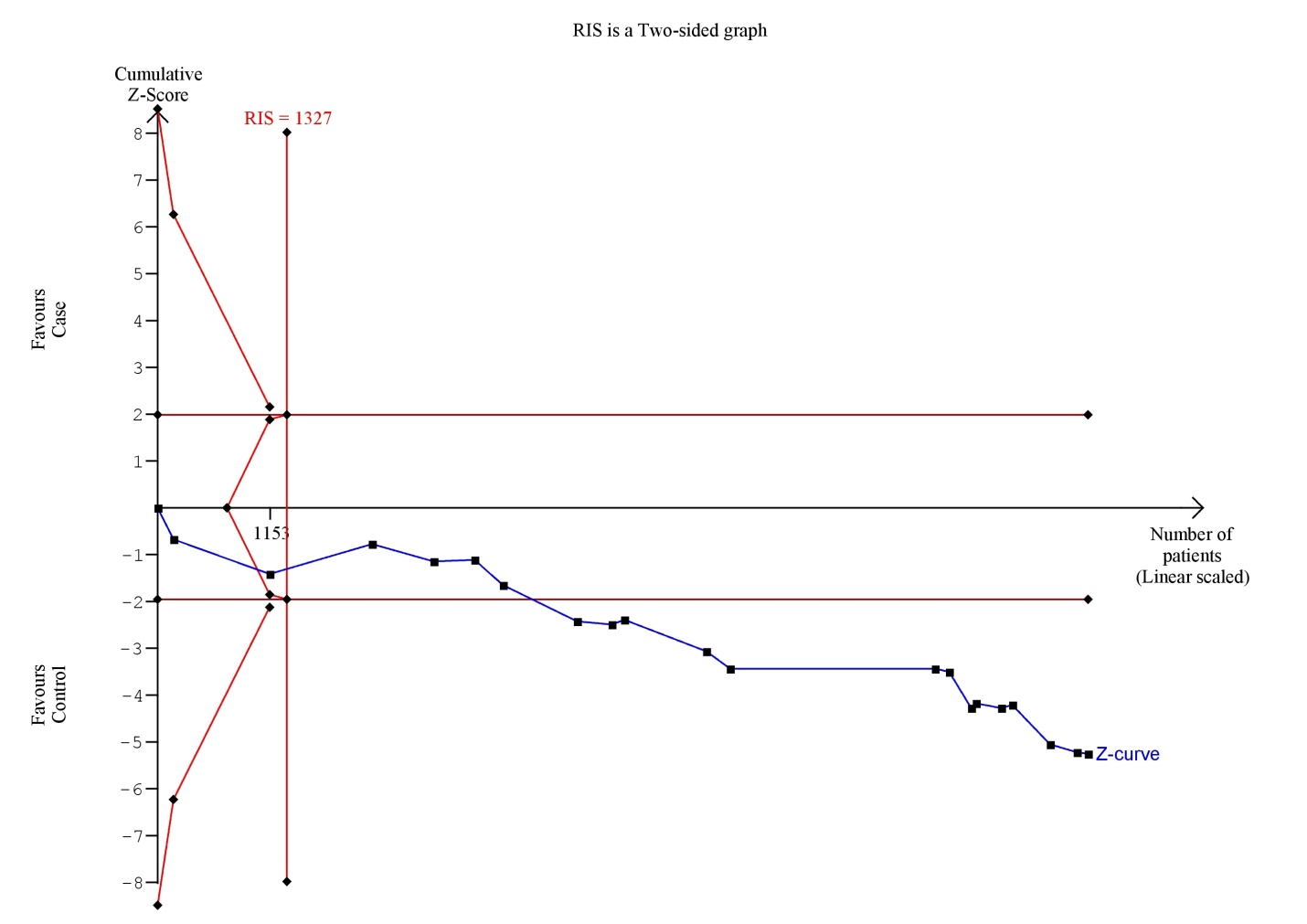


**Figure 3S**: Trial sequential analysis of the association between *ERCC2* *Lys751Gln* polymorphism and the risk of leukemia in heterozygous model


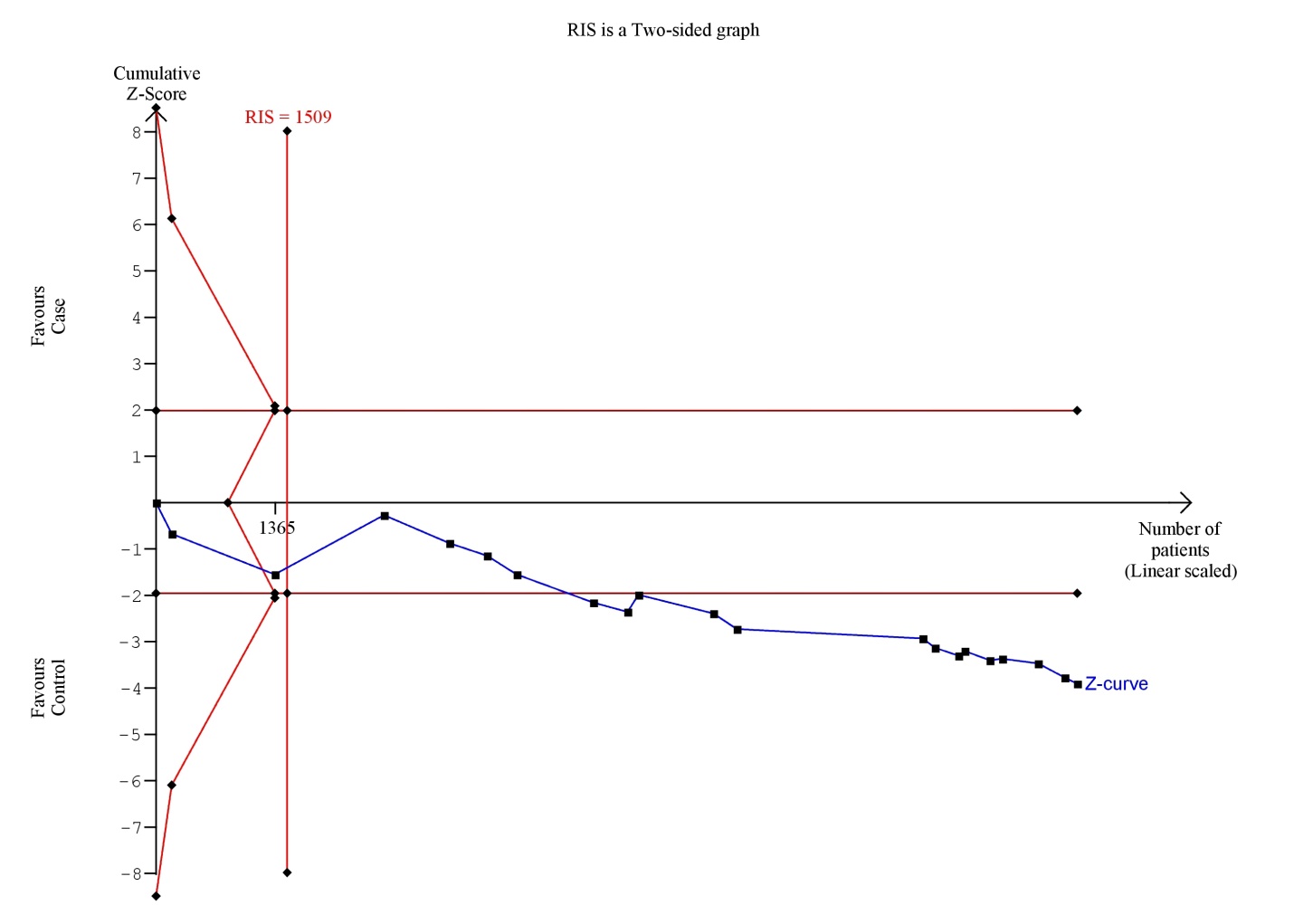


**Figure 4S**: Trial sequential analysis of the association between *ERCC2* *Lys751Gln* polymorphism and the risk of leukemia in dominant model


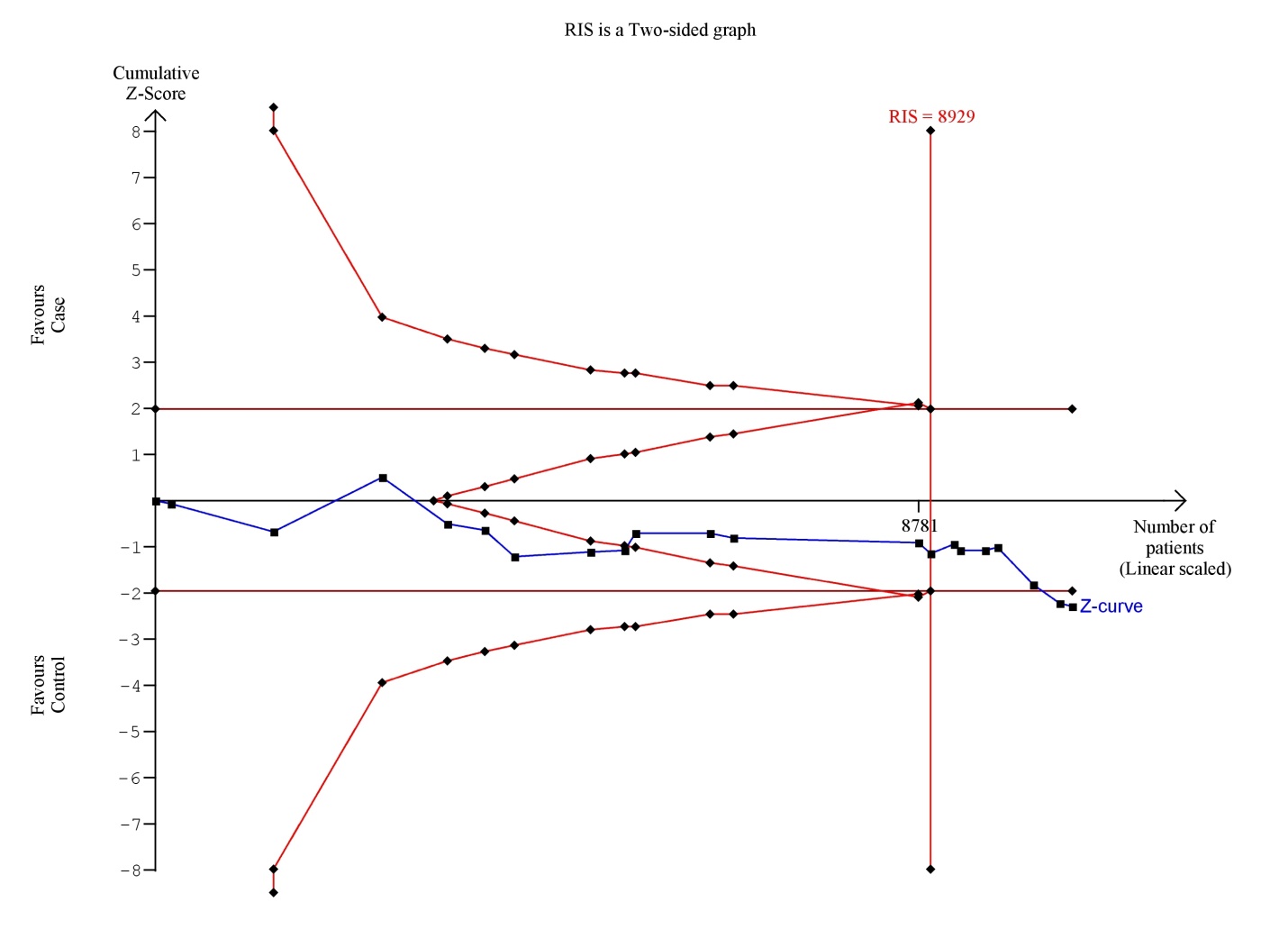


**Figure 5S**: Trial sequential analysis of the association between *ERCC2* *Lys751Gln* polymorphism and the risk of leukemia in recessive model


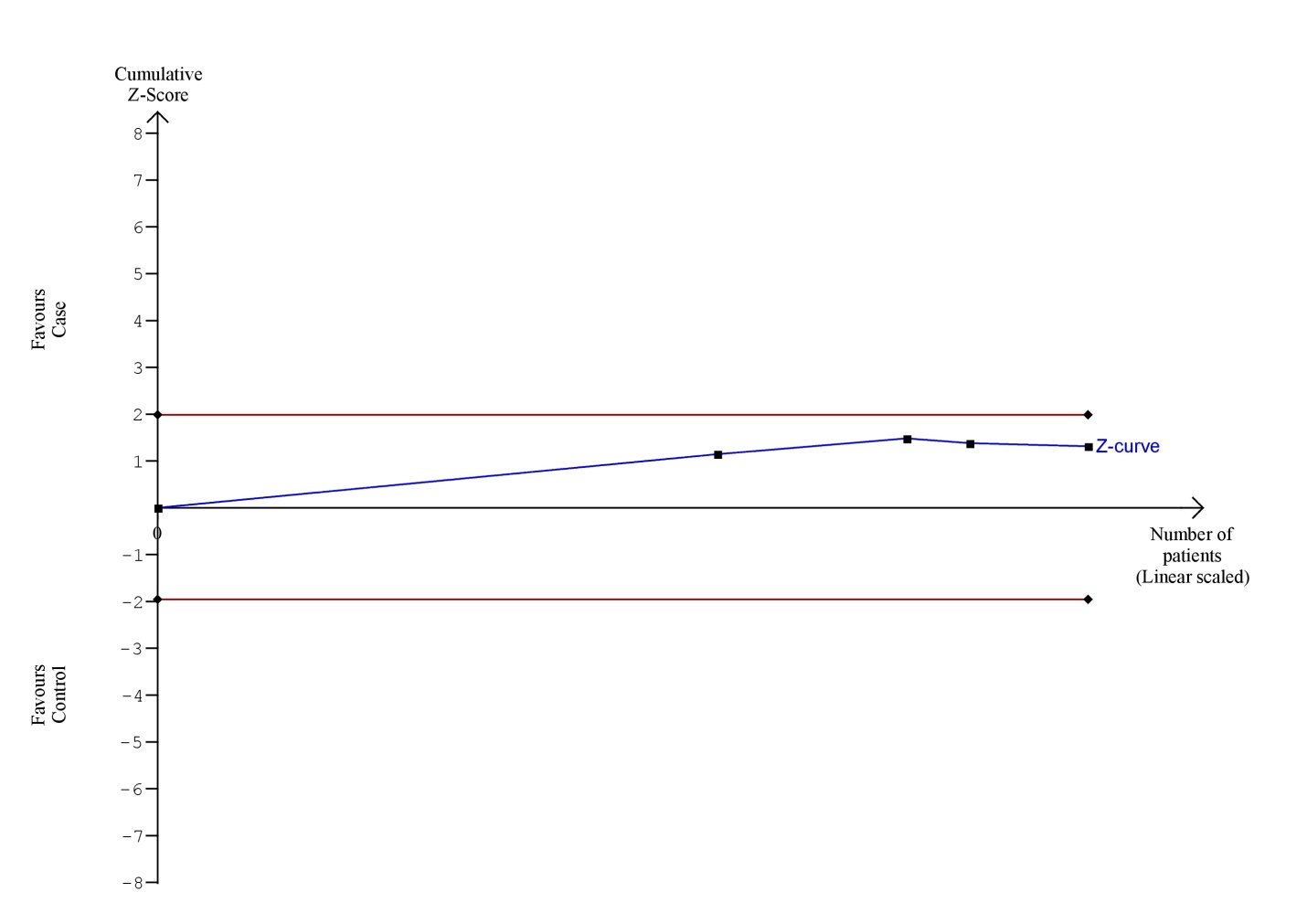


**Figure 6S**: Trial sequential analysis of the association between *ERCC2* Asp312Asn polymorphism and the risk of leukemia in allelic model


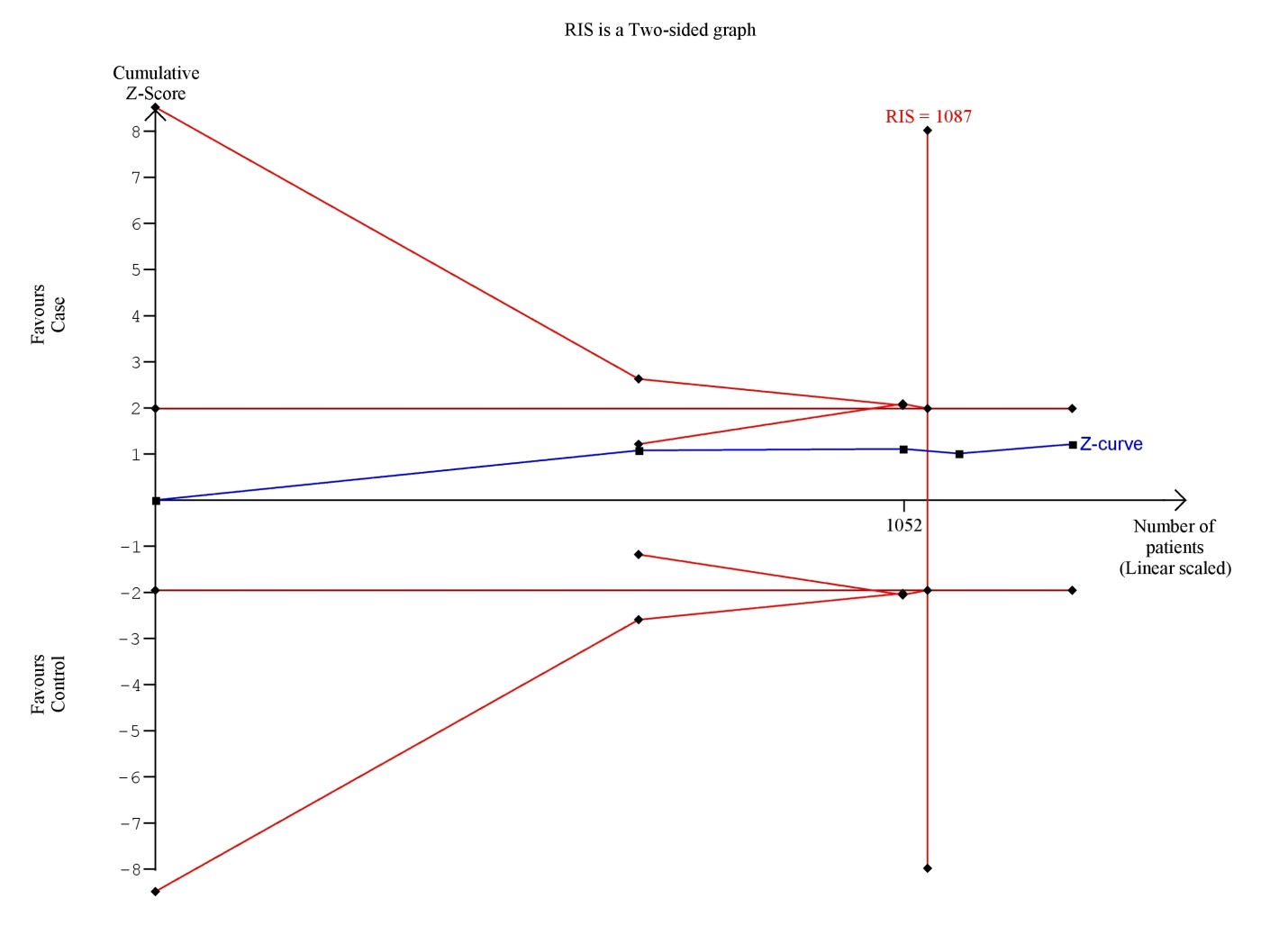


**Figure 7S**: Trial sequential analysis of the association between *ERCC2* Asp312Asn polymorphism and the risk of leukemia in homozygous model


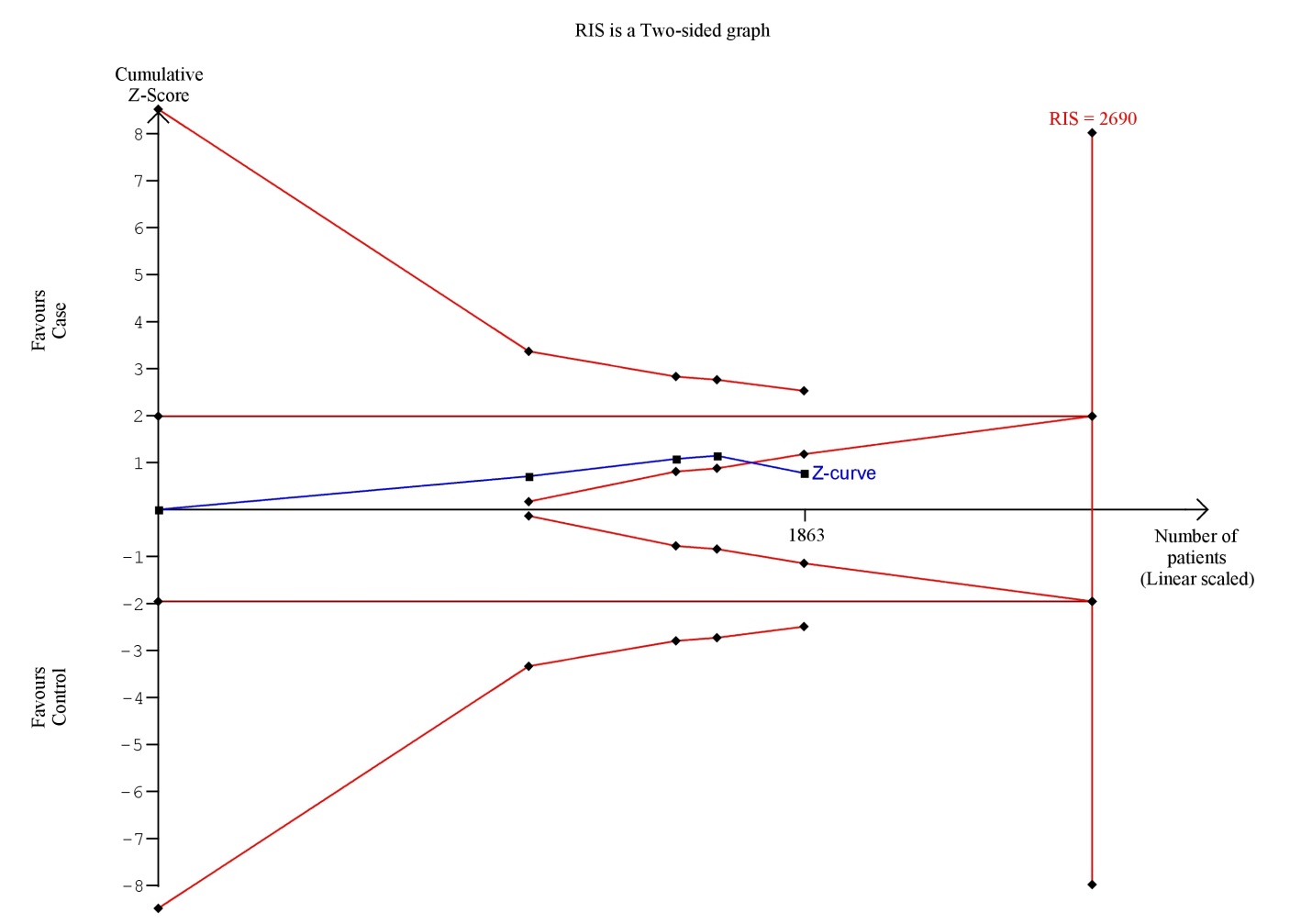


**Figure 8S**: Trial sequential analysis of the association between *ERCC2* Asp312Asn polymorphism and the risk of leukemia in heterozygous model


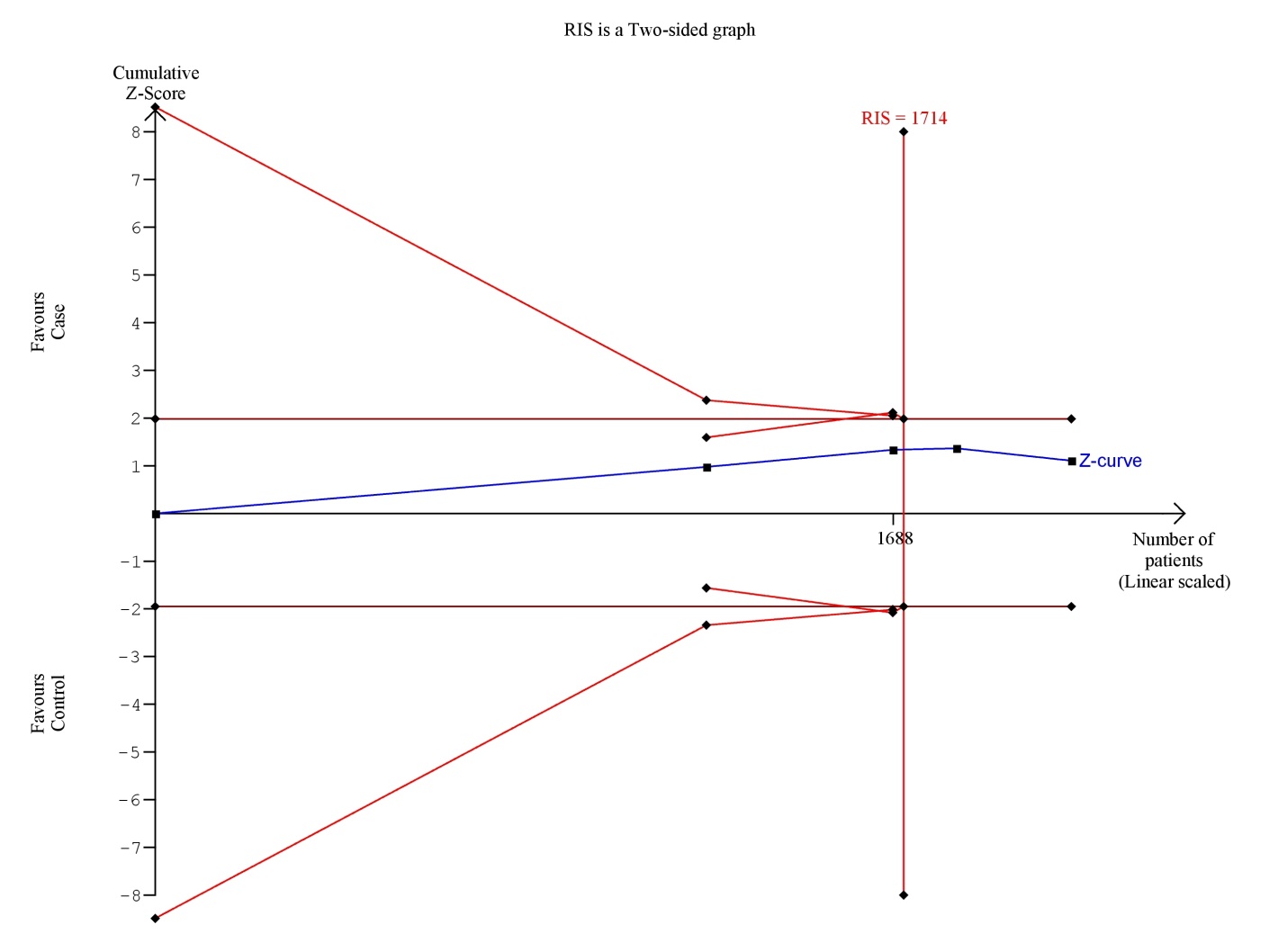


**Figure 9S**: Trial sequential analysis of the association between *ERCC2* Asp312Asn polymorphism and the risk of leukemia in dominant model


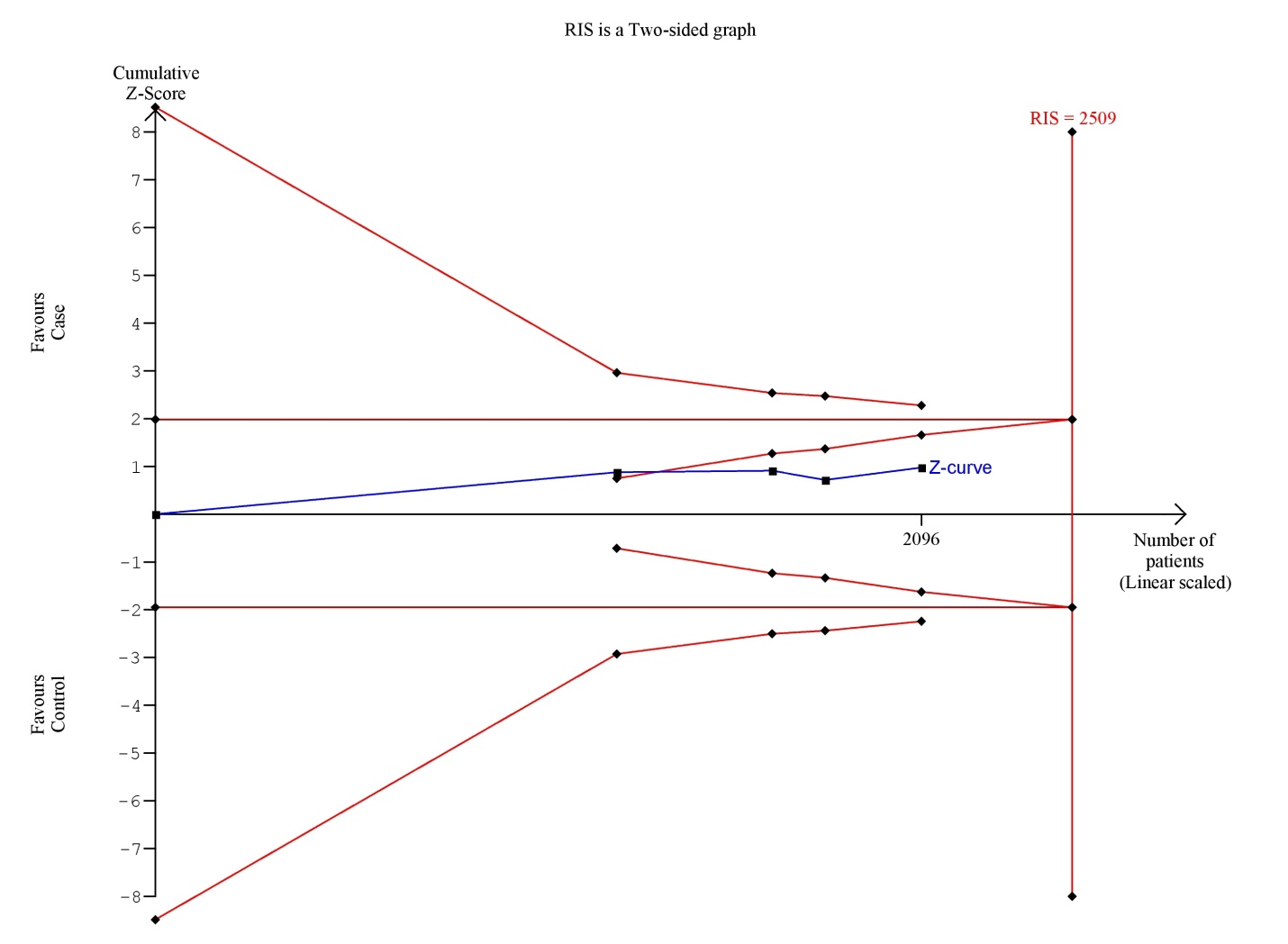


**Figure 10S**: Trial sequential analysis of the association between *ERCC2* Asp312Asn polymorphism and the risk of leukemia in recessive model


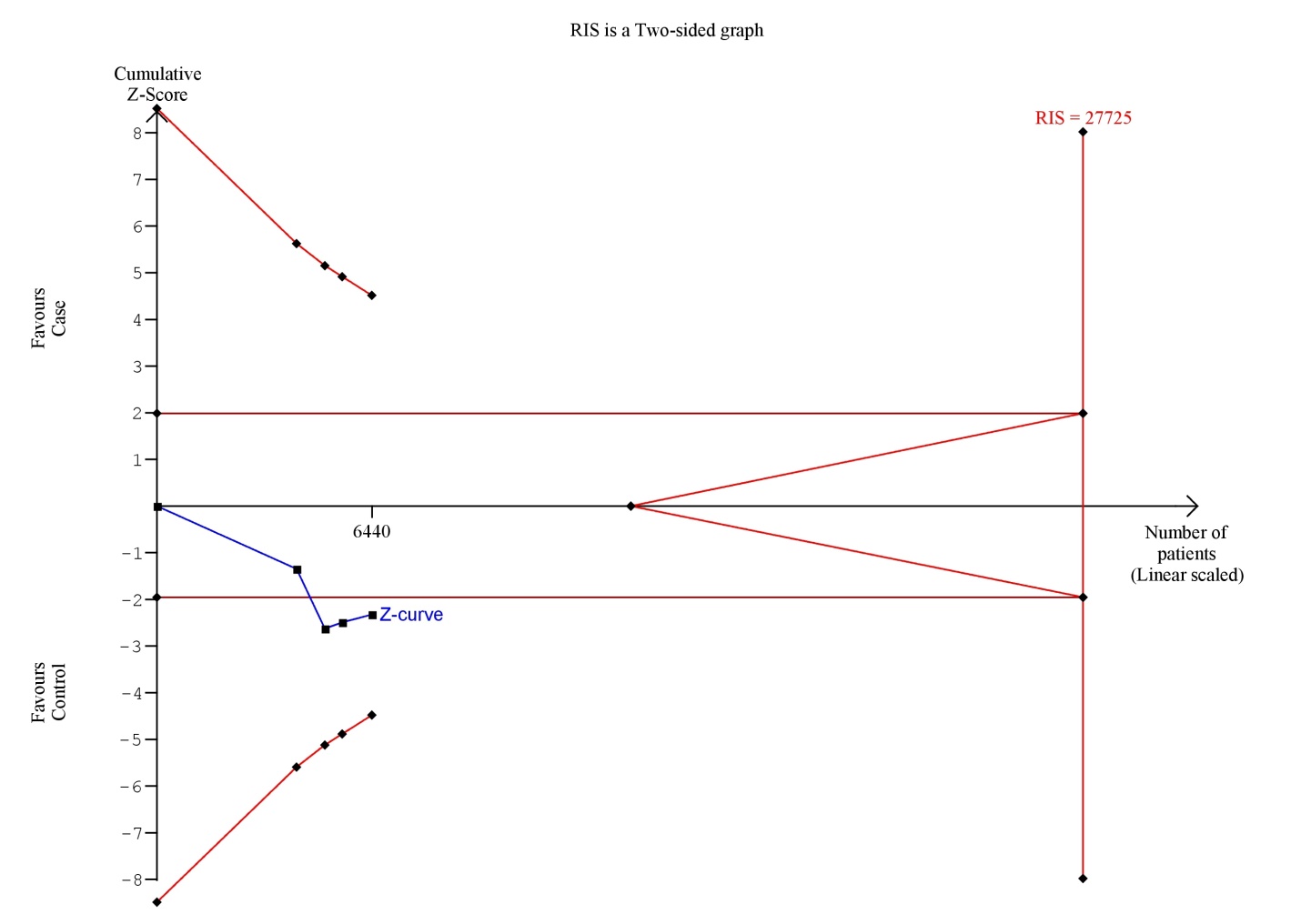


**Figure 11S**: Trial sequential analysis of the association between *XPC* *Lys939Gln* polymorphism and the risk of leukemia in allelic model


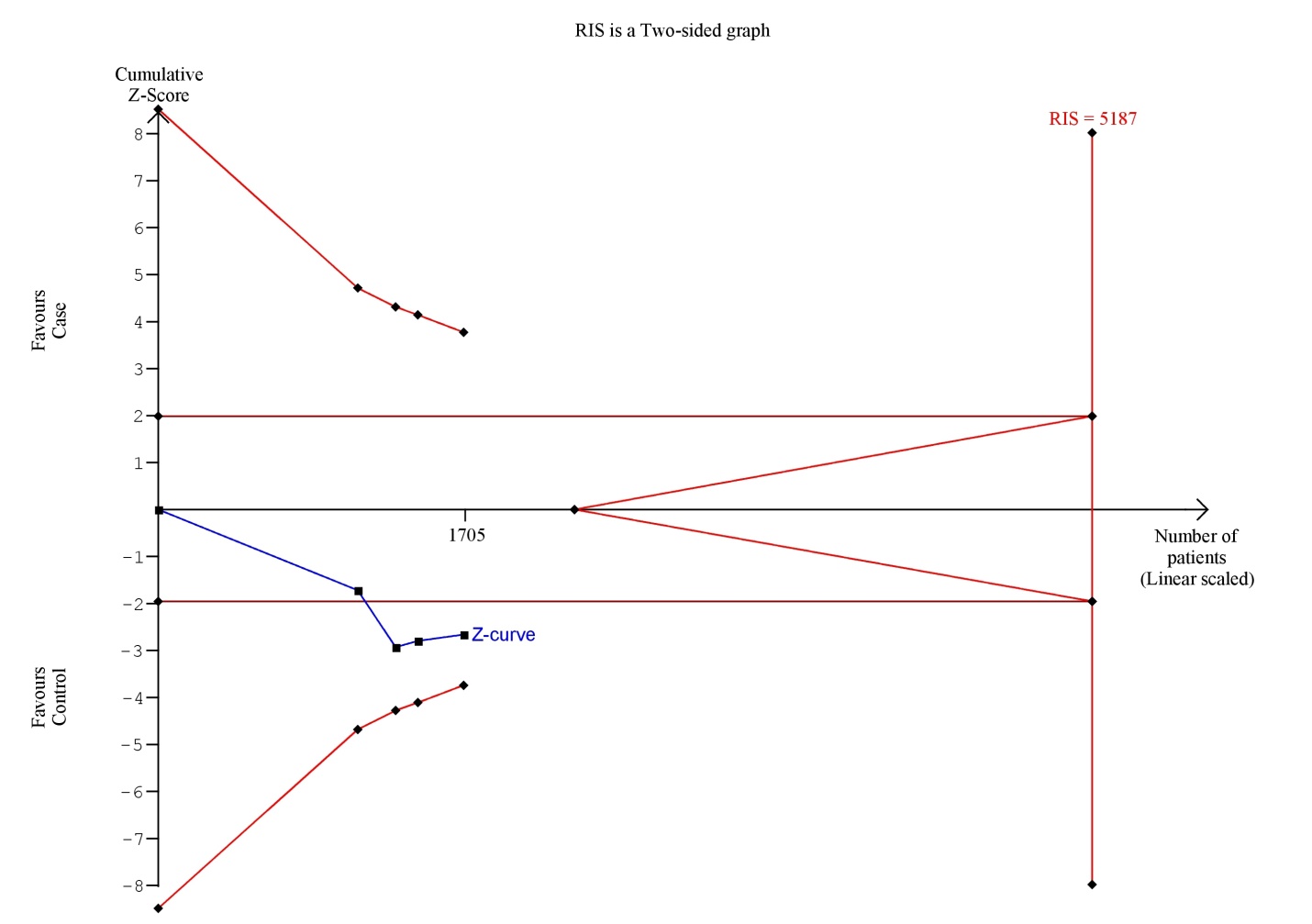


**Figure 12S**: Trial sequential analysis of the association between *XPC* *Lys939Gln* polymorphism and the risk of leukemia in homozygous model


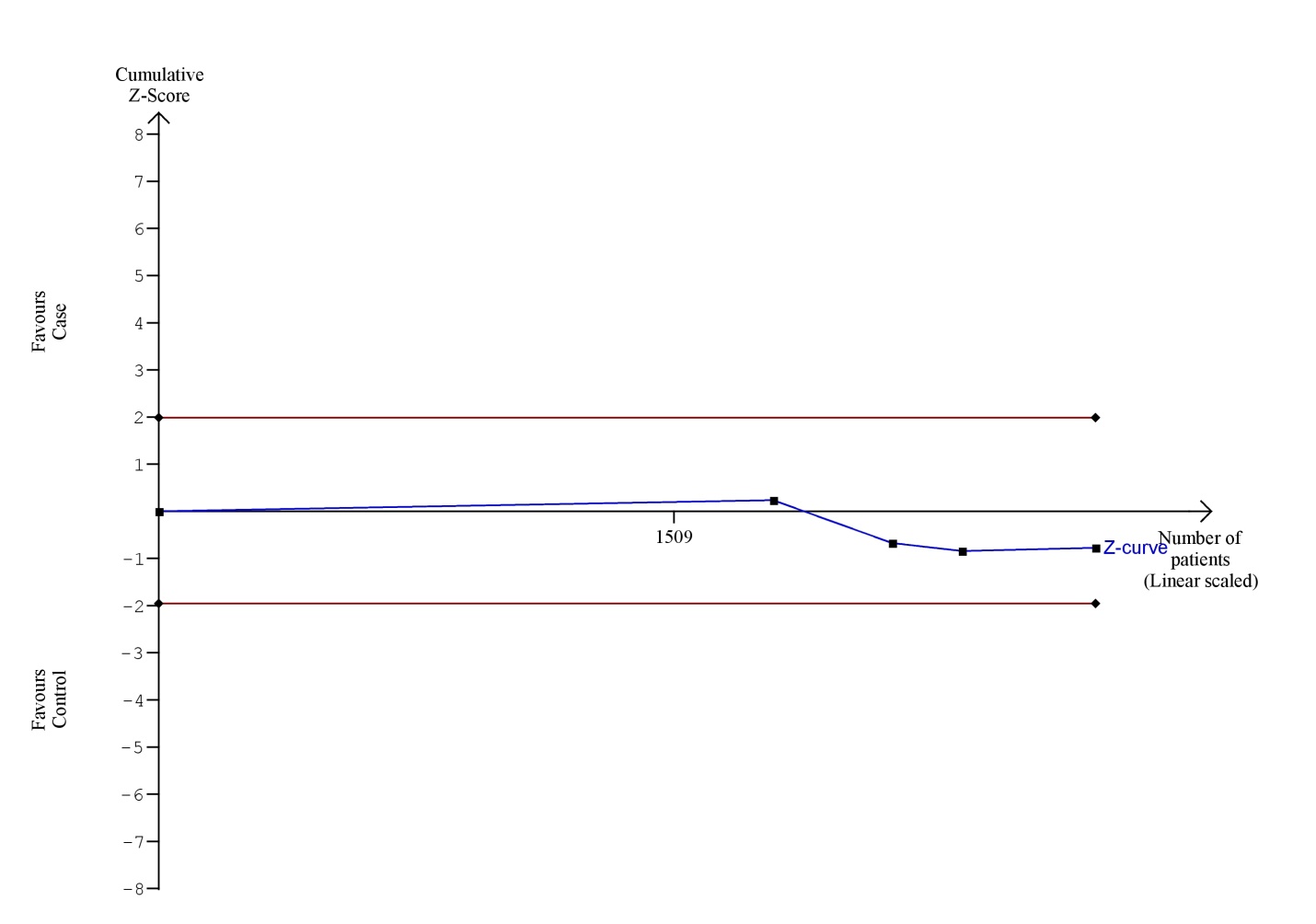


**Figure 13S**: Trial sequential analysis of the association between *XPC* *Lys939Gln* polymorphism and the risk of leukemia in heterozygous model


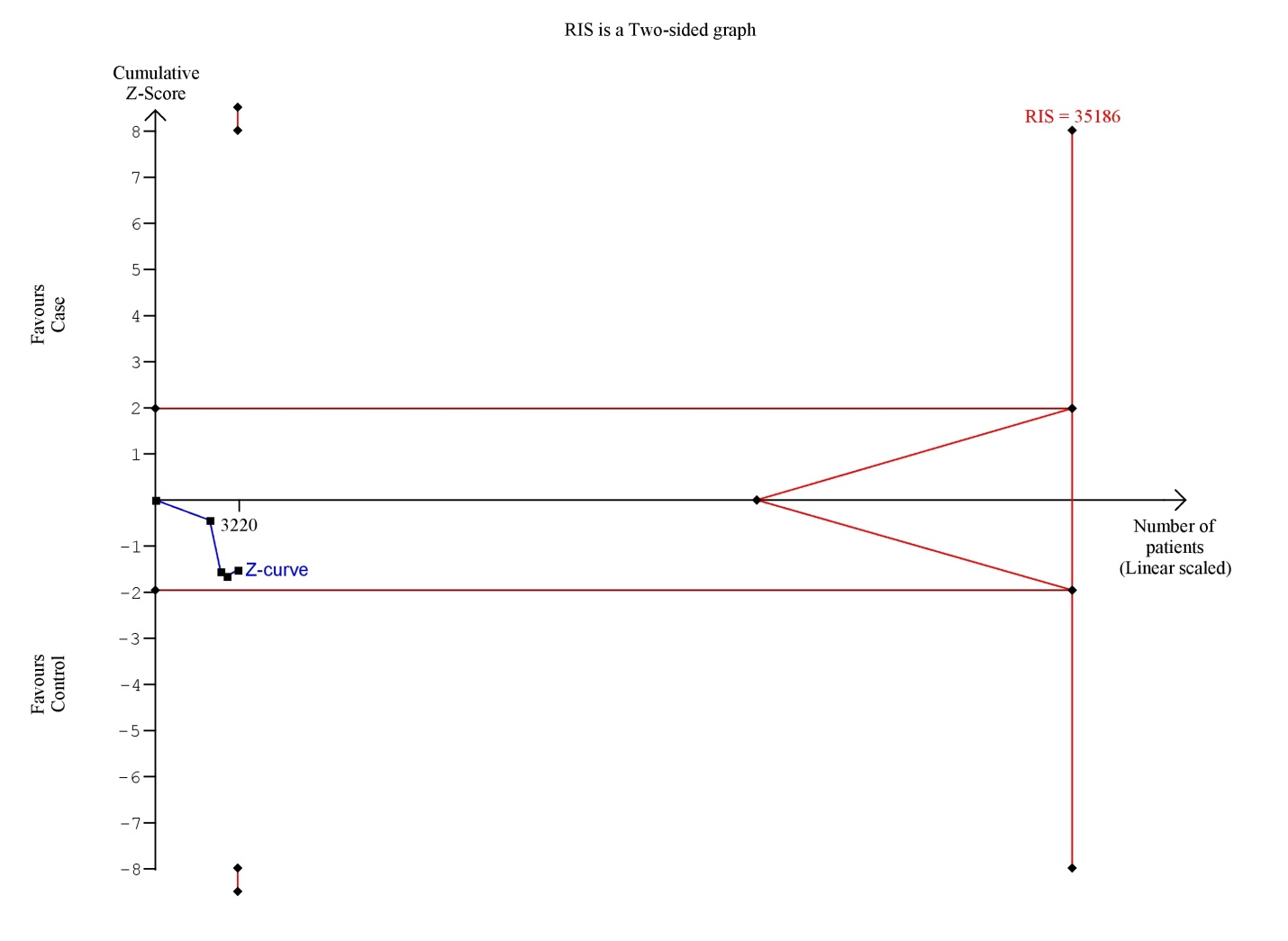


**Figure 14S**: Trial sequential analysis of the association between *XPC* *Lys939Gln* polymorphism and the risk of leukemia in dominant model


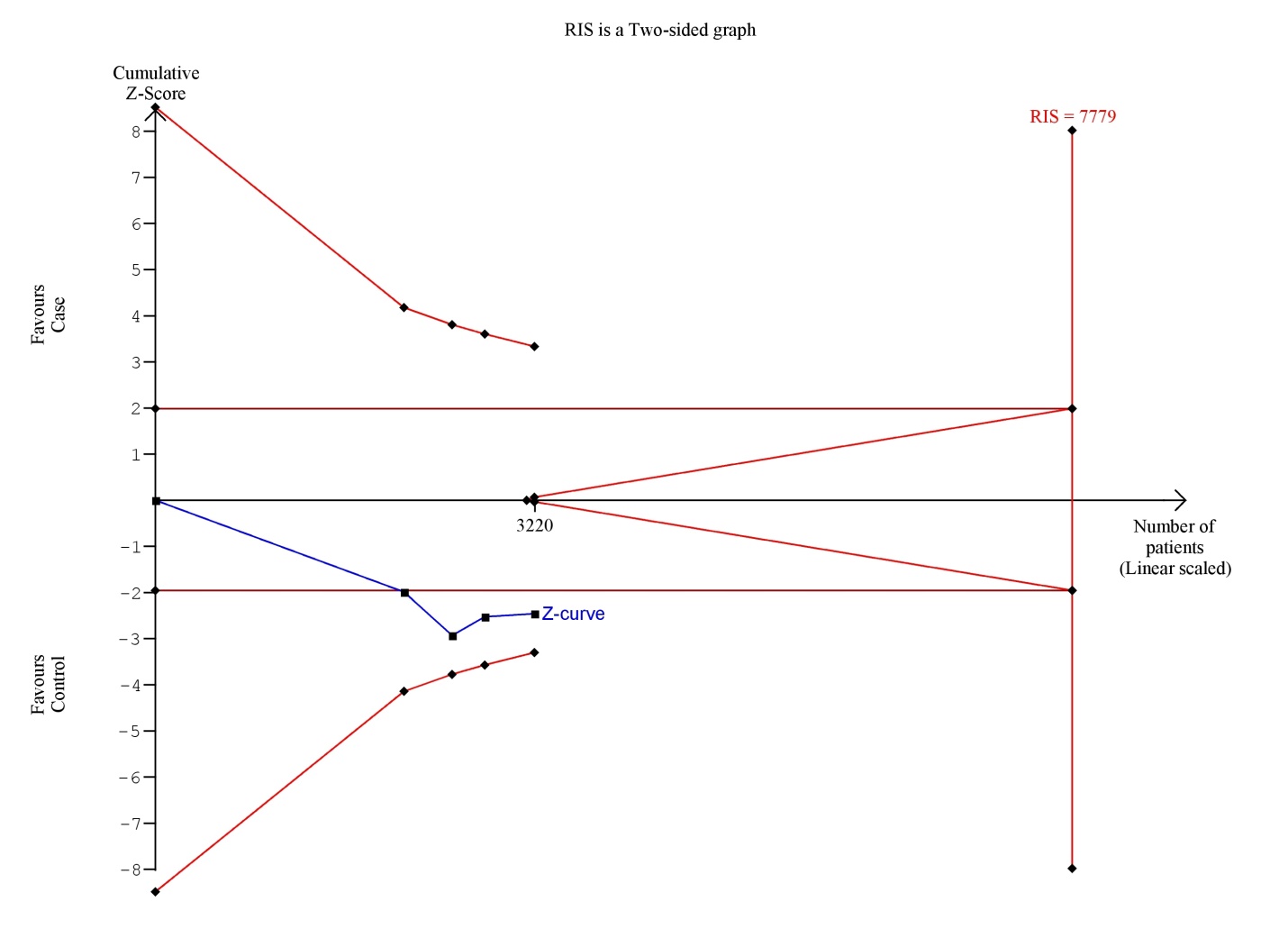


**Figure 15S**: Trial sequential analysis of the association between *XPC* *Lys939Gln* polymorphism and the risk of leukemia in recessive model


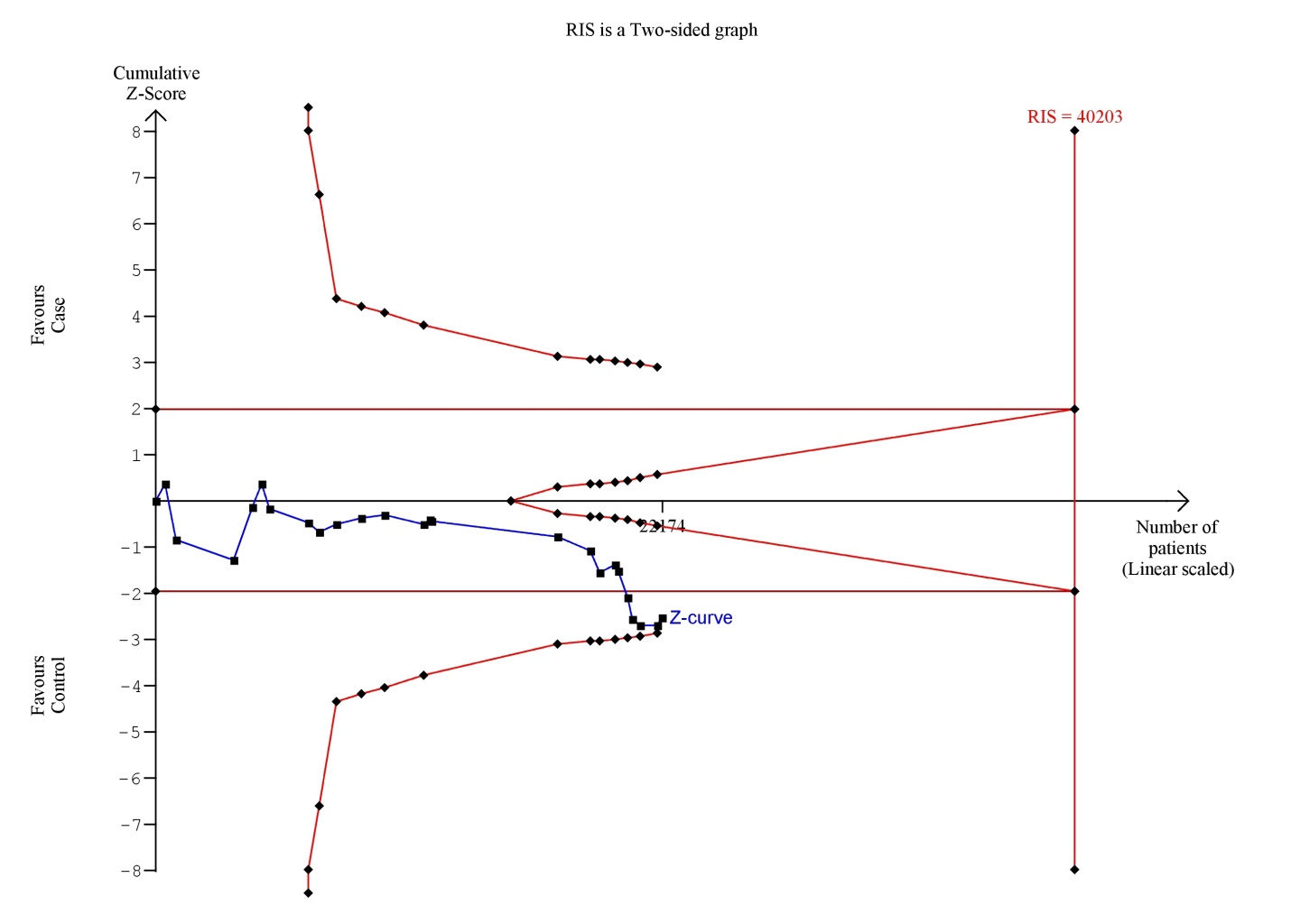


**Figure 16S**: Trial sequential analysis of the association between *XRCC1 Arg194Trp (rs1799782)* polymorphism and the risk of leukemia in allelic model


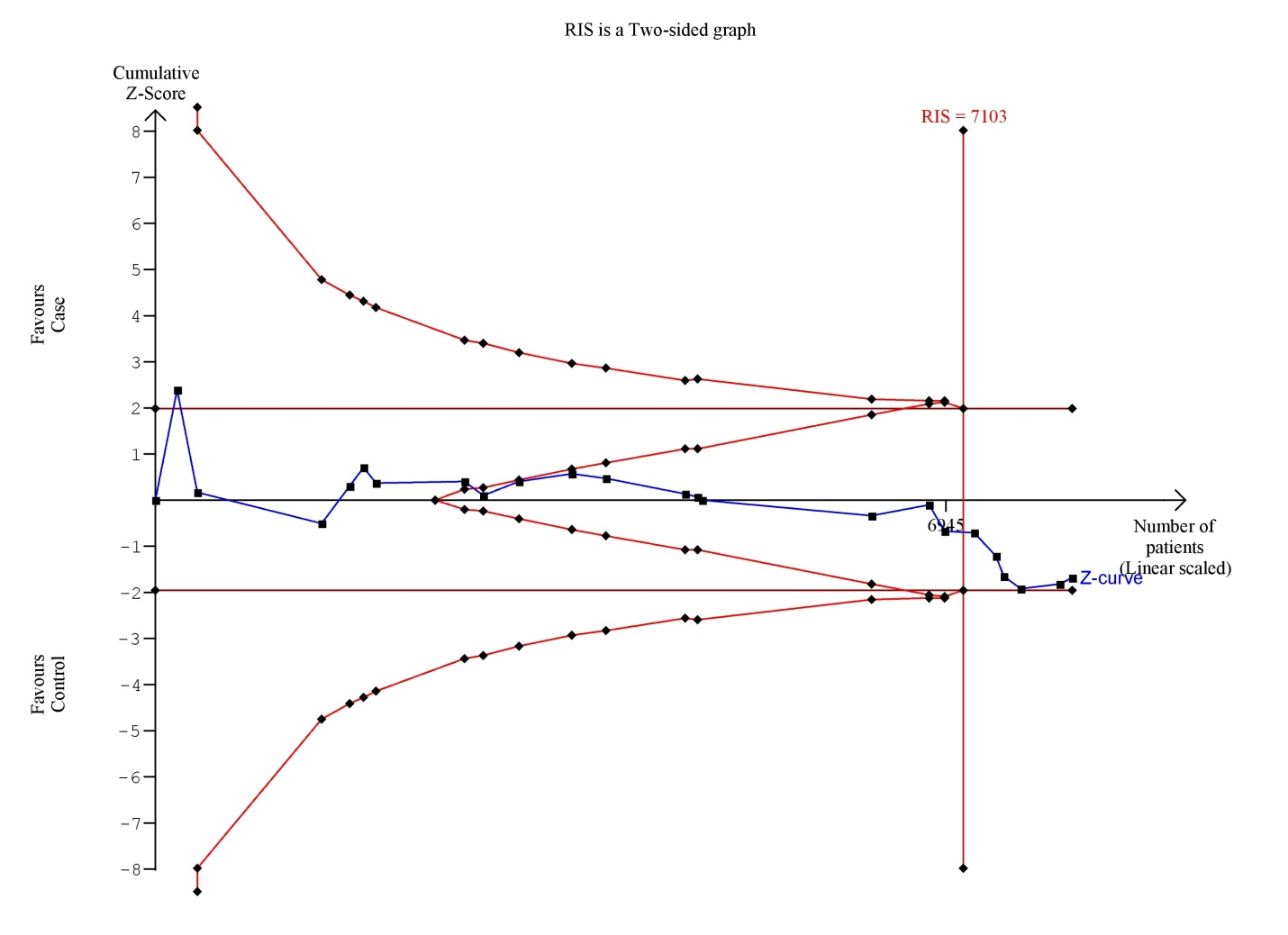


**Figure 17S**: Trial sequential analysis of the association between *XRCC1 Arg194Trp (rs1799782)* polymorphism and the risk of leukemia in homozygous model


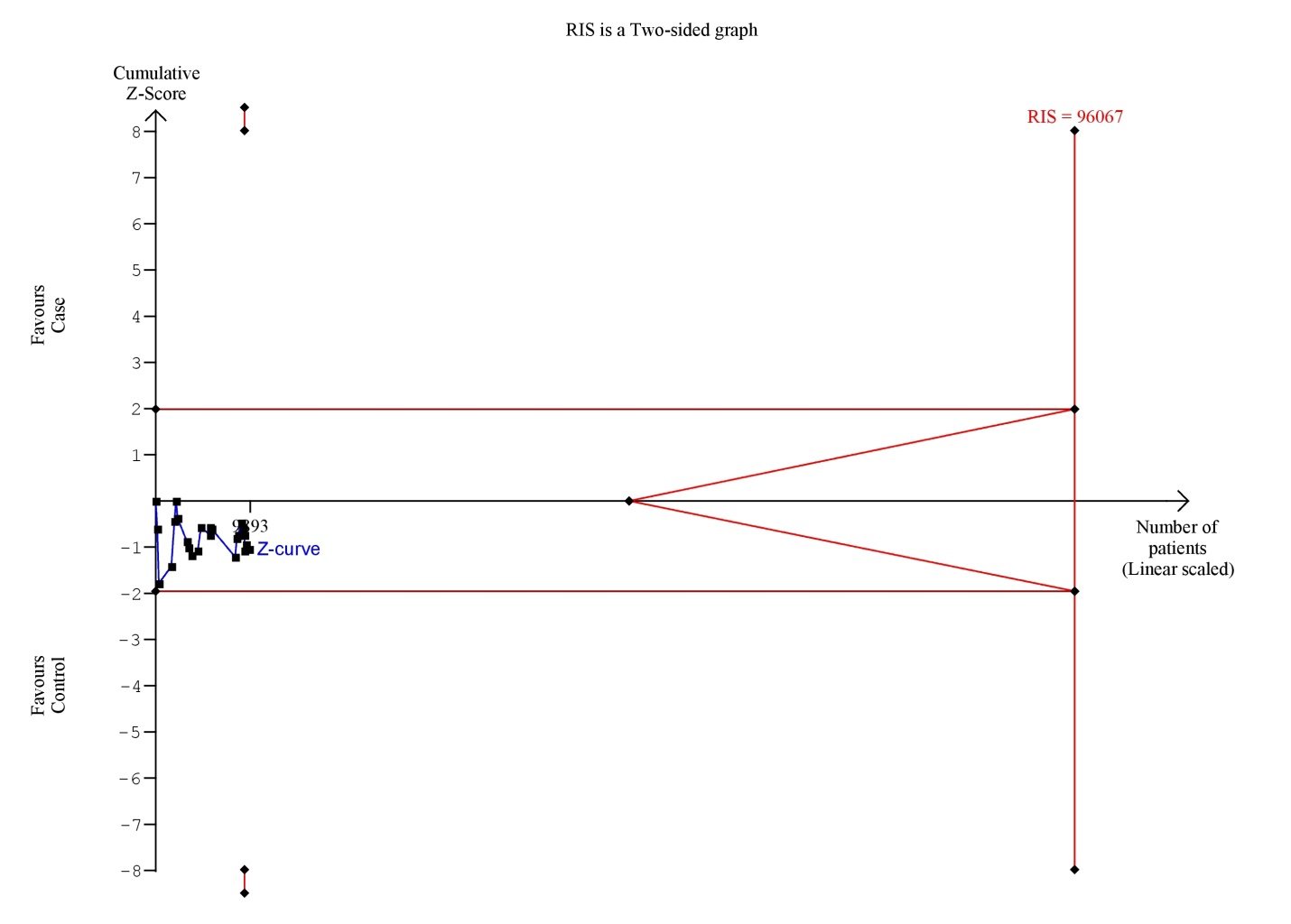


**Figure 18S**: Trial sequential analysis of the association between *XRCC1 Arg194Trp (rs1799782)* polymorphism and the risk of leukemia in heterozygous model


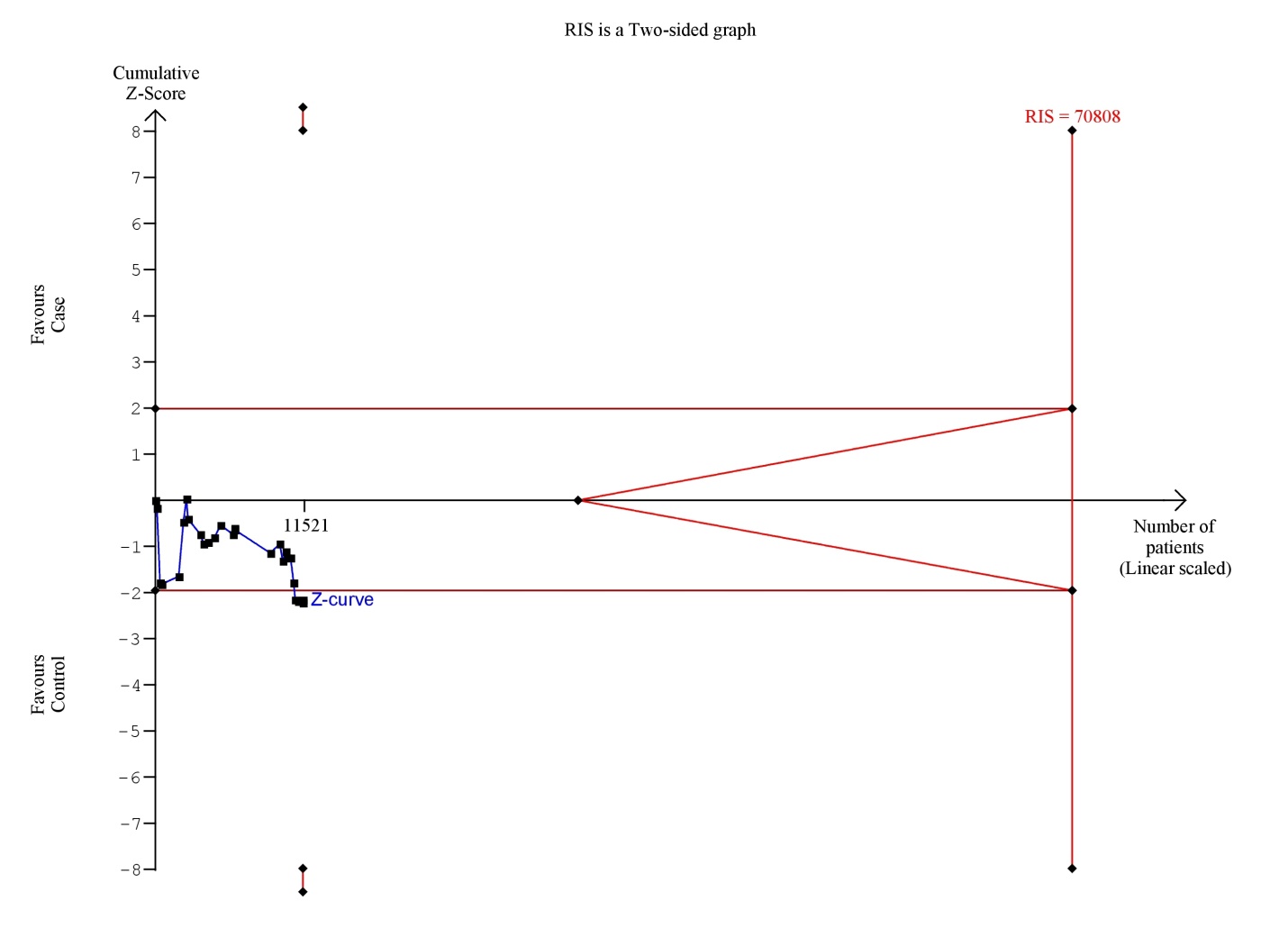


**Figure 19S**: Trial sequential analysis of the association between *XRCC1 Arg194Trp (rs1799782)* polymorphism and the risk of leukemia in dominant model


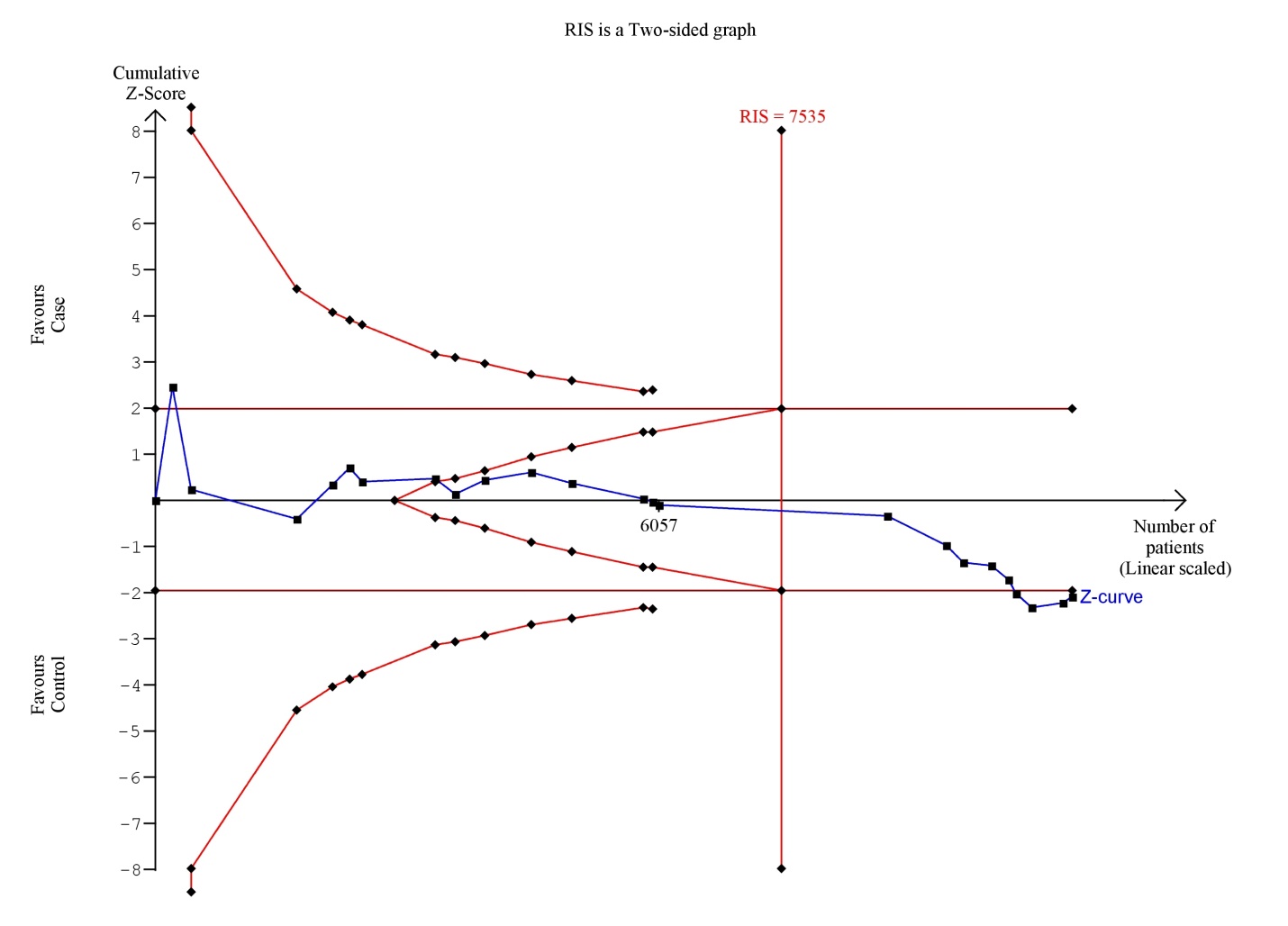


**Figure 20S**: Trial sequential analysis of the association between *XRCC1 Arg194Trp (rs1799782)* polymorphism and the risk of leukemia in recessive model


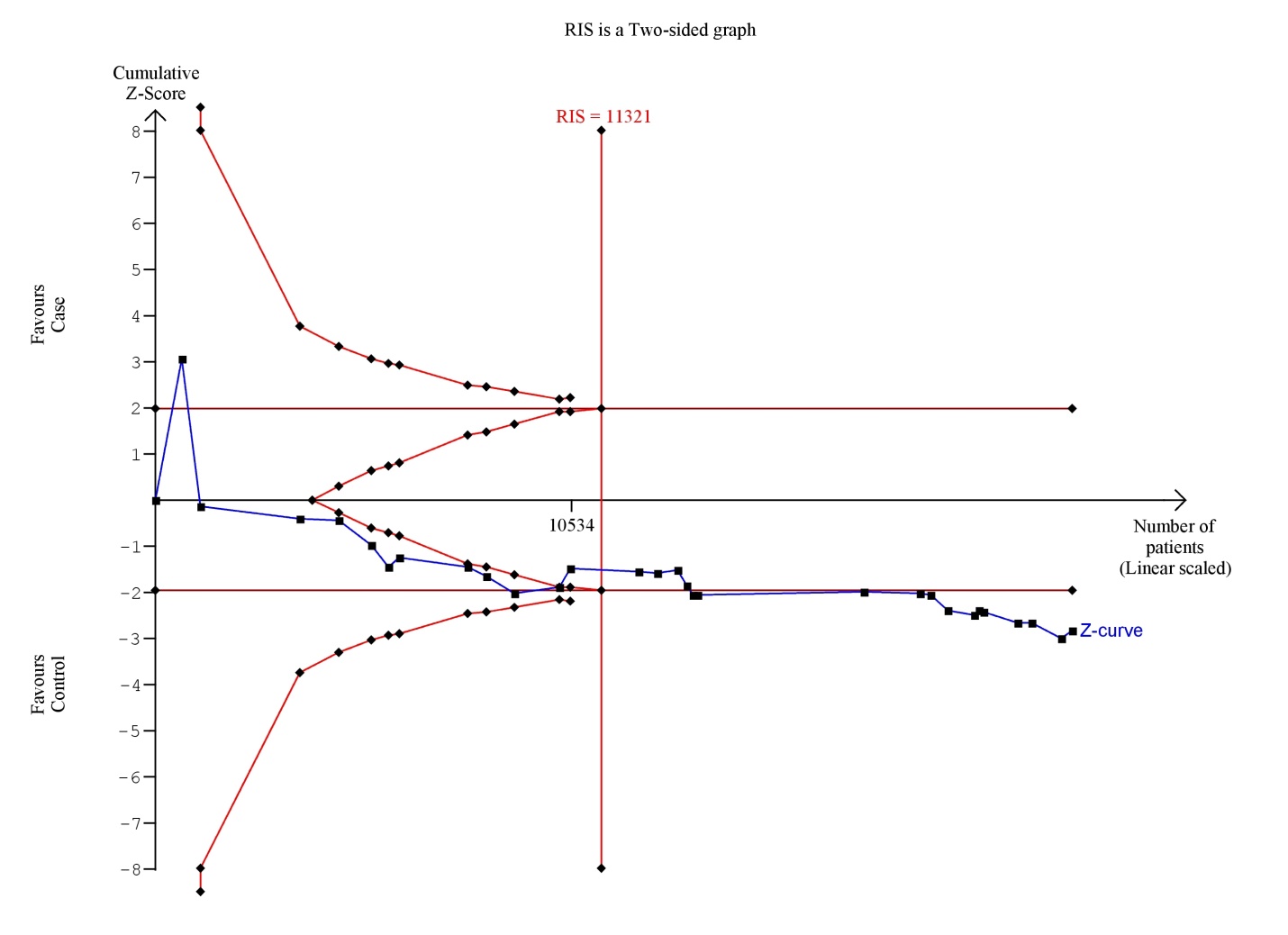


**Figure 21S**: Trial sequential analysis of the association between *XRCC1 Arg399Gln (rs25487)* polymorphism and the risk of leukemia in allelic model


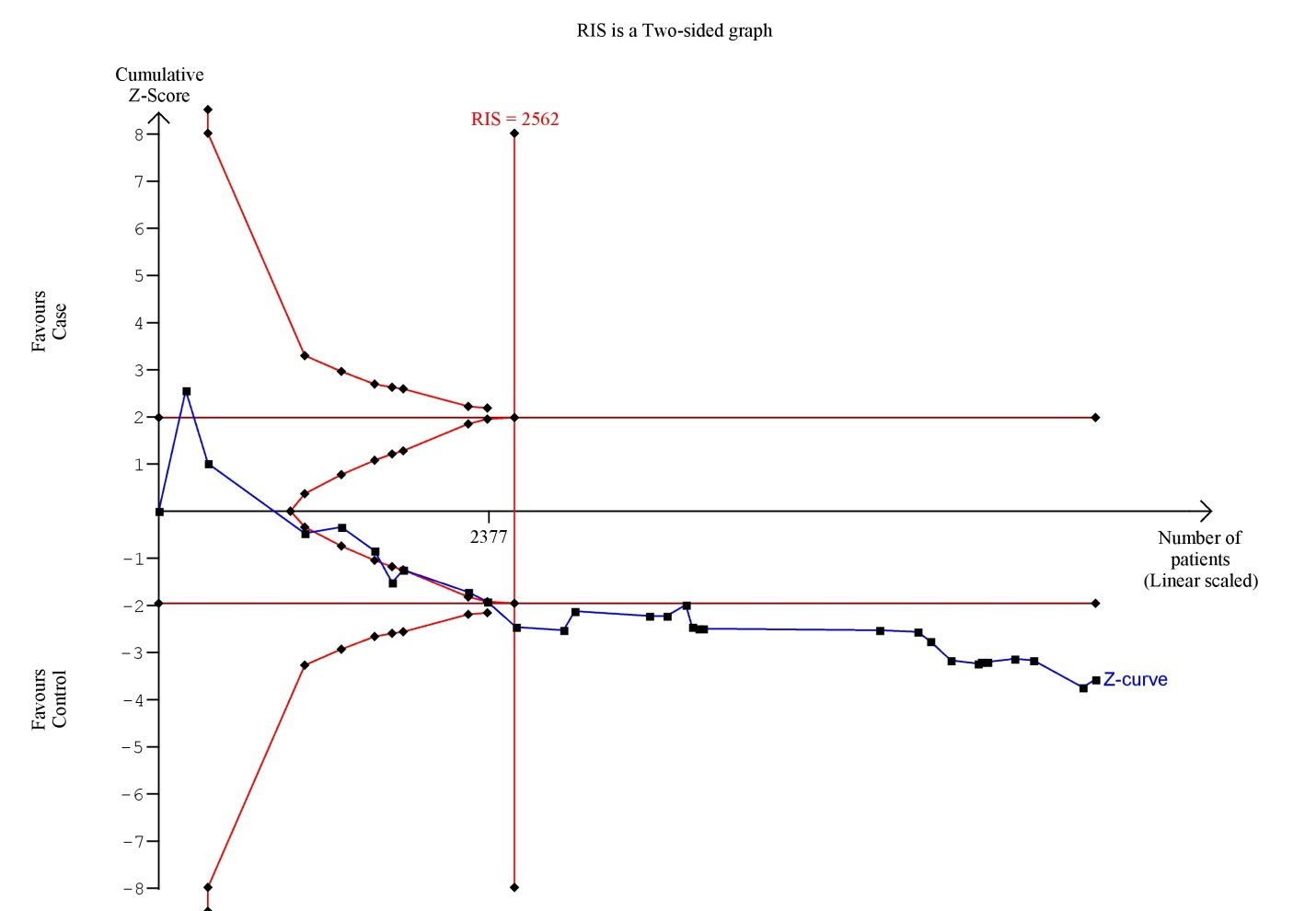


**Figure 22S**: Trial sequential analysis of the association between *XRCC1 Arg399Gln (rs25487)* polymorphism and the risk of leukemia in homozygous model


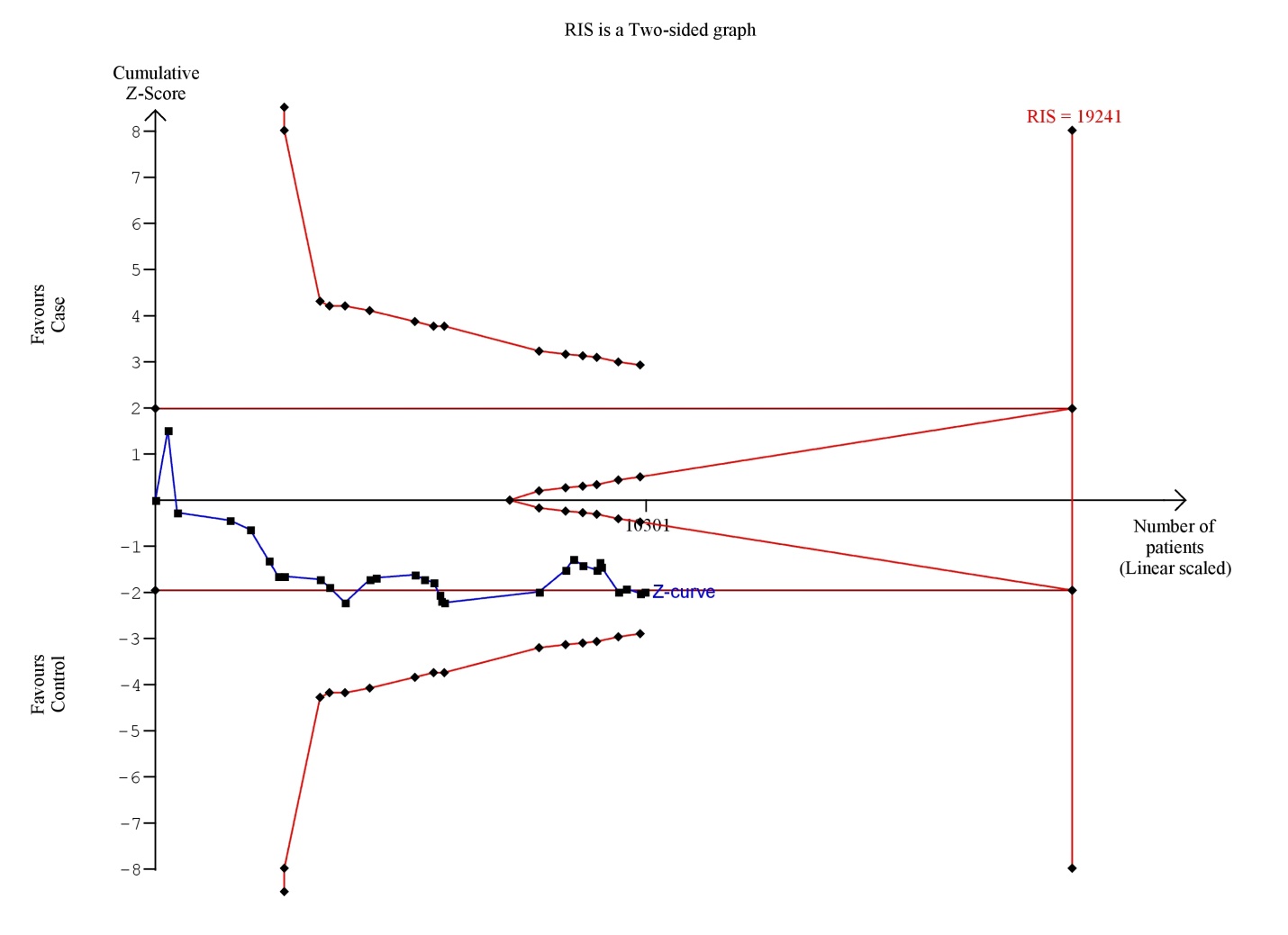


**Figure 23S**: Trial sequential analysis of the association between *XRCC1 Arg399Gln (rs25487)* polymorphism and the risk of leukemia in heterozygous model


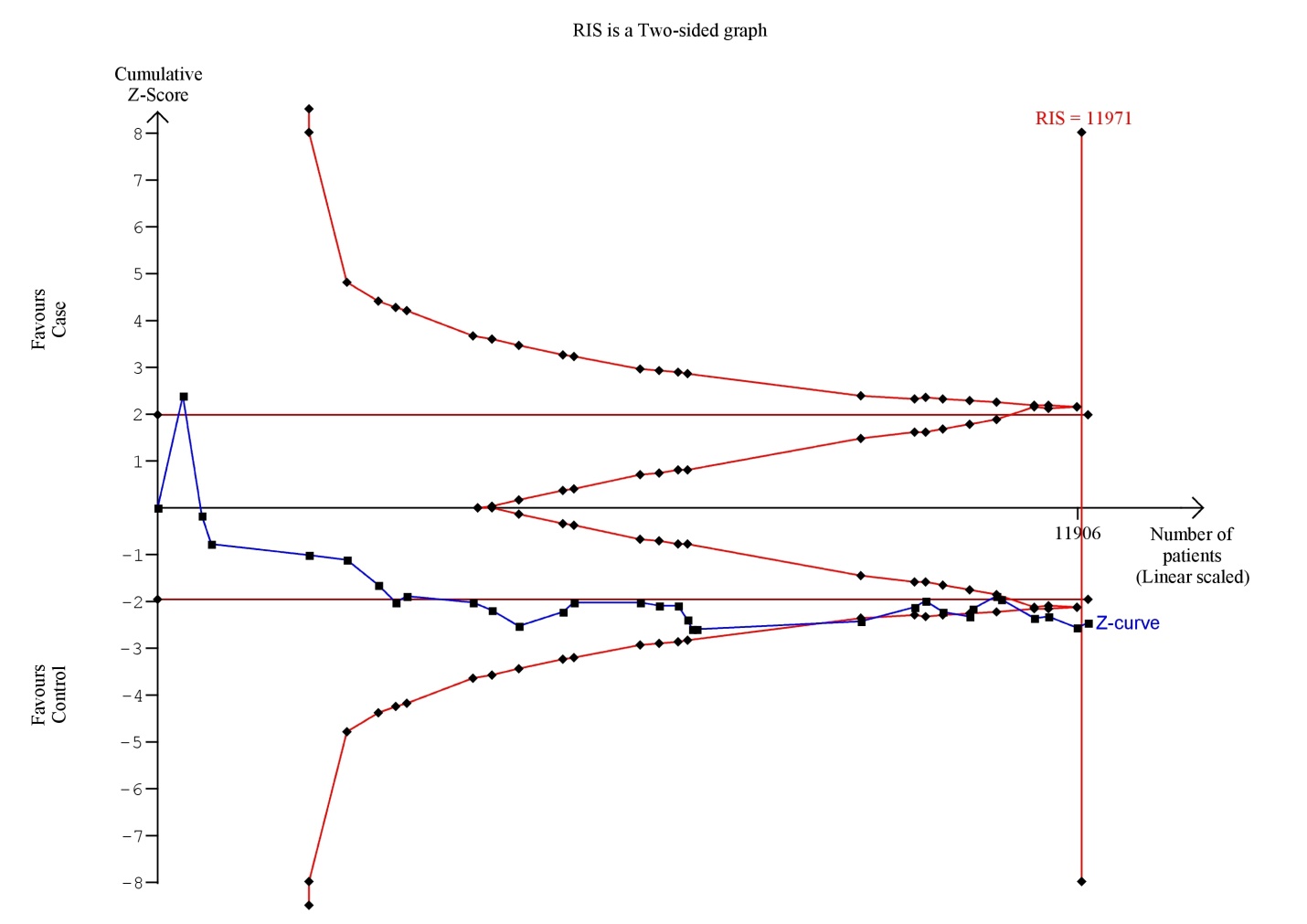


**Figure 24S**: Trial sequential analysis of the association between *XRCC1 Arg399Gln (rs25487)* polymorphism and the risk of leukemia in dominant model


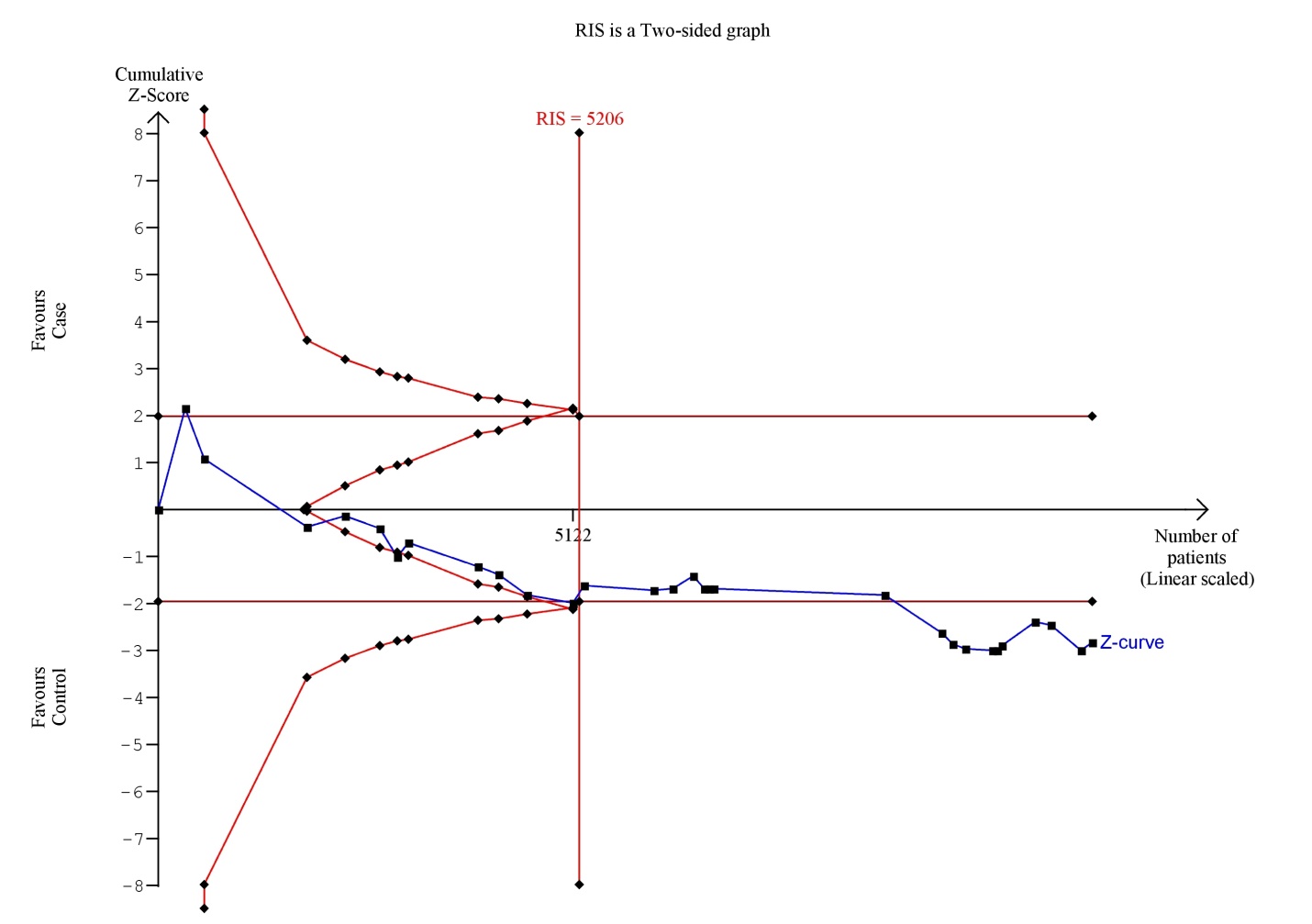


**Figure 25S**: Trial sequential analysis of the association between *XRCC1 Arg399Gln (rs25487)* polymorphism and the risk of leukemia in recessive model


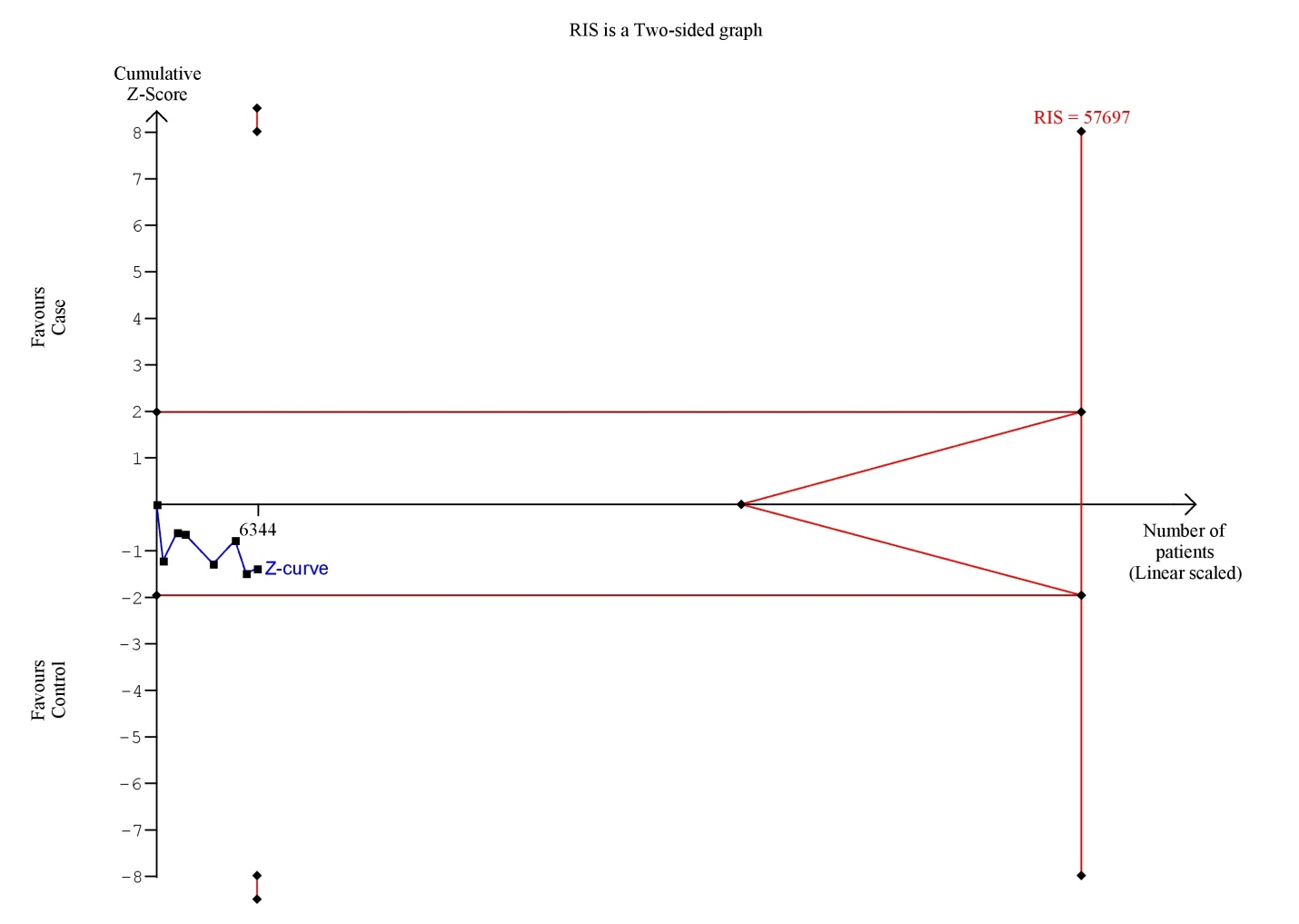


**Figure 26S**: Trial sequential analysis of the association between *XRCC1 Arg280His (rs25489)* polymorphism and the risk of leukemia in allelic model


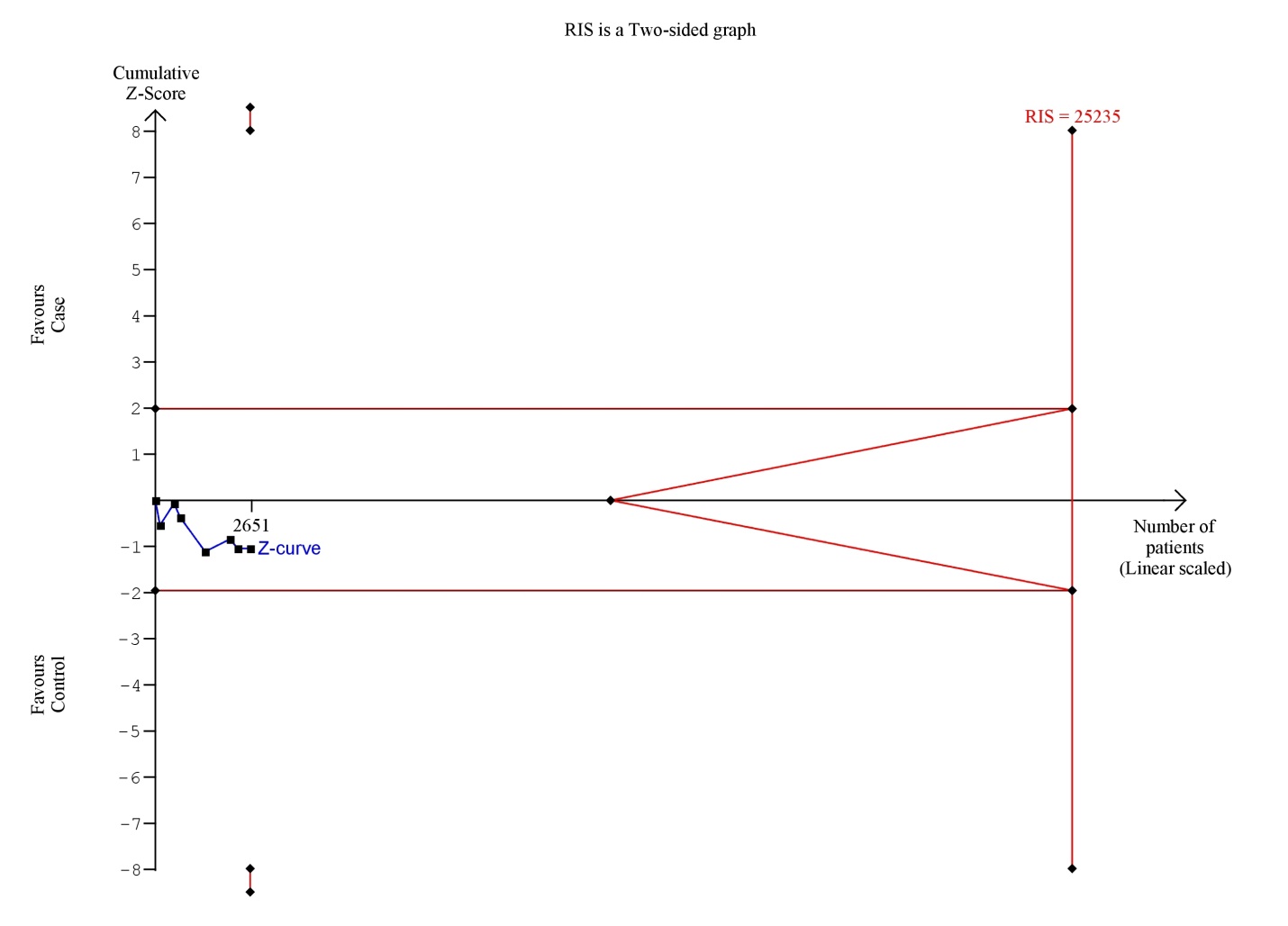


**Figure 27S**: Trial sequential analysis of the association between *XRCC1 Arg280His (rs25489)* polymorphism and the risk of leukemia in homozygous model


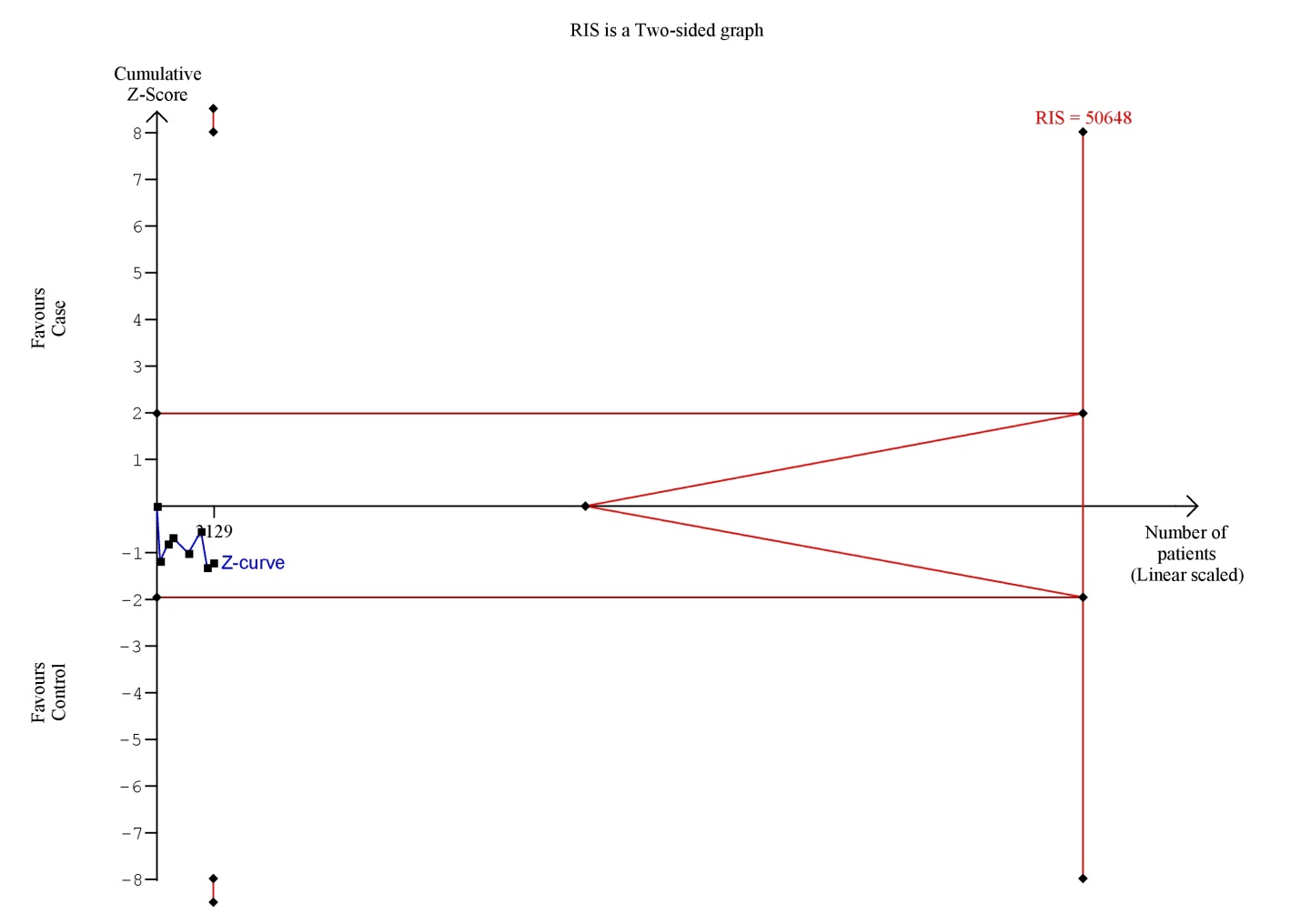


**Figure 28S**: Trial sequential analysis of the association between *XRCC1 Arg280His (rs25489)* polymorphism and the risk of leukemia in heterozygous model


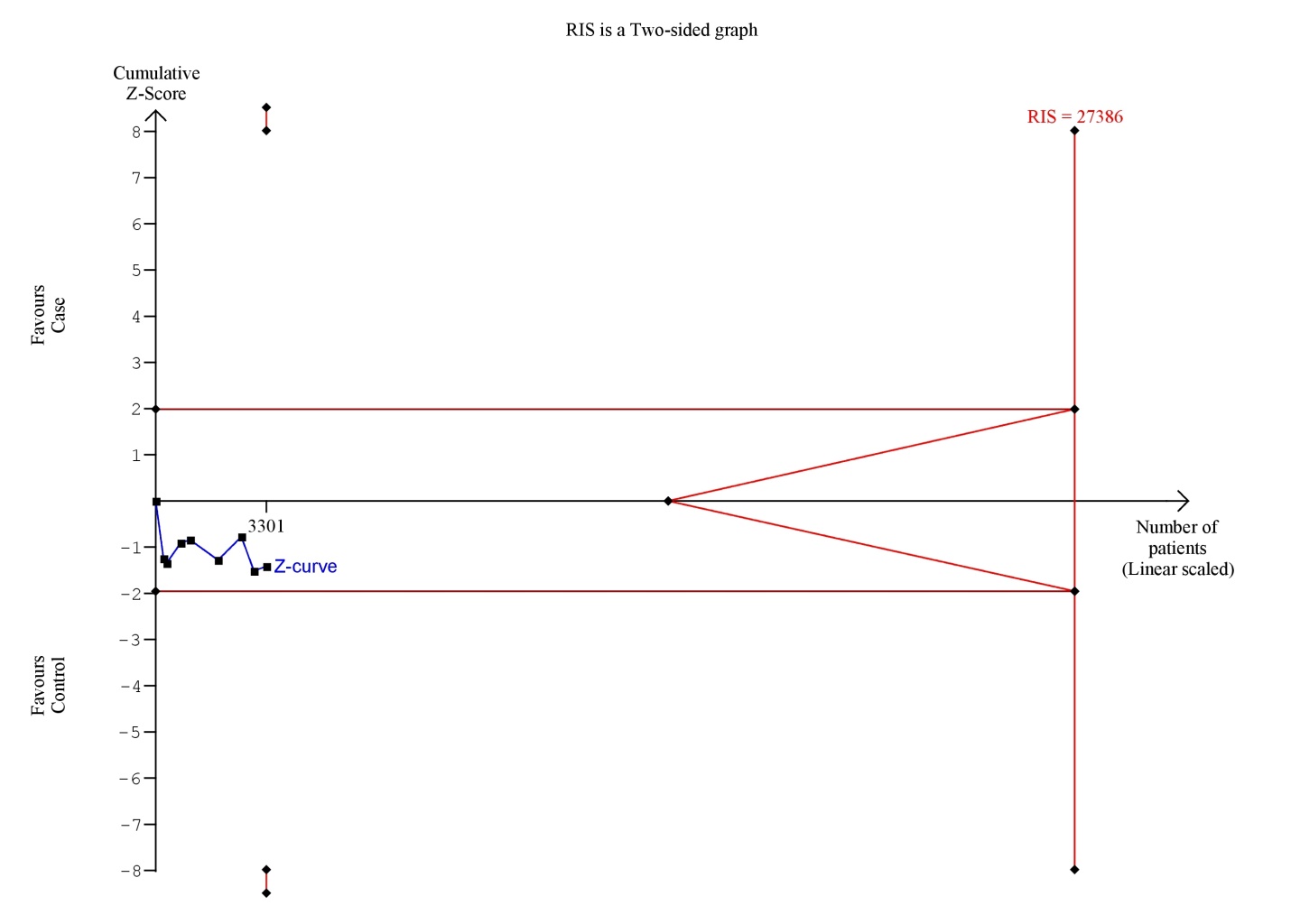


**Figure 29S**: Trial sequential analysis of the association between *XRCC1 Arg280His (rs25489)* polymorphism and the risk of leukemia in dominant model


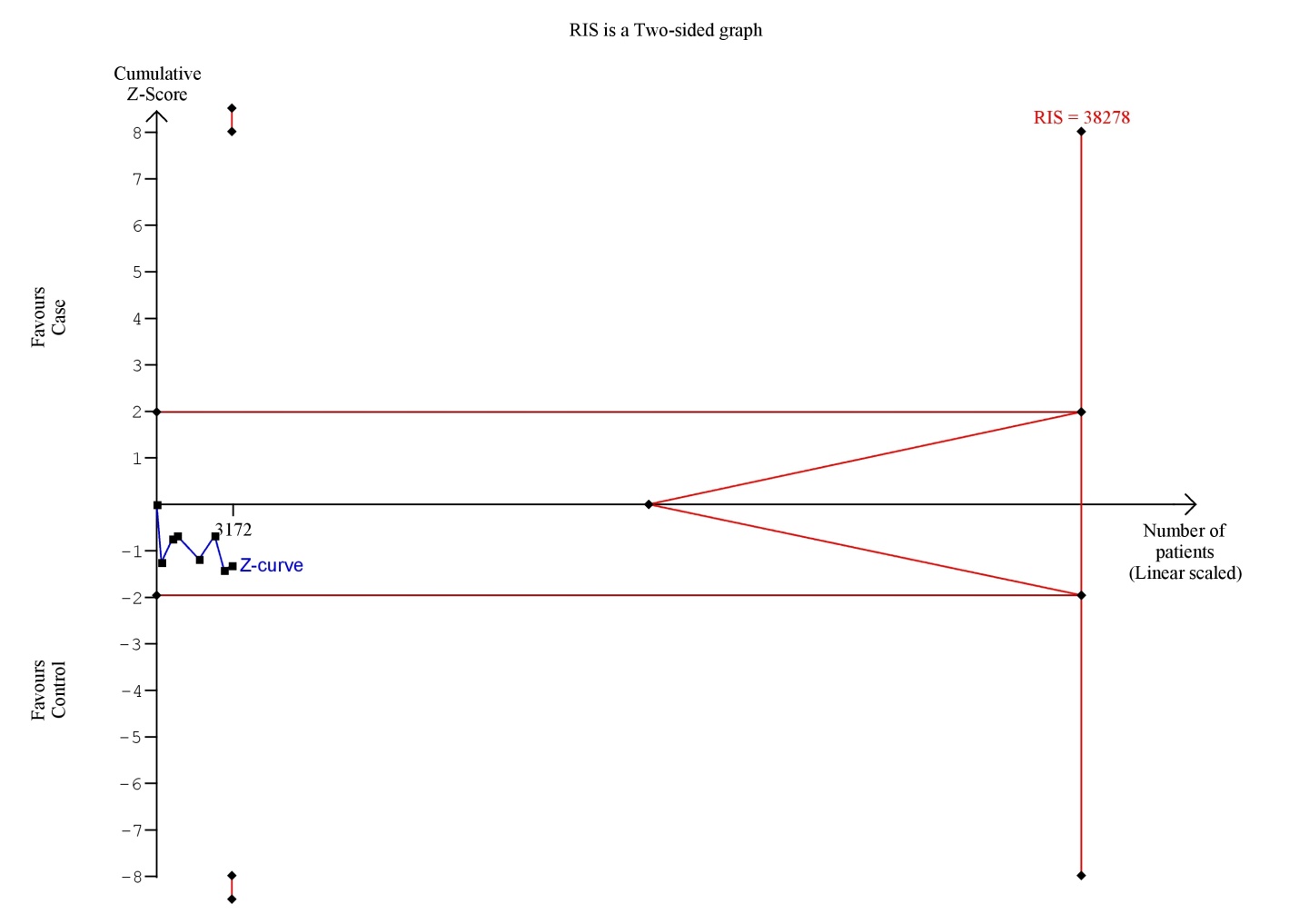


**Figure 30S**: Trial sequential analysis of the association between *XRCC1 Arg280His (rs25489)* polymorphism and the risk of leukemia in recessive model


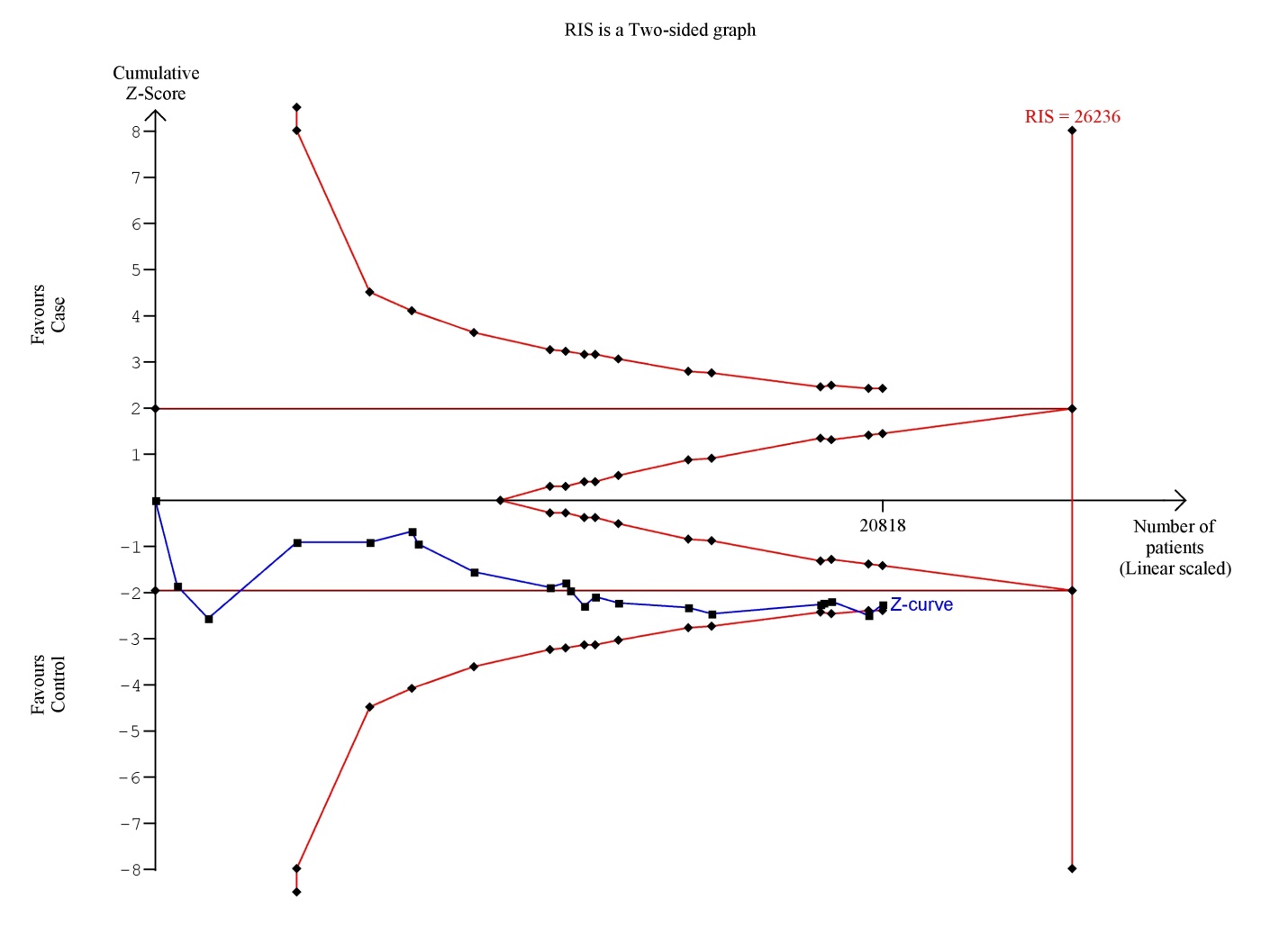


**Figure 31S**: Trial sequential analysis of the association between *XRCC3 Thr241Met (rs861539)* polymorphism and the risk of leukemia in allelic model


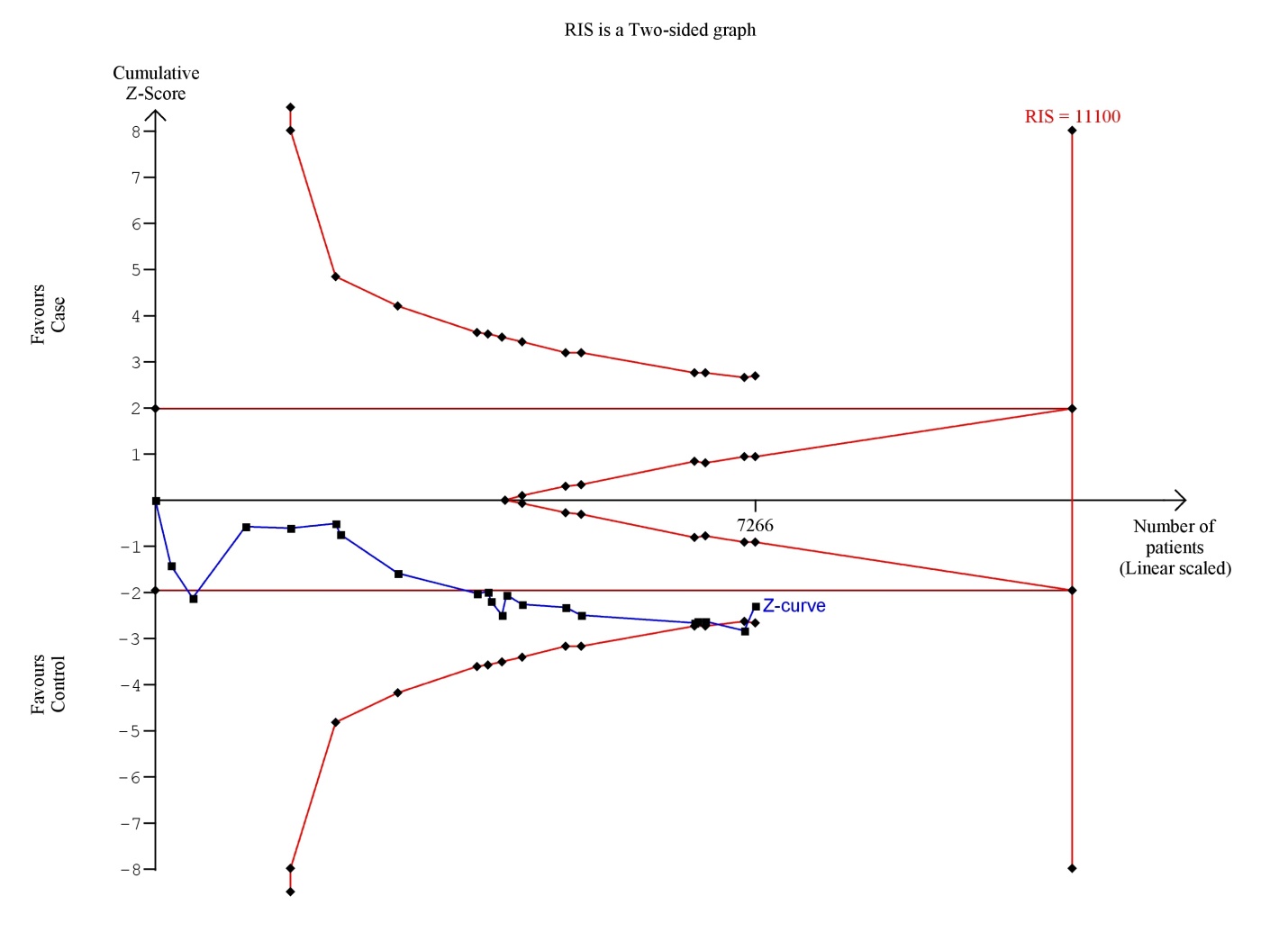


**Figure 32S**: Trial sequential analysis of the association between *XRCC3 Thr241Met (rs861539)* polymorphism and the risk of leukemia in homozygous model


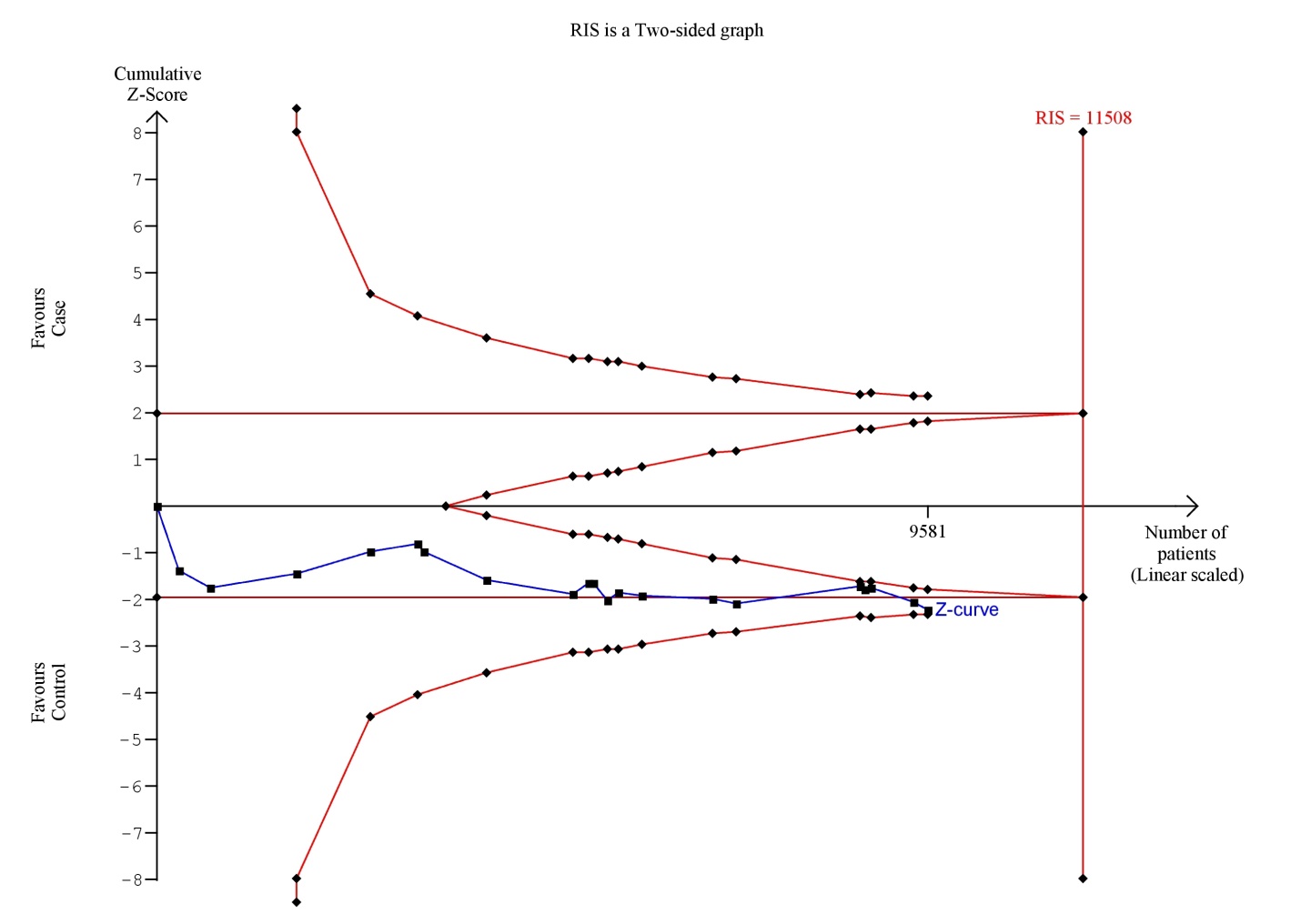


**Figure 33S**: Trial sequential analysis of the association between *XRCC3 Thr241Met (rs861539)* polymorphism and the risk of leukemia in heterozygous model


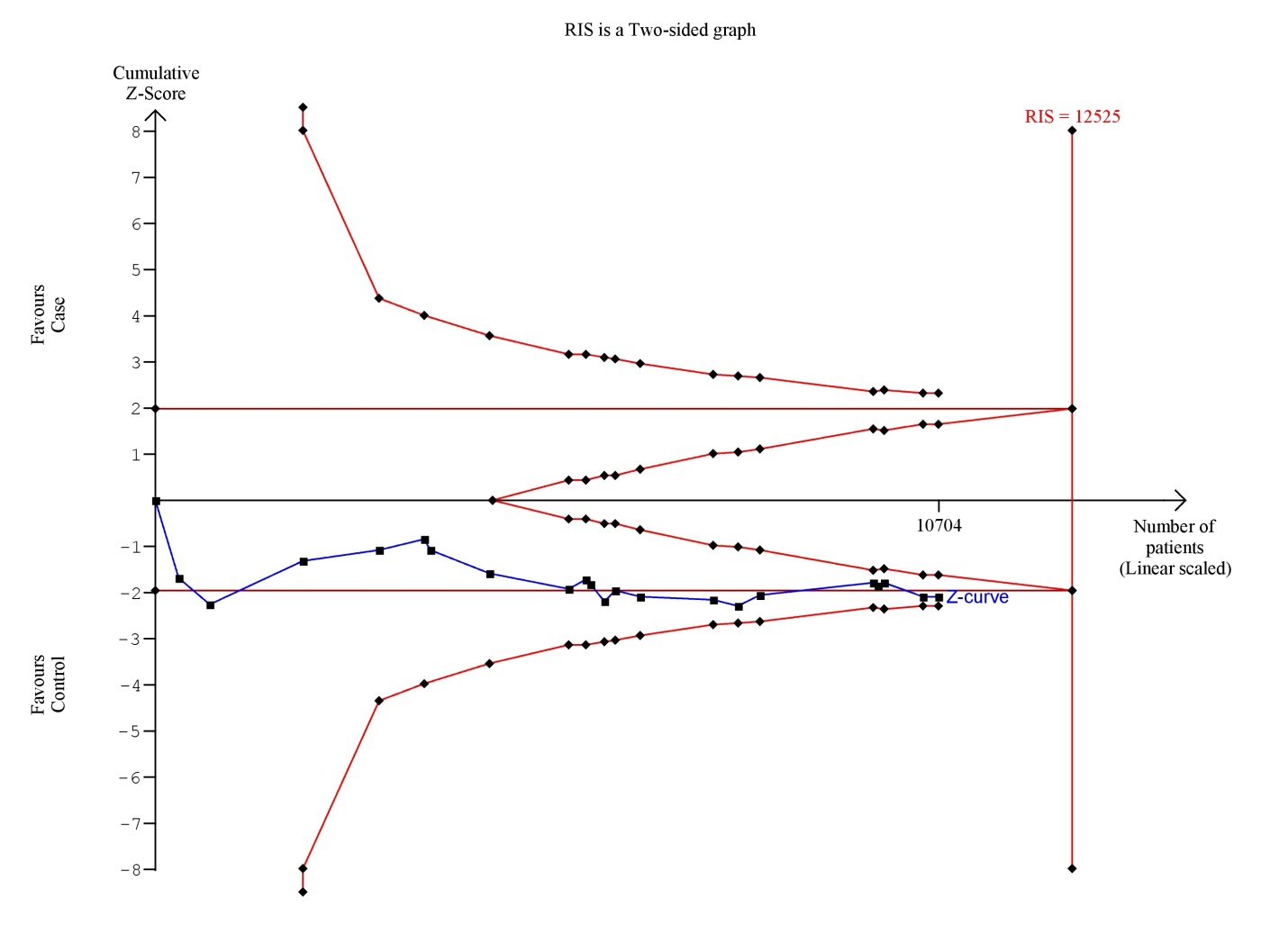


**Figure 34S**: Trial sequential analysis of the association between *XRCC3 Thr241Met (rs861539)* polymorphism and the risk of leukemia in dominant model


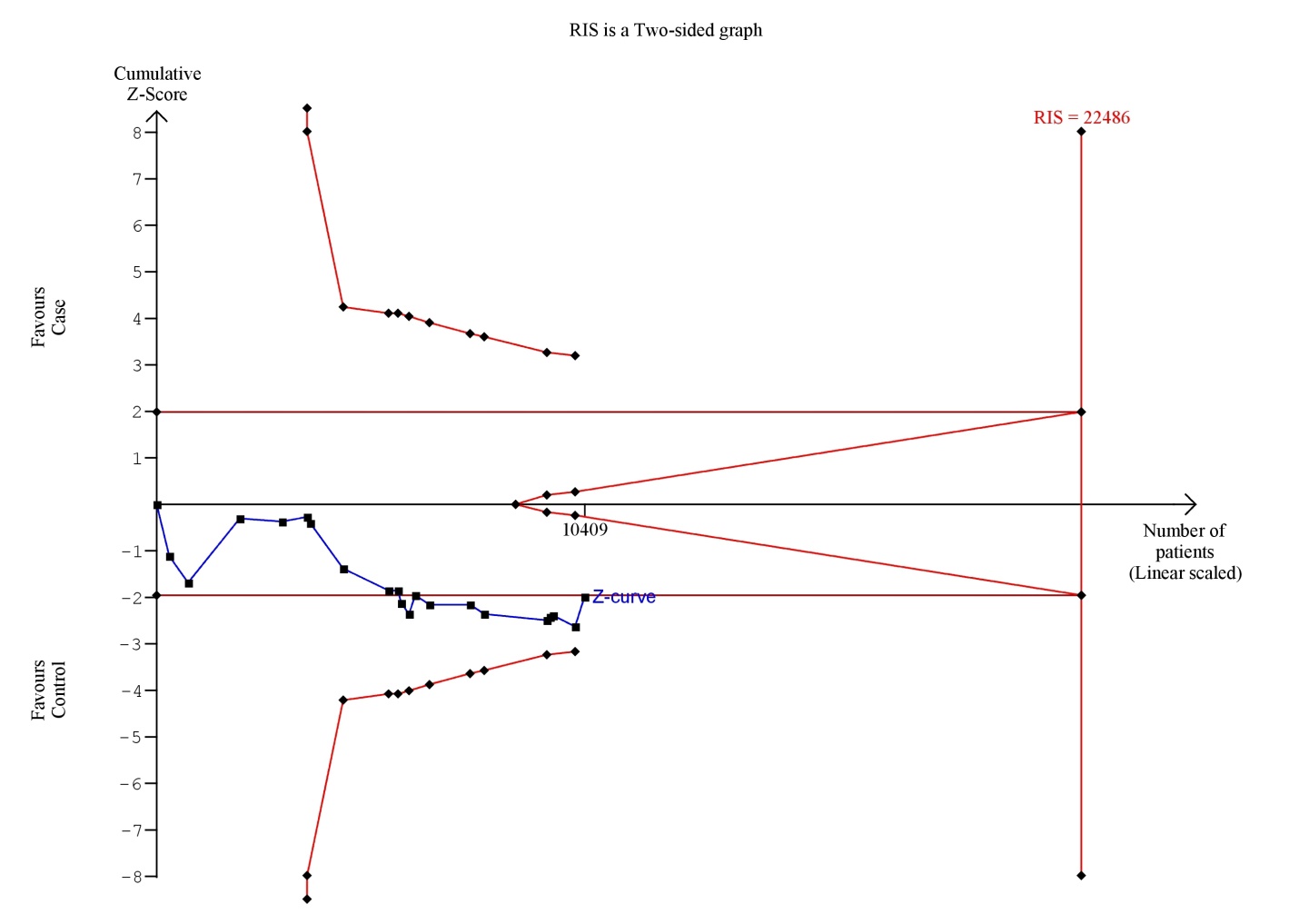


**Figure 35S**: Trial sequential analysis of the association between *XRCC3 Thr241Met (rs861539)* polymorphism and the risk of leukemia in recessive model
