# Supplementary material for: A meta-analysis and expression profiling of DNA repair gene polymorphisms in leukemia
Source: Front Oncol. 2026 Apr 23;16:1777198. doi: 10.3389/fonc.2026.1777198 (PMC13149071; doi:10.3389/fonc.2026.1777198)
Supplement: Supplementary file 4 [file DataSheet4.docx]

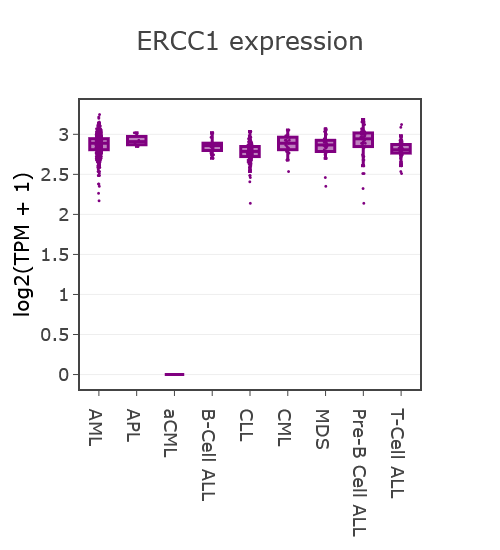


**Figure S1**: ERCC1 expression based on leukemia subtypes


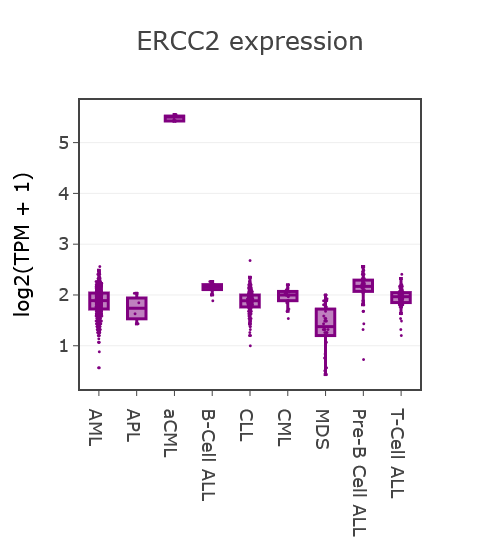


**Figure S2**: ERCC1 expression based on leukemia subtypes


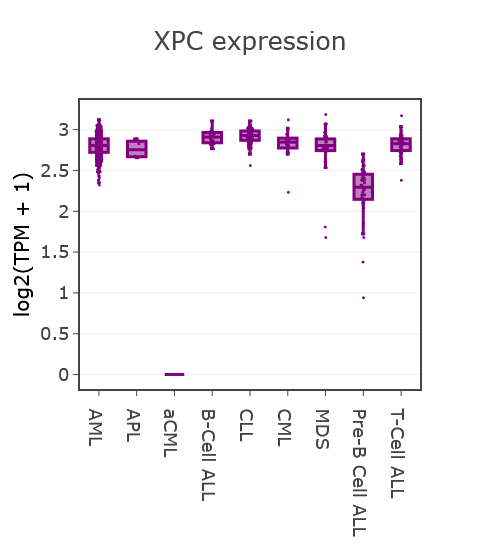


**Figure S3**: XPC expression based on leukemia subtypes


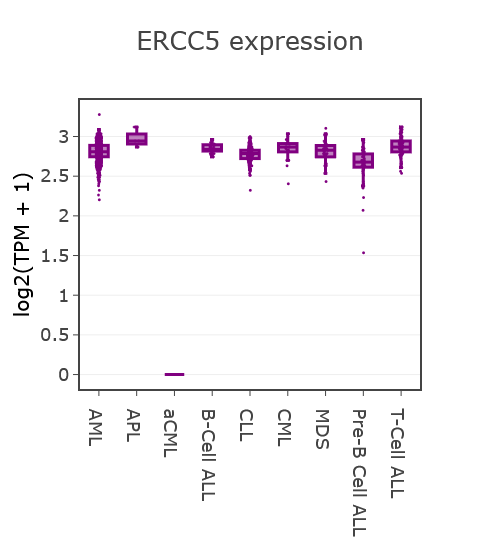


**Figure S4**: ERCC5 expression based on leukemia subtypes


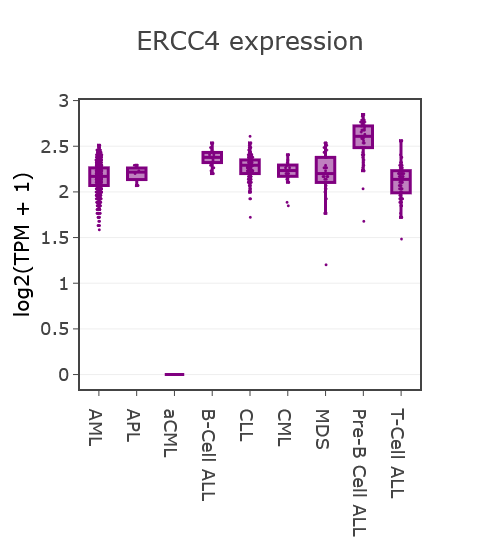


**Figure S5**: ERCC4 expression based on leukemia subtypes


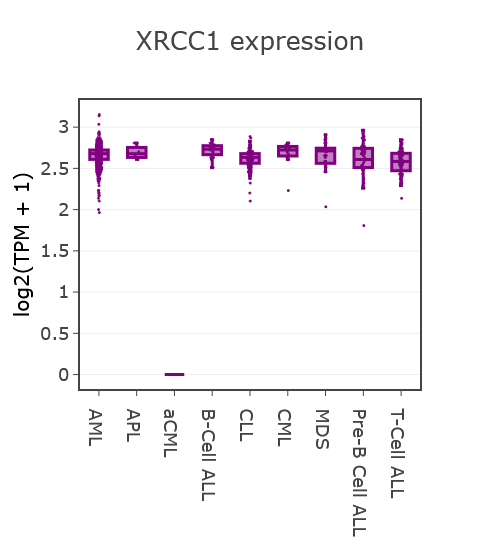


**Figure S6**: XRCC1 expression based on leukemia subtypes


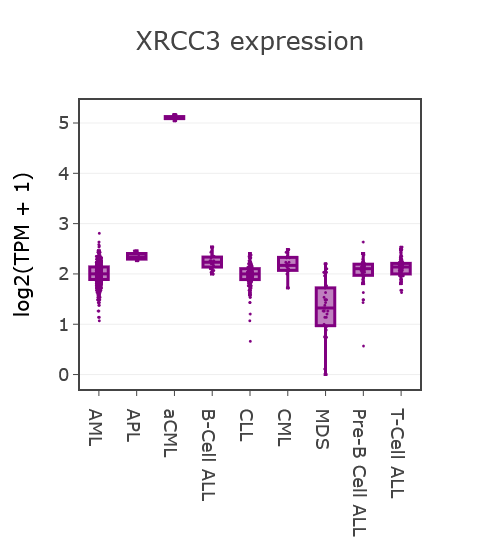


**Figure S7**: XRCC3 expression based on leukemia subtypes


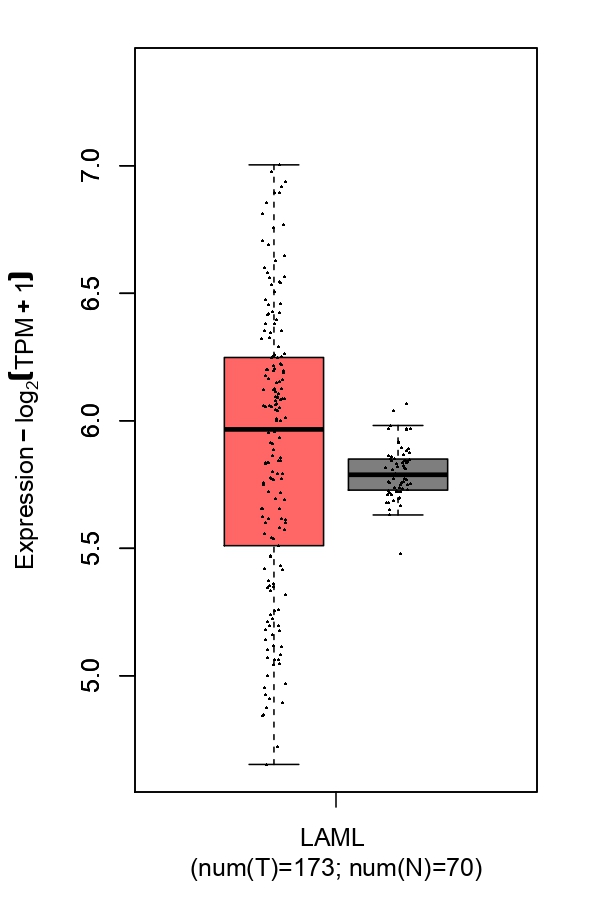


**Figure S8**: ERCC1 expression based on acute myeloid leukemia


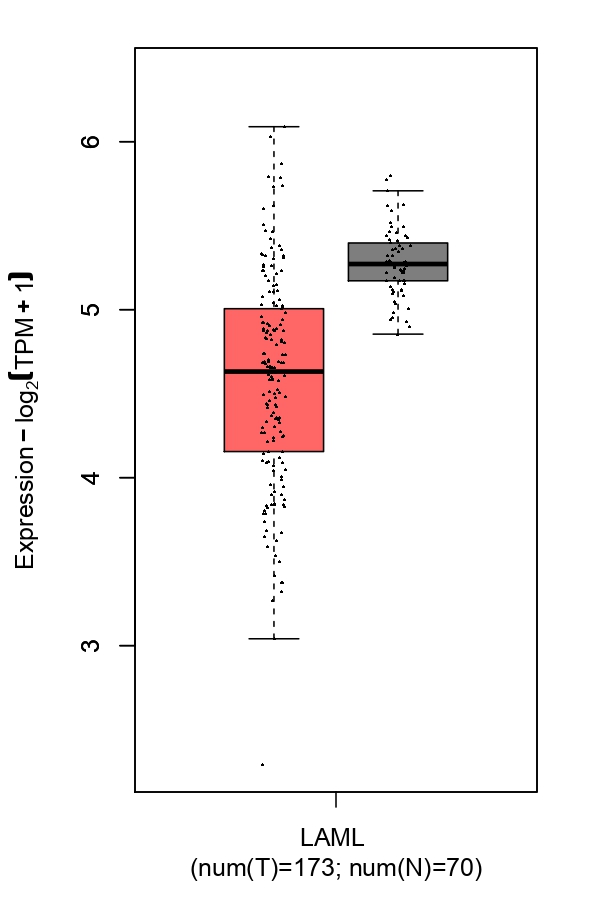


**Figure S9**: ERCC2 expression based on acute myeloid leukemia


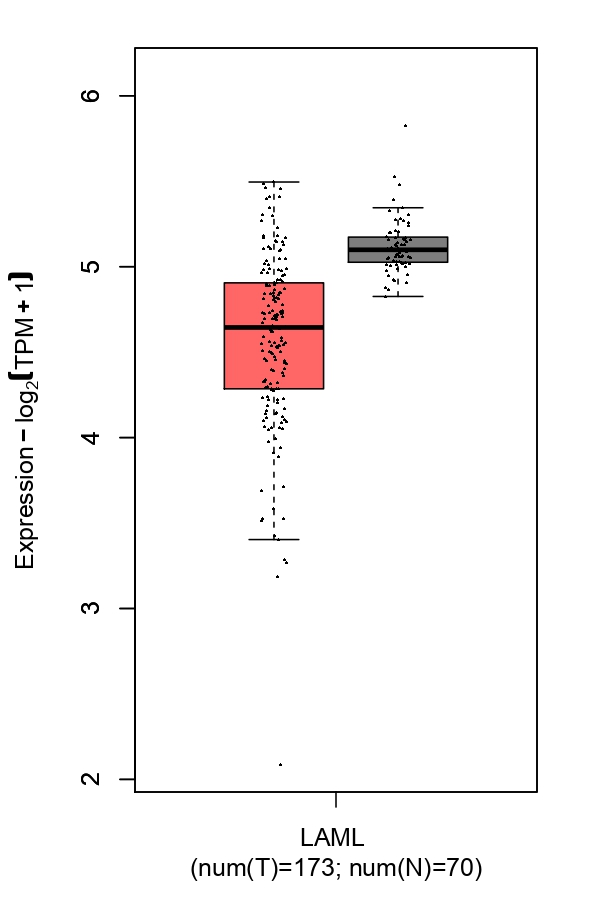


**Figure S10**: XPC expression based on acute myeloid leukemia


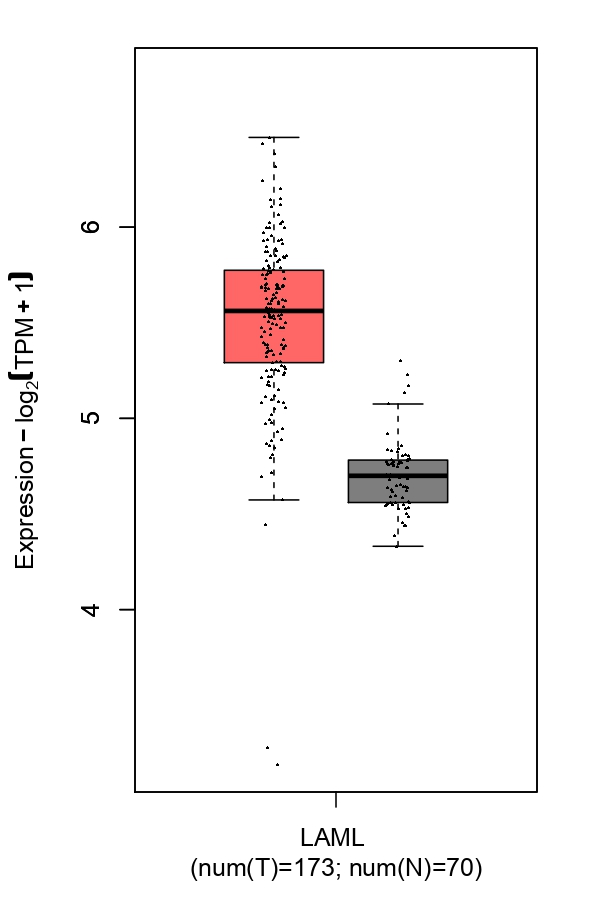


**Figure S11**: ERCC5 expression based on acute myeloid leukemia


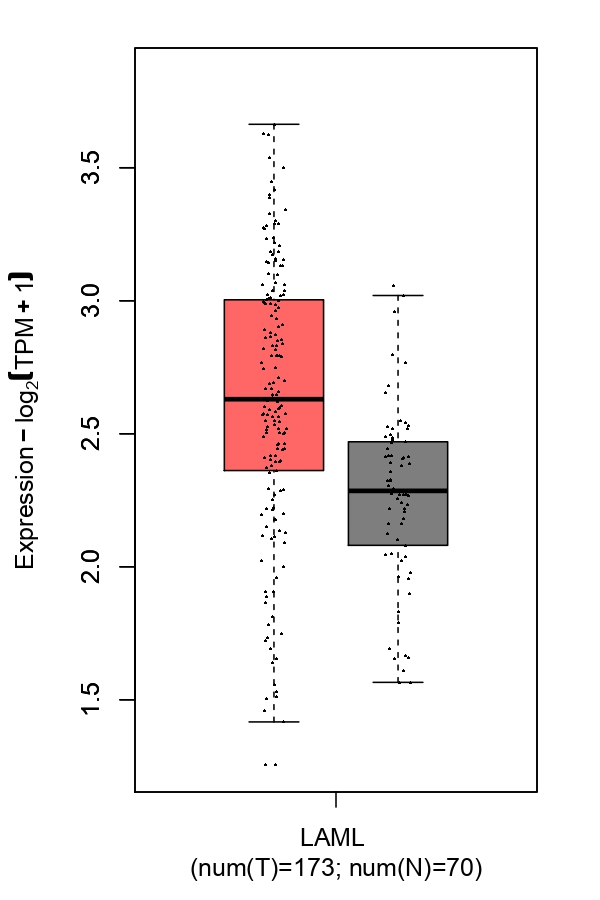


**Figure S12**: ERCC4 expression based on acute myeloid leukemia


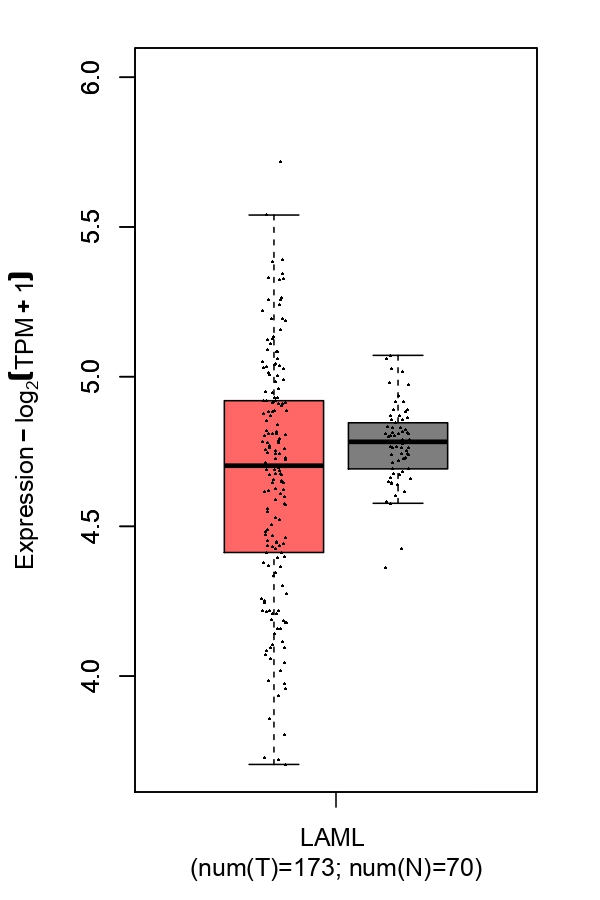


**Figure S13**: XRCC1 expression based on acute myeloid leukemia


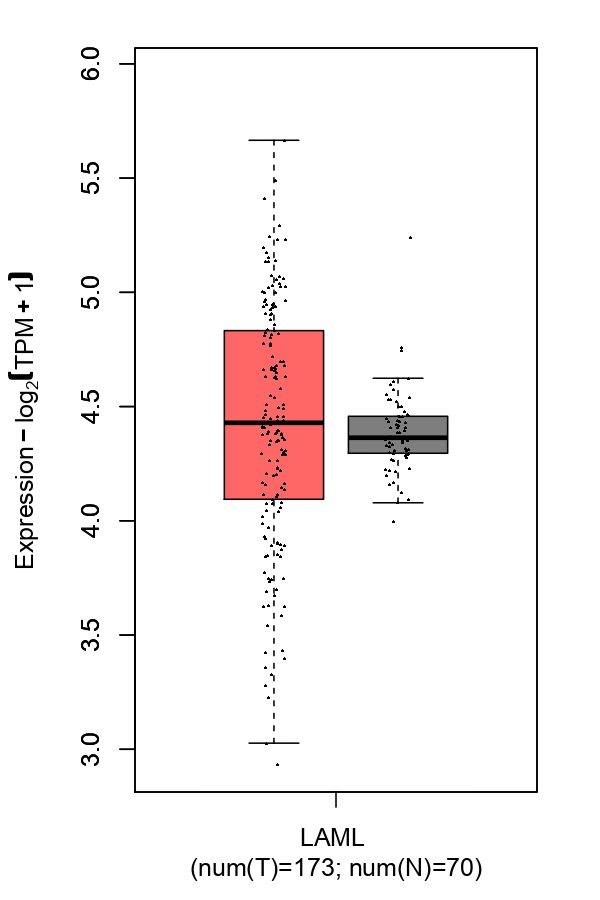


**Figure S14**: XRCC3 expression based on acute myeloid leukemia
